# Classification and metabolomic profiling of walnut pellicle polyphenols using a Pseudotargeted metabolomics approach

**DOI:** 10.1016/j.fochx.2026.103610

**Published:** 2026-01-30

**Authors:** Chang Liu, Mingxue Geng, Jiaxin Yin, Huibo Zhao, Bing Qi, Huiqing Li, Di Wang, Yanbing Wu, Shengxing Dai, Min Lu, Kuizhang Yao, Junxia Xia, Jiankang Deng

**Affiliations:** a School of Life Science and Food Engineering, Huaiyin Institute of Technology, Huai'an 223003, China; b College of Life Science, Hengshui University, Hengshui 053000, China; c Hebei Yangyuan Zhihui Beverage Co., Ltd., Hengshui 053000, China; d Hebei Key Laboratory of Walnut Nutritional Function and Processing Technology, Hengshui 053000, China; e Hebei Technology Innovation Centre of Walnut Beverage, Hengshui 053000, China; f College of Food Science & Technology, Shanghai Ocean University, Shanghai 201306, China

**Keywords:** Walnut pellicle, Polyphenols, Pseudotargeted metabolomics, Regional discrimination, Pathway enrichment, Food authenticity

## Abstract

Walnut pellicle is rich in polyphenols that enhance antioxidant capacity and health benefits, including anti-inflammatory and neuroprotective effects. Profiling these compounds has been hindered by their structural diversity, regional variability, and the limitations of traditional metabolomics approaches. This study employed a pseudotargeted metabolomics strategy, integrating ultra-high-performance liquid chromatography-quadrupole time-of-flight mass spectrometry (UHPLC-QTOF-MS) for untargeted profiling and multiple reaction monitoring (MRM) on a QTRAP system for semi-quantification. We analyzed 21 walnut pellicle samples from Xinjiang, Yunnan and the Taihang Mountains, identifying 406 polyphenols, including flavonoids, hydrolysable tannins, phenolic acids, coumarins, lignans and minor constituents. Multivariate analyses (PCA, PLS-DA, OPLS-DA) revealed region-specific metabolic fingerprints. KEGG pathway enrichment highlighted significant variations in flavonoid and phenylpropanoid biosynthesis across regions, with origin-specific markers like casuarinin, prunin, and ε-viniferin supporting provenance authentication. This research bridges the methodological gap in walnut polyphenol analysis and informs quality assurance, targeted breeding, and functional product development in sustainable food systems.

## Highlights

• Walnut pellicle polyphenols were analyzed by pseudotargeted metabolomics method, integrating UHPLC-QTOF-MS and LC-QTRAP-MS.
• A total of 406 polyphenolic compounds were tentatively identified and systematically classified.
• Multivariate analyses (PCA, PLS-DA, OPLS-DA) revealed region-specific metabolic fingerprints.
• KEGG enrichment analysis revealedsignificant regional differences in flavonoid and phenylpropanoid biosynthesis.

## 1 Introduction

Walnut (*Juglans regia L.*) is recognized as one of the four major nut crops worldwide, valued for its nutritional and economic significance. China stands as the largest global producer, with extensive cultivation across diverse agroecological zones. According to the China Statistical Yearbook, the primary walnut-producing regions are Yunnan, Xinjiang, and the Taihang Mountain range, the latter encompassing parts of Shanxi, Henan, and Hebei provinces (Liu et al., 2023).

Polyphenols, renowned for their antioxidant, anti-inflammatory, and neuroprotective properties (Vu et al., 2020), are abundant in walnuts and contribute substantially to their health-promoting benefits by scavenging free radicals, modulating inflammatory pathways, and protecting neuronal cells. Extensive research has characterized the major phenolic compounds in different parts of the walnut, including the kernel, oil, and residue fractions, thereby highlighting the complexity and richness of walnut polyphenols (Sheng et al., 2021; Slatnar et al., 2015). Among the diverse polyphenolic constituents, ellagitannins, ellagic acid, and flavonoids stand out as the primary contributors to the health benefits associated with walnut consumption (Sheng et al., 2021; Vu et al., 2018; Vu et al., 2020). However, the content and composition of these polyphenols vary markedly depending on cultivar, maturity, and growing environment (Huang et al., 2022; Johnson et al., 2016), underscoring the need to elucidate how genetic and ecological influences shape polyphenolic profiles.

The walnut pellicle, or inner seed coat, is particularly enriched in polyphenols and plays a pivotal role in the overall antioxidant capacity and sensory properties of the nut. Nevertheless, the comprehensive identification and quantification of these polyphenols remain challenging due to their complex chemical structures and large dynamic range in abundance. To address these issues, advanced analytical techniques such as high-performance liquid chromatography (HPLC), ultra-performance liquid chromatography coupled with mass spectrometry (UPLC-MS), and nuclear magnetic resonance (NMR) spectroscopy are commonly employed for the qualitative and quantitative analysis of walnut polyphenols. These methods provide detailed insights into the polyphenol diversity and their potential metabolic impacts on human health.

From an applied perspective, mapping regional variability in walnut polyphenol composition is increasingly important for the nut industry (Mo et al., 2022; Schmitt et al., 2020). Region-specific polyphenol signatures influence not only the nutritional value and antioxidant capacity of raw walnuts, but also the colour, bitterness/astringency, and shelf-life of downstream products such as walnut beverages and oils (Mo et al., 2022). Systematic knowledge of geographical polyphenol patterns can support quality assurance and raw material grading, guide targeted breeding and germplasm selection for desired health and sensory traits, and strengthen supply-chain integrity by enabling chemical authentication of geographical origin (Mo et al., 2022; Schmitt et al., 2020). In the longer term, such “polyphenol terroir” maps provide an evidence base for region-specific branding and the protection of high-value production areas.

From a fundamental plant metabolomics standpoint, walnut pellicles offer an informative model for studying tissue-specific polyphenol biosynthesis and its modulation by environmental gradients (Wang et al., 2022). Regional comparisons across major production areas capture coordinated shifts in ellagitannins, flavonoids, and other phenolic subclasses that reflect both genetic background and local climate/soil conditions. High-resolution mapping of this chemodiversity extends current provenance-focused metabolomics beyond traditional model species and contributes to a broader understanding of how perennial woody crops adapt their secondary metabolism to distinct ecological niches.

Building on these techniques, metabolomics—a rapidly evolving field in systems biology—offers a powerful approach to understanding the biochemical composition and physiological changes in response to dietary interventions, such as walnut consumption (Johnson et al., 2016). Metabolomics studies encompass targeted, untargeted, and hybrid approaches: untargeted metabolomics enables the discovery of a wide range of known and unknown metabolites without predefined targets, making it an ideal choice for exploratory research, though its data complexity often hinders metabolite identification and quantification. To bridge the gap between targeted and untargeted approaches, pseudotargeted metabolomics, which has emerged as a hybrid tool (Fan et al., 2022), combines the quantitative capabilities of targeted metabolomics with the broad coverage of untargeted analysis. This allows researchers to systematically quantify known polyphenols while also capturing novel, previously uncharacterized metabolites within specific metabolic pathways.

Despite significant progress in walnut polyphenol research, studies employing pseudotargeted metabolomics to investigate walnut pellicle remain notably absent. While untargeted metabolomics has been utilized to identify bioactive phenolics in walnuts (Vu et al., 2020; Zeb et al., 2024), and targeted methods have been developed for profiling polyphenols (Persic et al., 2018), the application of a high-coverage pseudotargeted approach to map regional variability in walnut pellicle polyphenols has not been reported. This gap emphasizes the need for research that leverages pseudotargeted metabolomics to deliver a more comprehensive and precise analysis of pellicle polyphenolic profiles across key production areas.

In response to this gap, the present study employs a pseudotargeted metabolomics strategy to systematically profile polyphenols in 21 walnut pellicle samples from Xinjiang, Yunnan, and the Taihang Mountains. The objectives are threefold: (1) to identify and classify the polyphenolic compounds in walnut pellicles, (2) to elucidate region-specific metabolic differences and their potential environmental drivers, and (3) to establish chemical markers for provenance authentication. By integrating multivariate statistical analyses (PCA, HCA, PLS-DA, OPLS-DA) and KEGG pathway enrichment, we aim to uncover the structural diversity, functional implications, and biosynthetic underpinnings of walnut polyphenols. This regional mapping provides actionable chemical markers for authentication and quality assurance, informs sourcing and breeding decisions in the walnut industry, and contributes to the broader field of plant metabolomics by offering a detailed case study of geographical chemodiversity in a perennial nut crop.

## 2 Materials and methods

### 2.1 Samples and chemicals

Walnut samples were sourced from commercial suppliers and local markets in three major walnut-producing regions of China: Yunnan, Xinjiang, and the Taihang Mountain area (covering parts of Shanxi, Hebei, and Henan). Within each region, independent commercial lots were obtained from different suppliers/markets to cover the major locally traded cultivars. Per-sample metadata, including cultivar/local genotype, origin (county/prefecture), and harvest period for each lot, are summarized in Table S1. In total, 8 lots were collected from the Taihang region (BF), 6 from Yunnan (YN), and 7 from Xinjiang (XJ), and each lot corresponded to an independent commercial sample. The cultivars listed in Table S1 are widely planted and traded in their respective regions. HPLC-grade acetonitrile, methanol, and formic acid were obtained from Merck (Darmstadt, Germany). Multiple non-phenolic internal standards (Table S2) were spiked at constant levels into all samples and QC pools. Ultra-pure water was generated using a Milli-Q system (Millipore, Bedford, MA, USA).

### 2.2 Polyphenol extraction and quality control samples

After immersion in liquid nitrogen, the walnut kernels were manually peeled to remove the pellicles. The pellicles were ground into a fine powder using a cryogenic mill. 100 mg of pellicle powder was extracted three times with 5 mL of methanol/water (80:20, *v*/v) containing 0.1% formic acid. Each extraction involved sonication for 30 min followed by centrifugation at 12,000 rpm for 10 min at 4 °C. The combined supernatant was filtered through a 0.22 μm membrane, freeze-dried, and stored at −80 °C until analysis.

To ensure consistency in the extraction process, the quality control (QC) sample was prepared by blending equal amounts of all pellicle extracts dissolved in methanol, creating a composite sample representative of all metabolites in the study. This QC sample facilitated method development, comparison, and quality assurance throughout the analytical workflow. Multiple internal standards were used to monitor extraction and injection reproducibility, track LC-MS/MS performance over time, correct retention time deviations between UHPLC-HRMS and QTRAP runs, and normalize MRM peak areas.

### 2.3 Data acquisition

#### 2.3.1 Untargeted profiling with UHPLC-HRMS

Building on a previously established pseudotargeted metabolomics protocol (Zheng et al., 2020), the overall UHPLC–HRMS acquisition and data-processing strategy in this study was adapted, with minor modifications, for walnut pellicle polyphenols. In brief, 21 samples were first analyzed using an untargeted metabolomics approach on a Waters ACQUITY UHPLC system coupled to an AB Sciex TripleTOF 5600 mass spectrometer to obtain a comprehensive profile of walnut pellicle polyphenols. The order of individual biological samples within the batch was randomized to minimize potential confounding by run order. All technical replicates of a given extract were kept in close proximity within the sequence but embedded within the randomized order of samples from different regions. The QC samples were regularly injected every 5 samples during the batch.

The HRMS system was equipped with an electrospray ionization (ESI) source. Chromatographic separation was performed on a Kinetex C18 column (100 mm × 2.1 mm, 2.6 μm, Phenomenex Corp.), maintained at 25 °C, and utilizing both positive and negative ion modes under identical conditions. The injection volume was set at 10 μL, with a flow rate of 0.50 mL/min. The mobile phases consisted of solvent A (0.1% formic acid in water) and solvent B (acetonitrile). The gradient elution program was as follows: 0–20 min, 95% to 70% A; 21–30 min, 70% to 10% A; 31–34 min, 10% A; 35–36 min, 10% to 95% A; and 37–40 min, 95% A. After gradient elution, the column was re-equilibrated to the initial conditions. The effluent from the column was directed to the mass spectrometer for data acquisition.

The mass spectrometric analysis was performed using a heated electrospray ionization (HESI) source in both positive and negative ion modes. In the positive ion mode, a spray voltage of 3.5 kV was applied, while the negative ion mode used a spray voltage of −3.0 kV. The capillary temperature was maintained at 320 °C, with the auxiliary gas heater temperature set at 350 °C. Sheath gas and auxiliary gas flow rates were set at 35 and 8 arbitrary units, respectively, for both ionization modes. The S-lens RF level was adjusted to 50. The mass range was scanned from *m*/*z* 70 to 1050, with a resolution of 70,000 for the full MS scan and 17,500 for MS/MS. The top five most intense ions (TopN = 5) were selected for fragmentation using stepped normalized collision energies (NCE) of 20 and 40. Additionally, the collision energy (CE) for the positive ion mode was set to 15, 30, and 40 V, whereas for the negative ion mode, it was set to −15, −30, and − 40 V. Both ionization modes utilized an information-dependent acquisition (IDA) method to perform secondary ion scanning, ensuring high-quality fragmentation data for subsequent metabolite identification. This untargeted UHPLC-HRMS dataset was then used to construct a high-coverage pseudotargeted MRM panel for semi-quantitative monitoring of walnut pellicle polyphenols, following the general strategy of Zheng et al. (2020).

#### 2.3.2 Selection of MRM ion-pairs and MRM transitions

Raw UHPLC-HRMS data were converted into MGF format using MSConvert software. Subsequently, XCMS was utilized for peak detection, identifying metabolite features. Peak detection was performed with XCMS, followed by peak annotation using CAMERA to group ions by retention time and adduct patterns, identifying *m*/*z* values, isotopes and adducts. Redundant features were filtered to prepare for multiple reaction monitoring (MRM) transition selection.

MRM ion-pairs were selected using MRM-Ion Pair Finder software (version 2.0), which processes MS/MS spectra to identify optimal precursor-product ion pairs based on ion intensity, stability, and minimal interference. Candidate ion-pairs were validated by analyzing precursor ion MS/MS spectra to select intense, specific product ions for targeted MRM semi-quantification. Consistent with pseudotargeted metabolomics workflows (Zheng et al., 2020), MRM peak areas were normalized to internal standards (Table S2) and reported as relative abundances; absolute concentrations were not determined in this study. Normalized peak areas were subsequently used for multivariate statistical analysis and visualization. The ion-pair list was refined using prior knowledge of polyphenol-targeted assays, as detailed previously (Luo et al., 2015). Key parameters, including retention time, *m*/*z* values, and signal intensity, were optimized for specificity and reproducibility. Collision energies were fine-tuned to maximize sensitivity and minimize background noise. Final MRM transitions were validated for retention time consistency across multiple runs.

Retention time discrepancies between UHPLC-HRMS and UHPLC-Triple Quadrupole MS (TQMS) were corrected using internal standards (Cholic acid-2,2,4,4-d4, Chenodeoxycholic acid-2,2,4,4-d4, Decanoyl-l-carnitine-d3 HCl (*N*-methyl-d3), Hexadecanoyl-l-carnitine-d3 HCl (*N*-methyl-d3), Nonadecanoyl-2-hydroxy-sn-glycero-3-phosphocholine, *L*-phenylalanine (Ring-d5), L-tryptophan-d5 (Indole-d5)). Their retention times, ionization conditions, and ion transitions are detailed in Table S2. These standards served as references to adjust metabolite retention times, enhancing MRM transition accuracy and reproducibility.

#### 2.3.3 Pseudotargeted analysis with QTRAP

Pseudotargeted metabolomics was conducted using an AB Sciex QTRAP 5500 LC/MS/MS system in dynamic MRM mode. Chromatographic conditions mirrored those of UHPLC-HRMS, except for a 5 μL injection volume. The QTRAP operated with ion source gas 1 and 2 at 50 psi, curtain gas at 35 psi, and an ESI source temperature of 500 °C. Ion spray voltages were 5500 V (ESI+) and − 4500 V (ESI-).

#### 2.3.4 Metabolite annotation and MSI levels

Metabolite annotation followed the Metabolomics Standards Initiative (MSI) guidelines. Because no authentic reference standards were analyzed under the present UHPLC-MS conditions, all polyphenolic features are reported at MSI Levels 2–4. Vendor software annotates features using three database tiers, reported as “Databases Level”: a Standards database compiled from authentic reference standards (Databases Level 1), the KEGG compound database (Databases Level 2), and an Integrated database combining multiple public resources (Databases Level 3). For each feature, the software also reports MS/MS-based scores (MS1, forward and reverse MS2 scores and MS2 correlation), which summarize the type and quality of database evidence.

In this study, we used “Databases Level” together with the presence or absence of MS/MS spectral matching to assign MSI levels. Features with high-quality MS/MS spectra and a positive spectral match in any of the three databases were classified as MSI Level 2 (putatively annotated compounds), whereas features supported only by MS1 information (exact mass, isotopic pattern and/or molecular formula) without reliable MS/MS confirmation were classified as MSI Level 3 (putatively characterized compound classes). Signals without any structural assignment were designated as MSI Level 4 (unknown features). Although the Standards database is built from authentic compounds, in our workflow it was used only as an external library; no reference standards were injected together with the walnut samples, and therefore no feature is reported at MSI Level 1. The MSI level, together with precursor/product ions, retention time, “Databases Level” and MS/MS evidence, is summarized for all 406 features in Tables S3 and S4.

### 2.4 Data processing and multivariate Modeling

For each walnut pellicle sample, a single extract was prepared and analyzed by LC–MS/MS in five technical replicate injections. Metabolite characterization employed a multilevel annotation system (Supplementary Material). The One-MAP platform (http://www.5omics.com/, version 1.0) integrated qualitative annotation results for preliminary metabolite identification. Multivariate statistical analyses, including principal component analysis (PCA), hierarchical cluster analysis (HCA), partial least squares-discriminant analysis (PLS-DA), and orthogonal partial least squares-discriminant analysis (OPLS-DA), were conducted using R (version 4.5.0) to elucidate variations in metabolite profiles among samples.

For pseudotargeted LC-MS/MS analysis, the final data matrix consisted of 406 MRM transitions (metabolites) measured across 21 walnut pellicle samples. Raw peak areas were first inspected for missing values. Metabolites with more than 20% missing entries across all samples were excluded from both multivariate and univariate analyses. For the remaining metabolites, sporadic missing values—typically corresponding to peaks below the detection threshold—were imputed using half of the minimum non-zero peak area observed for that metabolite across the dataset (x imputed = 0.5 × min {x > 0}).

To stabilize variance and reduce right-skewness, the imputed peak areas were log_10_-transformed as x’ = log_10_(x imputed +1). Before PCA, PLS-DA, and OPLS-DA, each metabolite was mean-centered and Pareto-scaled by dividing its centered values by the square root of its standard deviation. This scaling scheme is widely used in metabolomics and balances the contribution of medium- and high-abundance features. All multivariate analyses were performed on these log-transformed, Pareto-scaled, semi-quantitative relative abundances.

Unsupervised PCA was first applied to visualize overall similarities among samples and to assess potential outliers. Supervised PLS-DA and OPLS-DA models were then constructed to evaluate regional discrimination among the three origins (BF, YN, XJ). Models were built using the ropls package in R, with class membership (BF, YN, XJ) as the response. To assess model robustness and mitigate overfitting, a stratified 7-fold cross-validation scheme was employed and repeated 50 times with different random fold allocations. Stratification ensured that the class proportions (BF, YN, XJ) were preserved in each fold. At each repetition, PLS-DA models were trained on six folds and used to predict the held-out fold; predictions from all folds and repetitions were aggregated to construct an overall 3 × 3 confusion matrix, from which overall accuracy, balanced accuracy (macro-averaged recall across the three classes), and a macro-averaged Matthews correlation coefficient (MCC) were derived.

To further guard against overfitting, 200-label permutation tests were performed under the same 7-fold cross-validation regime. For each permutation, class labels were randomly shuffled while keeping the data matrix fixed, and PLS-DA models were refitted to generate empirical null distributions of R^2^ and Q^2^. The observed Q^2^ intercepts remained negative, indicating that the discrimination achieved by the original model was unlikely to arise from random chance.

### 2.5 Univariate statistical analysis and multiple-testing correction

For each metabolite, differences in relative abundance among the three regions (BF, YN, XJ) were evaluated using univariate statistical tests applied to the log_10_-transformed data. Normality and homoscedasticity were assessed using the Shapiro-Wilk and Bartlett tests, respectively. If both assumptions were met, a one-way ANOVA was performed; otherwise, the non-parametric Kruskal-Wallis test was used. The resulting *p*-values across all 406 metabolites were corrected for multiple testing using the Benjamini-Hochberg false discovery rate (FDR) procedure, yielding q-values. Metabolites with q < 0.05 were considered statistically significant in the global three-class comparison.

For pairwise regional comparisons (BF vs YN, BF vs XJ, YN vs XJ), log_2_ fold-changes (log_2_FC) in relative abundance were calculated from group means, and volcano plots were constructed using log_2_FC on the x-axis and -log_10_(q) on the y-axis. Metabolites were considered significantly different between two regions if they met both |log₂FC| > 0.585 (fold-change >1.5) and q < 0.05. Variable importance in projection (VIP) scores from the PLS-DA models were used to prioritise q-significant metabolites (VIP > 1.0) as candidate discriminant markers, rather than as an additional significance threshold.

## 3 Results and discussion

### 3.1 The pseudotargeted metabolomics process

**Fig. 1:**
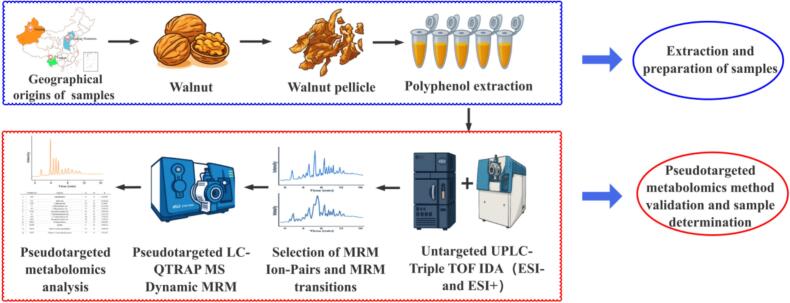
Flowchart of the sample-directed pseudotargeted metabolomics method.

In this study, we implemented a pseudotargeted metabolomics workflow based on liquid chromatography-tandem mass spectrometry (LC-MS/MS) to systematically profile the polyphenolic composition of walnut pellicles (Fig. 1). The geographic map in Fig. 1 presents the geographical origin of the walnut pellicles from 21 samples collected from three ecologically distinct regions: the Taihang Mountains (BF), Yunnan (YN), and Xinjiang (XJ). Sampling sites are marked with stars and colour-coded according to climate: orange (arid), green (high-plateau humid) and blue (temperate continental).

**Fig. 2:**
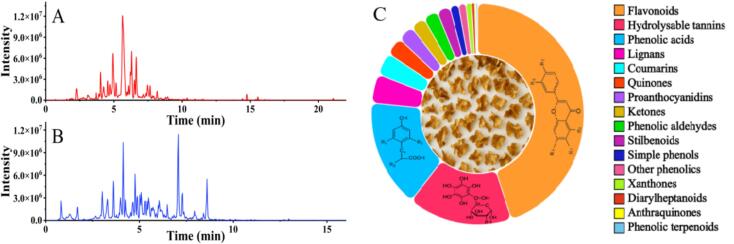
Typical TIC chromatograms of non-targeted metabolomics method based on UHPLC-Q-TOF-MS in (A) positive and (B) negative modes, (C) Pie chart showing the distribution of 406 putatively identified polyphenols by main class.

The workflow begins with exhaustive acquisition of precursor (Q1) and product (Q3) ion pairs across the metabolome. QC pools were first analyzed in untargeted mode on an ultra-high-performance LC coupled to a quadrupole time-of-flight mass spectrometer (UHPLC-QTOF-MS) operating in information-dependent acquisition (IDA). To maximize fragmentation coverage, spectra were recorded at collision energies of 15, 30 and 40 V in positive electrospray mode and − 15, −30 and − 40 V in negative mode. Representative total ion chromatograms (TICs) from ESI+ and ESI- modes of QCs are provided in Fig. 2 (A). Initially, 1658 ion pairs were detected in ESI+ mode and 1045 in ESI- mode. After deconvolution to remove isotopic redundancies, neutral loss peaks, and adduct ions, 1191 and 896 unique ion pairs, respectively, were retained to construct a high-confidence precursor-product ion dataset for MRM method development.

MS1 features were unevenly distributed across the *m*/*z* range, risking fragment overlap when fixed Q1 windows were used. We therefore adopted a TIC-guided variable-window strategy that equalises ion current among acquisition windows, improves MS2 spectral quality and ultimately yields more reliable MRM transitions. By integrating broad untargeted discovery on the QTOF with sensitive, selective quantification on a triple-quadrupole, the pseudotargeted approach combines the coverage of global profiling with the reproducibility of a targeted assay. The resulting platform affords accurate detection of low-abundance polyphenols and superior inter-sample comparability relative to conventional untargeted workflows, providing a robust basis for in-depth characterization of walnut pellicle metabolomes (Luo et al., 2016).

### 3.2 Comprehensive profiling of walnut pellicle polyphenols

The pseudotargeted LC–MS/MS workflow enabled comprehensive putative annotation of polyphenolic compounds from 21 walnut pellicle samples. In total, 293 and 135 unique compounds were detected in negative (ESI-) and positive (ESI+) ionization modes, respectively. After reconciling duplicates by retention time, precursor-product transitions, and MS/MS fragmentation patterns, we established a non-redundant catalogue of 406 polyphenolic features (MSI Levels 2–4) for structural classification and biological interpretation based on normalized, semi-quantitative MRM peak areas. These 406 features represent the complete set of peaks retained after quality-control filtering and were detected in all 21 biological samples, so regional differences arise from relative abundance patterns rather than simple presence/absence.

As shown in Fig. 2B, this phenolic profile is dominated by flavonoids (201 compounds), spanning diverse subclasses such as flavonols, flavones, flavan-3-ols, and flavanones. Phenolic acids (71 compounds) also comprise a major class, including hydroxybenzoic acids, hydroxycinnamic acids, and derivatives. A comparable number of hydrolysable tannins (71 compounds) were putatively annotated, mainly consisting of gallotannins, ellagitannins, and oxidized ellagitannins (dilactones). Additional major categories include lignans (19 compounds), proanthocyanidins (20 compounds), and coumarins (15 compounds). Minor classes include stilbenoids, phenolic aldehydes and ketones, quinones, xanthones, simple phenols, phenolic terpenoids, and diarylheptanoids. Notably, some compounds exhibit structural features that allow classification into more than one main class (e.g., flavonoid-tannin or flavonoid-phenolic acid hybrids), reflecting their multifunctional nature (Mutha et al., 2021). Negative ion mode offered superior sensitivity for acidic phenolics—especially phenolic acids and tannins—whereas positive ion mode enhanced the detection of flavonoid aglycones and other neutral compounds. This aligns with Regueiro et al. (2014), who identified more than 120 phenolic compounds in kernels using LC-LTQ-Orbitrap MS, describing fragmentation behaviours (e.g., CO_2_ losses, retro-Diels-Alder cleavages) that mirror those observed here. Our pseudotargeted approach extends this coverage to 406 compounds in pellicles, revealing additional classes such as lignans and providing the basis for the regional variability analyses presented in the subsequent multivariate and univariate sections.

At the semi-quantitative level, the 406 pellicle polyphenols exhibited a broad dynamic range of normalized MRM peak areas across the three regions, with many ellagitannins, flavan-3-ols and stilbenoids among the highest-abundance and most variable features. The detailed statistics on per-compound abundance and inter-regional differences (p- and q-values, fold-changes and VIP scores) are reported in Sections 3.4–3.5 and summarized in Table S8, where formal multivariate and univariate tests are used to quantify these regional patterns.

#### 3.2.1 Flavonoids

Flavonoids constituted a substantial proportion of pellicle polyphenols. Flavan-3-ols such as (+)-catechin and (+)-epicatechin were among the most abundant and appeared as [M–H]^+^ ions at *m*/*z* 289 and yielded characteristic fragments at *m*/*z* 245 (Sheng et al., 2021). These catechins are monomers of condensed tannins and contribute to the astringency of the walnut skin (Sheng et al., 2021). Galloylated derivatives—including (−)-catechin-3-O-gallate and procyanidin gallate dimers—were also identified (Sheng et al., 2021). Beyond their influence on taste and colour, flavonoids exhibit antioxidant, anti-inflammatory, and cardioprotective activities in vitro and in vivo (Mutha et al., 2021).

Flavonol glycosides were pervasive, with derivatives of quercetin, myricetin, kaempferol, and isorhamnetin predominating. Quercetin-3-glucoside showed a parent ion at *m*/*z* 465.1 and a fragment at m/z 304.1; myricetin 3-(2′′,3′′-digalloylrhamnoside) fragmented from m/z 769.1 to 335; and taxifolin fragmented from m/z 305.1 to 287.1. The abundance of these glycosylated flavonoids accords with the high total phenolic content of walnut pellicle and likely underpins its potent antioxidant capacity (Sheng et al., 2021).

#### 3.2.2 Hydrolysable tannins (ellagitannins and Gallotannins)

Ellagitannins and gallotannins, two subclasses of hydrolysable tannins, were also abundant. Ellagic acid itself appeared prominently at m/z 303 ([M-H]^−^) with fragments at m/z 286 arising from characteristic losses. Numerous ellagitannins were observed: casuarictin at m/z 937.1 produced fragments at m/z 919.1, while casuarinin at m/z 937.1 yielded fragments at m/z 345, and vescalagin at *m*/*z* 935.1 showed fragments at *m*/*z* 881. Peaks at m/z 483.1, 635.1, and 787.1 corresponded to di- through tetragalloyl-glucose, each showing successive 152 Da losses of galloyl units. Detection in ESI was favored by the multiple phenolic hydroxyls of these compounds. The rich ellagitannin/gallotannin profile—previously reported for walnut pellicle—contributes to antioxidant capacity and, after hydrolysis, supplies bioactive ellagic acid. The presence of these hydrolysable tannins was confirmed primarily in the negative ion data (Table S4), as their multiple phenolic hydroxyls favor deprotonation. This rich ellagitannin/gallotannin profile is in agreement with walnut's known composition; indeed, compounds like casuarictin, casuarinin, and galloylglucoses have been previously reported in walnut pellicle (Medic et al., 2021; Regueiro et al., 2014; Sheng et al., 2021). These tannins contribute to the pellicle's antioxidant properties and, upon hydrolysis (e.g., during digestion), release ellagic acid—a bioactive phenolic with anti-inflammatory and anticancer attributes.

#### 3.2.3 Phenolic acids

A variety of low-molecular-weight phenolic acids and their derivatives were putatively annotated, mostly via ESI- data (Table S4). Prominent among these were the hydroxybenzoic acids: 2,3-dihydroxybenzoic acid (*m*/*z* 153), 2,4-dihydroxybenzoic acid (m/z 153), 2,3,4-trihydroxybenzoic acid (m/z 169) and 4-*O*-methylgallic acid (m/z 183). Each of these showed the expected loss of CO_2_ (44 Da) in MS/MS, yielding a characteristic fragment (e.g., m/z 109 from 2,3-dihydroxybenzoic acid, m/z 168 from 4-*O*-methylgallic acid), which is a common fragmentation pathway for benzoic acid derivatives (Sheng et al., 2021). Among hydroxycinnamic acids, we detected caffeic acid (m/z 179) and trans-p-hydroxycinnamic acid (m/z 163) in the pellicle. Their identities were supported by typical fragments (e.g., caffeic acid yielding *m*/*z* 135 after loss of CO_2_). We also observed more complex conjugates of phenolic acids. Chlorogenic acid was tentatively identified with [M-H]^−^ at *m*/*z* 353.1, producing prominent fragments at m/z 192.1 (quinate anion) (Regueiro et al., 2014). Similarly, a coumaroylquinic acid (m/z 337 for [M-H]^−^) was annotated by fragment ions at m/z 191, consistent with loss of quinic acid and formation of a coumarate ion (Regueiro et al., 2014). We further detected hexosides of phenolic acids; for instance, 3-glucogallic acid (m/z 331 for [M-H]^−^) was present, exhibiting fragment ions (m/z 313.1) that correspond to losses from the sugar moiety (Regueiro et al., 2014). The suite of phenolic acids putatively identified is consistent with known walnut compositions and contributes to the overall antioxidant capacity of the pellicle. While generally less abundant than the tannins or flavonoids, these simple phenolics (and their glycosides) can influence the pellicle's flavor (some impart slight bitterness) and serve as important precursors in polyphenol biosynthetic pathways.

#### 3.2.4 Coumarins

Coumarin-type compounds were also detected in the walnut pellicle, albeit in minor quantities. We putatively identified a peak corresponding to a 7-hydroxycoumarin derivative, tentatively assigned as 4-methylumbelliferone (a methylated form of umbelliferone). This compound was observed in the negative ion mode with [M-H]^−^ at *m*/*z* 176, and its MS/MS spectrum showed fragment ions (e.g., m/z 173) characteristic of coumarin core cleavage. The presence of 4-methylumbelliferone (or an isomer thereof) suggests that walnut pellicle contains low levels of coumarins or related benzopyrone metabolites. Coumarins are a class of phenolics known for their UV-absorbing properties and potential biological activities (e.g., antimicrobial effects). In our data, the coumarin was more evident in the ESI- results (listed under phenolic acid derivatives in Table S4, due to its behavior), whereas its signal in ESI+ was weak or not observed, consistent with the compound's ability to ionize as a phenolate anion. While coumarins are not a dominant class in walnut pellicle, their detection expands the palette of phenolic compounds in this tissue and may point to subtle roles in the plant's defense or oxidative chemistry (Thada et al., 2013).

#### 3.2.5 Lignans

A small number of lignans (phenylpropanoid dimers) were tentatively identified in the pellicle extracts. Lignans are not typically abundant in English walnut; however, the pseudotargeted approach allowed us to capture these trace components. For instance, a compound with *m*/*z* 361.2 ([M-H]^−^ in negative mode) was observed, consistent with the molecular weight of secoisolariciresinol or an isomeric lignan. Similarly, we detected features suggestive of other plant lignans (e.g., episyringaresinol 4’-O-β-D-glucopyranoside at m/z 581.2, which typically shows [M-H]^−^ at m/z 581 depending on glycosylation). Due to their low intensity, these lignans were annotated with caution; their MS/MS fragments indicated the presence of the characteristic dihydrobenzofuran structure (for example, fragment ions around m/z 346.1 from secoisolariciresinol after loss of a methyl group). Although present at low levels, lignans broaden the spectrum of pellicle polyphenols. These compounds have noted antioxidant and estrogenic properties in other foods, so even trace amounts in walnut pellicle could contribute to its overall bioactivity profile. The detection of lignans in ESI- (Table S4) also demonstrates the value of using both ionization modes to cover a wide polarity range of metabolites (Vu et al., 2018).

Collectively, polyphenols of diverse structural classes coexist synergistically in walnuts, jointly determining their nutritional value and sensory attributes (Huang et al., 2022). Compared to other nuts, walnuts rank among the highest in total polyphenol content, averaging 1591.5 mg gallic acid equivalents per 100 g of kernels (Pycia et al., 2019). This rich polyphenolic composition underpins the antioxidant and health-promoting effects of walnuts. In summary, a structure-function classification of walnut polyphenols enhances our understanding of their respective contributions to quality and bioactivity, while providing a foundation for comparative analyses across different cultivars or geographical origins.

### 3.3 Unsupervised Modeling for group discrimination

**Fig. 3:**
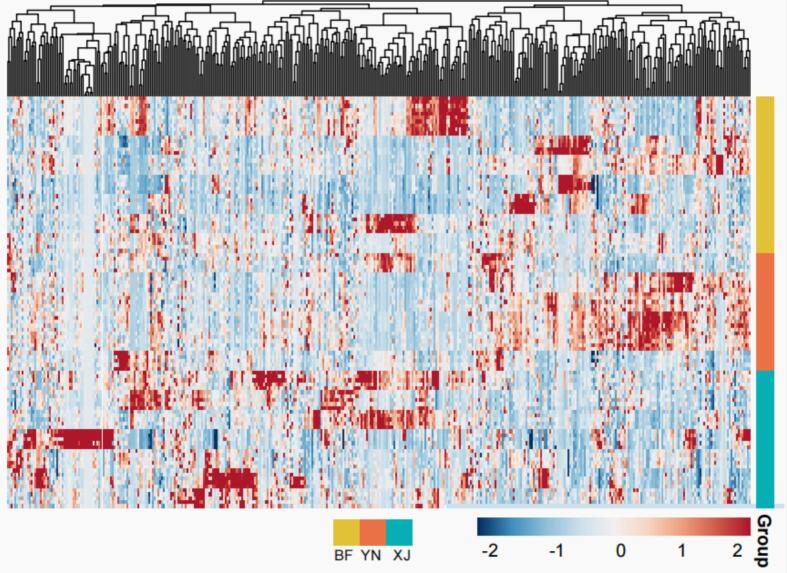
Hierarchical clustering analysis (HCA) heatmap of walnut pellicle samples from different regions. A total of 406 polyphenolic compounds were subjected to hierarchical clustering based on normalized chromatographic peak areas derived from samples collected in three distinct regions. The compounds and sample groups were clustered according to similarity in abundance patterns. In the resulting heatmap, each colour block represents the correlation strength and pattern between a specific polyphenol and its geographical origin, with red indicating high abundance and blue indicating low abundance. (For interpretation of the references to colour in this figure legend, the reader is referred to the web version of this article.)

To explore how geographical origin shapes the metabolome of walnut pellicles, we first applied unsupervised pattern-recognition tools to the complete 406-compound matrix, a strategy widely adopted in nut-authentication studies (Zhu & Xu, 2020). These exploratory analyses provide a global view of variance before any supervised discrimination is attempted, ensuring that subsequent models are grounded in intrinsic data structure (Esteki et al., 2017). The walnut pellicle samples from the three regions showed region-specific grouping in unsupervised multivariate analyses. HCA of the 406 polyphenols produced a heatmap with distinct clusters corresponding to each geographic origin, indicating that samples from different regions shared both common and unique metabolite features (Fig. 3). In the HCA heatmap, metabolites tended to be differentially expressed across subsets of samples, with certain clusters enriched in specific regions (BF, YN, or XJ), suggesting regional variation in pellicle polyphenol composition.

**Fig. 4:**
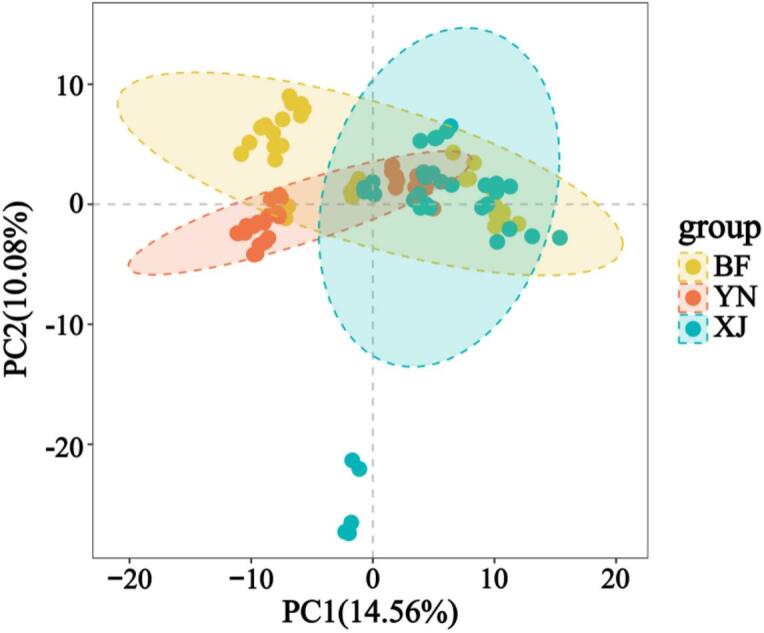
PCA of walnut pellicle samples from three distinct geographic regions. The PCA score plot illustrates the distribution of samples according to the first two principal components, with PC1 explaining 14.56% and PC2 accounting for 10.08% of the total variance.

Consistent trends emerged when the same dataset was projected into principal-component space (Huang et al., 2022). PCA further demonstrated partial separation among the three groups (Fig. 4). In the PCA scores plot (PC1 vs. PC2), samples from regions BF, YN, and XJ showed tendencies to cluster separately, although some overlap was observed among the groups. This partial but discernible separation along the first two principal components indicates moderate inherent differences in metabolite composition between the walnut pellicles of the three origins. The tendency toward group-specific clustering, despite the overlap, suggests that geographical origin may influence the pellicle polyphenol profile, even before supervised analysis. These results imply that walnuts from regions BF, YN, and XJ tend to develop distinguishable polyphenolic fingerprints, likely due to differences in cultivar or environmental factors (climate, soil, agronomic practices) in each region. Together, the HCA and PCA results demonstrate that walnuts from three regions possess differentiable polyphenolic fingerprints—differences most likely driven by cultivar and local environmental factors—and thereby provide a robust foundation for the supervised PLS-DA and OPLS-DA models discussed in the next section.

### 3.4 PLS-DA based group separation

**Fig. 5:**
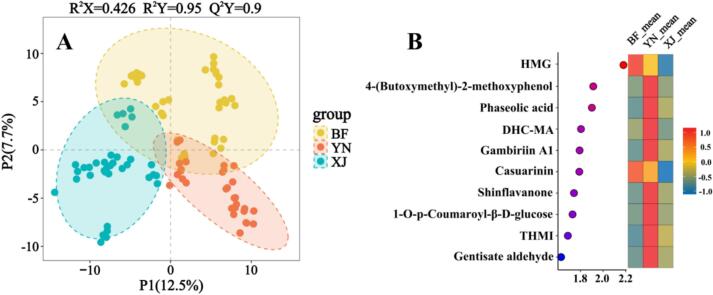
(A) PLS-DA score plot showing metabolic discrimination among walnut pellicle samples from different regions and (B) heatmap and variable importance of selected differential polyphenols across sample groups. Note:6-[4-hydroxy-2-methyl-6-[(2*S*,3*R*,4*S*,5*S*,6*R*)-3,4,5-trihydroxy-6-(hydroxymethyl)oxan-2-yl]oxyphenyl]-4-methoxypyran-2-one: HMG; (2*R*,3*S*)-2-[(3,4-dihydroxyphenyl)methyl]-3-[(E)-3-(3,4-dihydroxyphenyl)prop-2-enoyl]oxy-2-hydroxybutanedioic acid: DHC-MA; (3*R*,4*S*)-4,6,8-Trihydroxy-7-methoxy-3-methyl-3,4-dihydro-1H-isochromen-1-one: THMI.

To more formally discriminate the walnut pellicle samples by origin and identify the metabolites driving these differences, PLS-DA was performed on the entire data set. The PLS-DA score plot showed clear separation among the three groups, indicating strong discriminative power based on their polyphenol profiles. The model yielded cumulative statistics of R^2^X = 0.426, R^2^Y = 0.95, and Q^2^ = 0.90 (Fig. 5A), which exceed commonly accepted thresholds for model stability (R^2^Y > 0.5, Q^2^ > 0.5). These results suggest that the metabolic differences among samples from three regions are systematic and can be effectively captured by supervised multivariate modeling. Permutation testing produced a negative Q^2^-intercept (−1.328), demonstrating that the model is not overfitted. Collectively, these parameters support the use of PLS-DA for exploratory discrimination of walnut pellicles by geographic origin.

To quantitatively evaluate the discriminatory performance of walnut pellicle polyphenols among regions, we further summarized the cross-validated predictions of the three-class PLS-DA model (BF, YN, XJ). Under stratified 7-fold cross-validation repeated 50 times (Table S6), the model achieved an overall accuracy of 0.954 and a balanced accuracy of 0.956 (Table S7). Class-wise recall values were 0.935 for BF, 0.932 for YN, and 0.999 for XJ, while the corresponding precision values were 0.947, 0.979, and 0.942, respectively. One-vs-rest MCC values were 0.905 (BF), 0.937 (YN), and 0.957 (XJ), with a macro-averaged MCC of 0.933 (Table S7). These metrics, together with the negative Q^2^ intercepts observed in 200-label permutation tests, indicate robust but not perfect discrimination and support that the regional separation observed in PCA and PLS-DA is unlikely to be driven by random noise.

Although the high variable-to-sample ratio (406 metabolites measured across 21 samples) may increase the risk of overfitting in supervised models, the combined use of stringent preprocessing, stratified repeated cross-validation, label permutation testing and FDR control was intended to mitigate this risk and provide a conservative assessment of regional discrimination among origins.

Using the PLS-DA model, we examined the VIP scores to pinpoint the metabolites most responsible for differentiating the regions. The VIP scores rank metabolites by their contribution to the group separation, and we focused on the top 10 VIP features as key discriminants. A summary of these top 10 differentiating metabolites and the region in which each showed the highest relative abundance is presented in Fig. 5(B) and Table S5. Several of these discriminant metabolites are known phenolic constituents of walnut pellicle. Casuarinin and gambiriin A1, for example, are ellagitannins (hydrolysable tannins) that contribute to walnut astringency (Huang & Xu, 2021; Mateș et al., 2024). Their higher concentrations in region BF (for casuarinin) and YN (for gambiriin A1) could indicate differences in tannin biosynthesis or polymerization in walnuts grown in those locales. In contrast, region YN pellicles were characterized by elevated amounts of phaseolic acid—also known as trans-caffeoyl-L-malic acid and other hydroxycinnamate derivatives (e.g., p-coumaroyl glucose conjugates). Region XJ exhibited intermediate profiles, but with unique features such as a higher abundance of certain flavonoids (e.g., a flavanone tentatively identified as shinflavanone) and minor phenolic lactones. These patterns are visualized in the heatmap of scaled intensities (Fig. 5B), where each compound's intensity differs by origin, reflecting a distinct polyphenolic fingerprint for each region.

These regional metabolite differences are likely underlain by environmental and genetic factors. Walnut trees synthesize polyphenols as secondary metabolites in response to geoclimatic factors—temperature, humidity, UV radiation, soil composition, etc.—all of which vary by geographic location (Mateș et al., 2024). Region BF's higher tannin content (gambiriin A1, casuarinin) may be linked to a cooler or more arid climate in the Taihang Mountain area (promoting tannin-based defenses), whereas region YN's higher caffeoylmalate (phaseolic acid) could result from different phenylpropanoid pathway flux under the humid, temperate conditions of Yunnan. Notably, year-to-year and site-specific factors can significantly affect walnut phenolic levels (Mateș et al., 2024). Our findings concur that both geography and genotype influence the pellicle's polyphenol composition, yielding region-specific profiles.

Prior research has demonstrated the power of polyphenolic profiling in walnut authentication. Kalogiouri and Samanidou (2021) developed an HPLC-DAD method to quantify walnut phenolics and applied chemometrics to distinguish walnuts from different countries. Their PLS-DA model achieved clear separation of walnuts from Greece, France, and Bulgaria, with markers such as p-coumaric acid, caffeic acid, kaempferol, and myricetin driving the discrimination. This illustrates that a combination of phenolic acids and flavonoids can be diagnostic for geographic origin. In our study, the top features (ellagitannins, caffeoylmalate, etc.) similarly provided a unique pattern for each Chinese region. The ability to fingerprint walnuts by region has practical value for protecting designation of origin and preventing food fraud. High-value walnuts from a reputed origin could be adulterated with cheaper nuts; however, a polyphenol-based authenticity test would detect anomalies in the profile. Moreover, understanding which metabolites are most elevated in a given region can guide quality control—for example, a region known for high ellagitannin content might market its walnuts for their superior antioxidant properties. Overall, the differential polyphenol signatures not only deepen our understanding of walnut phytochemistry but also provide a scientific basis for geographical traceability, leveraging advanced metabolomic profiling to ensure product genuineness and inform breeding or agricultural practices for quality improvement.

### 3.5 OPLS-DA and Differential metabolite analysis

**Fig. 6:**
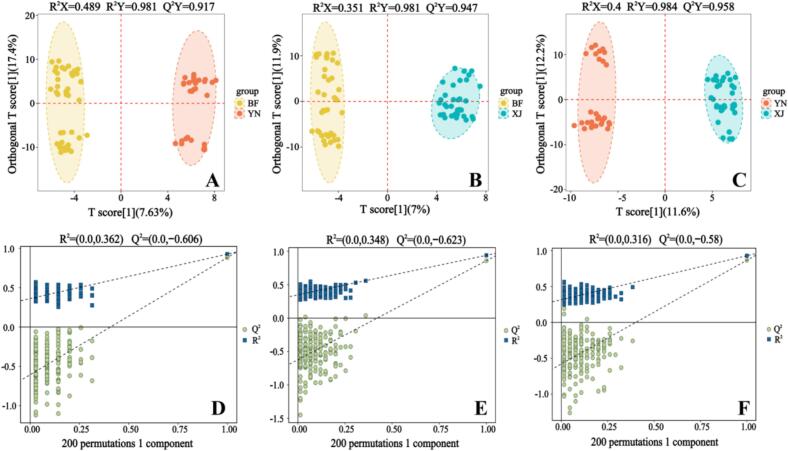
OPLS-DA score plots and permutation test results for pairwise comparisons of walnut pellicle samples from different geographic origins. (A-C) OPLS-DA score plots illustrating group separation among origin BF vs YN (A), BF vs XJ (B), and YN vs XJ (C). (D—F) Corresponding permutation test plots for each model (*n* = 200).

To further investigate the differences between each pair of regions, orthogonal PLS-DA (OPLS-DA) models were constructed for BF vs. YN, BF vs. XJ, and YN vs. XJ (Fig. 6). Each pairwise OPLS-DA model achieved clear separation between the two groups, with strong model performance and validation statistics. The BF vs. YN comparison (one predictive/one orthogonal component) yielded R^2^X (cum) = 0.489, R^2^Y (cum) = 0.981, and Q^2^ (cum) = 0.917; BF vs. XJ gave R^2^X (cum) = 0.351, R^2^Y (cum) = 0.981, and Q^2^ (cum) = 0.947; and YN vs. XJ showed R^2^X (cum) = 0.400, R^2^Y (cum) = 0.984, and Q^2^ (cum) = 0.958. In all three comparisons, the predictive ability (Q^2^ ≈ 0.91–0.96) far exceeded the 0.50 threshold, and permutation testing (200 runs) yielded negative Q^2^-intercepts (−0.623 to −0.580), confirming that the models were not overfitted. Detailed permutation test results and lists of differentially abundant metabolites for each pairwise comparison are provided in Fig. S1. These parameters demonstrate that the metabolomic differences between regions are statistically significant, reproducible, and region-specific.

OPLS-DA loadings revealed a consistent pattern across all three pairwise comparisons (BF vs. YN, BF vs. XJ, and YN vs. XJ), indicating that similar metabolite classes drove the discrimination between walnut pellicle samples from the different origins. Polyphenolic metabolites—principally phenolic acids, flavonoids and hydrolysable ellagitannins—dominated the predictive component in every comparison (Fig. 6). Region BF was characterized by sharply elevated ellagitannin content (notably casuarinin and related tannins), along with a few unusually high phenolic glycosides (e.g., a distinctive glycosylated methoxypyranone). In contrast, region YN was enriched in a broader suite of flavonoids and phenolic acids, exemplified by prunin (naringenin-7-O-β-D-glucoside, a flavanone glycoside) and certain procyanidin-type tannins. Region XJ, on the other hand, lacked the high-level ellagitannins of BF and showed generally lower overall polyphenol levels, with no uniquely dominant markers apart from a modest increase in corilagin (an ellagitannin). These loading trends suggest that origin BF's metabolomic fingerprint is strongly tannin-centered, origin YN combines the greatest diversity and abundance of polyphenols, and origin XJ is comparatively depleted in most of these metabolites. This hierarchy aligns with expected sensory properties: BF's tannin-rich pellicles would impart the most intense bitterness and astringency, YN's diverse but balanced polyphenol profile yields a moderate astringency, and XJ's polyphenol-poor pellicles likely produce the mildest astringency among the three.

To further interpret the separations, several specific high-VIP metabolites were noted as origin-discriminating markers. For instance, casuarinin—a potent antioxidant ellagitannin—was especially elevated in region BF and is known to impart a strongly bitter, drying “bite” to walnut pellicles. Likewise, prunin was putatively identified as a key discriminator for region YN. Prunin is a flavonoid glycoside of naringenin with antioxidant and anti-inflammatory activity and only mild bitterness, which is substantially lower than that of the aglycone naringin (Seong et al., 2023). By contrast, a resveratrol-derived stilbenoid tentatively identified as “ε-viniferin” was characteristically higher in region XJ. The ε-viniferin is recognized for its powerful free-radical scavenging activity (Park et al., 2022) and anti-inflammatory effects (Beaumont et al., 2022). These distinct functional and sensory attributes of the key metabolites support the notion that each region's chemical profile underpins its flavor profile—e.g., tannin-rich compounds like casuarinin drive robust bitterness/astringency in region BF, whereas flavanone glycosides like prunin may impart subtler bitter or citrus notes.

Among the differential metabolites putatively identified by this combined multivariate–univariate framework, casuarinin, prunin, and ε-viniferin were prioritized as geographical marker candidates because they simultaneously exhibited high multivariate importance (VIP > 1.0 in the relevant PLS-DA/OPLS-DA models), statistically significant inter-regional differences after FDR correction (q < 0.05), and large pairwise differences in relative abundance (|log₂FC| > 0.585, i.e., >1.5-fold between the enriched origin and the other two origins), while being robustly detected in all samples (within-origin CV < 30%).

Complementing the multivariate analyses, we performed univariate tests across the 406 metabolites to assess global differences in relative abundance among BF, YN, and XJ. Using one-way ANOVA (or Kruskal-Wallis when normality or variance homogeneity assumptions were violated) followed by Benjamini-Hochberg FDR correction, 214 metabolites exhibited significant regional differences at q < 0.05 (Table S8). In subsequent pairwise comparisons, differential metabolites were defined using log2 fold-changes and FDR-adjusted q-values, with significance thresholds set at |log2FC| > 0.585 and q < 0.05. Candidate geographical marker metabolites were selected by combining these FDR-significant univariate differences (q < 0.05) with high multivariate importance (VIP > 1.0) in the PLS-DA models. The full list of *p*-values, q-values, fold-changes, and VIP scores can be found in Table S8.

Volcano plot-based differential expression analysis was then performed for each pairwise regional comparison (BF vs YN, BF vs XJ, and YN vs XJ) using the same FDR/FC framework. For each contrast, log_2_ fold-changes and -log_10_(q-values) (Benjamini-Hochberg FDR correction across all 406 metabolites) were plotted, and metabolites were considered significantly different if they satisfied |log_2_FC| > 0.585 (corresponding to a > 1.5-fold change) and q < 0.05. Under these FDR/FC thresholds, 104, 102 and 166 metabolites were significantly altered between BF vs YN, BF vs XJ, and YN vs XJ, respectively (Fig. S2). These sets of significant metabolites were then used to construct a three-way Venn diagram (Fig. S3), which summarizes the overlap and uniqueness of differential metabolites across the three pairwise comparisons and highlights both shared polyphenolic responses and region-specific markers.

Importantly, the majority of these discriminants in every pairwise contrast were polyphenols, underscoring that variations in flavonoids, ellagitannins, and related phenolic acids are the primary drivers of the chemical divergence between regions. In the BF-YN comparison, for example, region YN showed higher levels of various phenolics such as hydroxycinnamate conjugates and lignan derivatives (e.g., p-coumaroyl-glucosides and lyoniresinol-type compounds), reflecting YN's broader phenolic spectrum, whereas region BF had greater abundance of certain flavonoids and ellagitannins consistent with its tannin-focused profile (for instance, BF contained more isorhamnetin (a methylated quercetin), catechin/epicatechin derivatives, and ellagitannins like casuarinin). Similarly, the BF-XJ comparison revealed that region XJ was enriched in specific flavonoid subclasses—including polymethoxylated flavones, chalcones, and stilbenoids—which were largely absent or lower in region BF. By contrast, region BF showed significantly higher levels of proanthocyanidins and ellagitannins (e.g. catechin, epicatechin, vescalagin, and casuarinin) relative to XJ, highlighting BF's specialization in tannin-type compounds. In the YN-XJ contrast, region YN contained greater abundances of flavanone glycosides and hydroxycinnamic acid derivatives (for instance, prunin and 1-O-p-coumaroyl-β-d-glucose were elevated in YN), whereas region XJ had relatively higher levels of a few flavonoids that were lower in YN (such as daidzein and certain dihydrochalcones). Across all three comparisons, a clear pattern emerges in which flavonoid glycosides, phenolic acids, and hydrolysable tannins collectively serve as the chemical markers distinguishing the walnut origins. These metabolite differences not only corroborate the multivariate OPLS-DA separations but also provide a concrete chemical basis for the geographic patterning observed. In practical terms, the consistent divergence in key compounds (for example, the dominance of ellagitannins like casuarinin in region BF, the prevalence of flavanone glycosides like prunin in region YN, and the unique stilbenoid presence in region XJ) means that each origin has a reproducible metabolomic “fingerprint.”

Such fingerprints carry both sensory implications and authenticity value: the higher tannin/catechin content of region BF likely underlies its stronger astringent taste, while the lower tannin, flavonoid profile of region XJ corresponds with milder sensory properties, and region YN's balanced polyphenol profile yields intermediate flavor characteristics. Consistently, previous work on walnut pellicle has demonstrated significant positive correlations between tannins/epicatechin and perceived astringency across cultivars (Liu et al., 2021), supporting the notion that the higher tannin/catechin levels in BF samples underlie their stronger astringency relative to XJ and YN. Moreover, these origin-specific metabolite profiles act as robust biomarkers of geographic origin. The fact that compounds like casuarinin, prunin, and ε-viniferin vary systematically and significantly with walnut provenance enables reliable discrimination of origin through their detection. This pseudotargeted metabolomic approach thus provides not only insight into flavor and nutritional differences but also a practical tool for provenance verification in the walnut supply chain. In line with food authenticity studies, the panel of metabolites identified here can be used to authenticate walnuts from different regions, complementing other analytical traceability methods (Schmitt et al., 2020). By leveraging these chemical markers—which are relatively stable traits of a given region's walnuts and less easily altered post-harvest—industry and regulators could verify claimed origins and detect mislabeling of walnut products. In summary, the differential metabolite analysis highlights a comprehensive chemical differentiation among the three walnut origins, linking the metabolome to both sensory attributes and geographic authenticity, and reinforcing how local cultivar and environmental factors (“terroir”) imprint a unique phytochemical signature on the walnut pellicle (Arndt et al., 2020).

Collectively, ellagitannins drive the tannin-centred fingerprint of region BF, a broader suite of flavonoids and phenolic acids underpins the more balanced profile of region YN, whereas region XJ is comparatively depleted in both tannins and flavonoids, with only modest enrichment of a few stilbenoids such as ε-viniferin. Given the strong antioxidant and astringent properties of ellagitannins, these compositional differences rationalise the sensory hierarchy BF > YN > XJ for intensity of bitterness/astringency and provide robust chemical markers for provenance authentication.

### 3.6 KEGG pathway enrichment of differential metabolites by origin

**Fig. 7:**
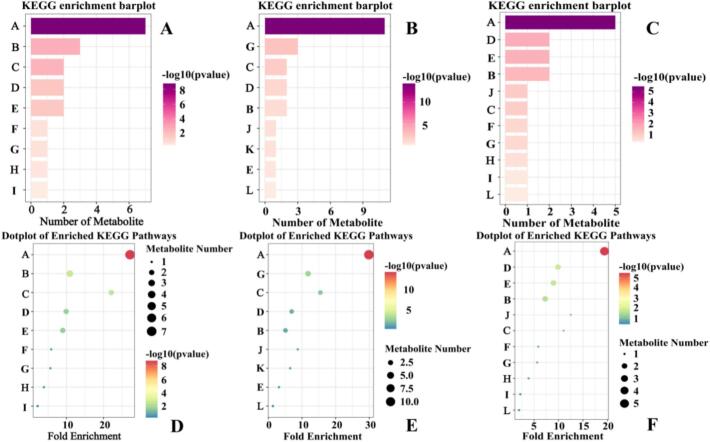
KEGG pathway enrichment analysis of origin-dependent differential metabolites.(Different letters represent different KEGG pathways. A: Flavonoid biosynthesis; B: Tyrosine metabolism; C: Stilbenoid/diarylheptanoid/gingerol biosynthesis; D: Phenylpropanoid biosynthesis; E: Isoflavonoid biosynthesis; F: Phenylalanine metabolism; G: Flavone and flavonol biosynthesis; H: Ubiquinone and other terpenoid-quinone biosynthesis; I: Isoquinoline alkaloid biosynthesis; J: Betalain biosynthesis; K: Glycolysis/Gluconeogenesis; L: Biosynthesis of various plant secondary metabolites.) (A-C) Bar charts for the pairwise comparisons BF vs YN, BF vs XJ, and YN vs XJ, respectively. Bars are ordered by statistical significance [−log_10_(*p*-value)] and coloured on a magenta scale proportional to this value; bar length denotes the number of differential metabolites annotated to each pathway (*p* < 0.05). (D—F) Corresponding dot plots. Each dot represents one KEGG pathway; the x-axis shows the rich factor (ratio of mapped differential metabolites to the total metabolites in that pathway), dot size reflects the number of differential metabolites, and dot colour encodes statistical significance [−log_10_(p-value), green → red]. The dashed vertical line marks the significance threshold (*p* = 0.05; −log₁₀ *P* = 1.3). Pathways plotted toward the upper-right with large, intensely red dots therefore exhibit both strong enrichment and high significance. (For interpretation of the references to colour in this figure legend, the reader is referred to the web version of this article.)

To delineate biochemical distinctions among walnut pellicles from these origins, KEGG pathway enrichment analysis was performed on differential metabolites mapped to compound IDs (BF vs. YN: 14; BF vs. XJ: 20; YN vs. XJ: 18). Enriched pathways (*p* < 0.05) primarily encompassed secondary metabolism, underscoring variations in polyphenol biosynthesis despite shared ellagitannin dominance (Huang et al., 2022). As shown in Fig. 7, Flavonoid biosynthesis (KEGG map00941) was the most prominent across all comparisons, consistent with walnuts' flavonoid and tannin richness, where multiple flavonoid-related compounds drive origin-specific distinctions (Huang et al., 2022; Sheng et al., 2021). These results indicate that multiple flavonoid-related compounds drive the metabolic distinctions between origins. Walnut pellicles are naturally rich in hydrolysable tannins (ellagitannins) and other polyphenols (Huang et al., 2024), so enrichment of flavonoid and phenylpropanoid pathways is unsurprising.

In the BF vs. YN comparison, five pathways were enriched: flavonoid biosynthesis (7/14 metabolites, p.adj = 1.50 × 10^−8^), tyrosine metabolism (map00350), stilbenoid/diarylheptanoid/gingerol biosynthesis (map00945), phenylpropanoid biosynthesis (map00940), and isoflavonoid biosynthesis (map00943). This pathway suite reflects production of diverse flavonoids from phenylpropanoid precursors (Rahim et al., 2023), with variations plausible given walnut pellicles' minor flavonoid content (e.g., catechin, epicatechin, quercetin glycosides) alongside hydrolysable tannins. Tyrosine metabolism (map00350) comprises the catabolic and anabolic routes of the amino acid L-tyrosine, leading to various phenolic and alkaloid compounds. Key differentials included p-coumaric acid (KEGG C00811), derived from tyrosine via tyrosine ammonia-lyase or phenylalanine via phenylalanine ammonia-lyase (PAL), linking tyrosine and phenylpropanoid pathways (Jiang et al., 2019). Stilbenoid enrichment involved compounds like resveratrol (up to ∼1.6 mg/100 g in walnuts), exceeding levels in other nuts (Xu et al., 2024). Isoflavonoid drivers, including daidzein and naringenin (KEGG C00509), indicated a distinct profile for origin YN relative to BF. Notably, naringenin is a pivotal intermediate in flavonoid and isoflavonoid biosynthetic pathways, suggesting higher levels of uncommen polyphenols in YN (Yang & Wang, 2024).

The BF vs. XJ comparison enriched four pathways: flavonoid biosynthesis, flavone and flavonol biosynthesis (map00944), stilbenoid/diarylheptanoid/gingerol biosynthesis, and phenylpropanoid biosynthesis. Recurrent flavonoid and phenylpropanoid enrichment aligned with BF vs. YN, but unique flavone/flavonol specificity suggested pronounced subclass differences (e.g., flavones and flavonols), which is biologically feasible as walnut pellicles contain minor flavonols modulated by environmental or genetic factors (Sheng et al., 2021). With the highest differential count (20 metabolites) and gene ratio for flavonoid biosynthesis (11/20), these results imply substantial metabolomic divergence between BF and XJ, possibly attributable to climatic/soil disparities or varietal differences.

In the YN vs. XJ comparison, four enriched pathways were putatively annotated: flavonoid biosynthesis, phenylpropanoid biosynthesis, isoflavonoid biosynthesis, and tyrosine metabolism. Consistent polyphenol biosynthesis variations emphasized differences in flavan-3-ols, flavonols, and phenolic acids (e.g., tannin or phenolic conjugates) (Ma et al., 2023; Shen et al., 2021). Shared isoflavonoid and tyrosine enrichment mirrored BF vs. YN, with differentials like naringenin, gentisic aldehyde (2,5-dihydroxybenzaldehyde), and 4-hydroxyphenylpyruvate highlighting YN as an outlier in these metabolites (Yang et al., 2024). Absence of stilbenoid or flavone/flavonol enrichment indicated similarity between YN and XJ in those aspects, reinforcing that BF differs from YN/XJ in stilbenoids, XJ from BF in flavonols, and YN uniquely in isoflavonoid/tyrosine-derived compounds (Jahanban-Esfahlan et al., 2019).

Walnut pellicles comprise 1–3% polyphenols by dry weight, predominantly hydrolysable tannins (ellagitannins like pedunculagin, the primary component) and ellagic acid derivatives, with lesser phenolic acids and flavonoids (Regueiro et al., 2014). Genotype or environment may alter pedunculagin concentrations, influencing phenylpropanoid signals (Medic et al., 2021). These bioactive compounds contribute to antioxidant capacity, bitterness/astringency, and health benefits; for example, elevated flavonoids (e.g., flavan-3-ols like catechin or proanthocyanidins) may heighten bitterness but enhance radical scavenging, while higher tannins could extend shelf-life via oxidation protection yet affect processing (e.g., pellicle removal for milder flavor) (Regueiro et al., 2014; Sheng et al., 2021).

Biologically, flavone/flavonol biosynthesis enrichment in origin XJ implies heightened expression of flavonoid hydroxylases or glycosyltransferases, directing metabolites toward flavonol production (e.g., quercetin, kaempferol) as UV protectants in epidermal tissues (Chen et al., 2022). This may stem from stronger sunlight/higher elevation in XJ or inherent genetic variants, yielding flavonol-rich pellicles with distinct antioxidant/anti-inflammatory properties and health/extract applications. Isoflavonoid enrichment in YN represents an atypical signal for non-legumes, potentially from horizontal gene transfer, endophytic microbes, or KEGG annotation overlaps due to structural similarities (e.g., naringenin as a precursor to flavonols or isoflavones) (Yang et al., 2024). Elevated naringenin—a central antioxidant intermediate—may indicate downstream bottlenecks (e.g., enzyme deficiencies), altering end products like proanthocyanidins or ellagitannins, with minor nutritional impacts but revealing genetic polymorphisms or micro-ecosystems. Tyrosine metabolism enrichment in YN underscores diversity in precursors (e.g., gentisate aldehyde, 4-hydroxyphenylpyruvate, p-coumaric acid; KEGG C05585, C00811), tying to phenylpropanoids and suggesting differential phenolic degradation/turnover influenced by storage, microbial load, or enzymes like tyrosine ammonia-lyase/polyphenol oxidase. This could affect pellicle colour (browning via quinones) and flavor (aromatics) (Araji et al., 2014). Stilbenoid/diarylheptanoid differences in BF highlight stress-induced defense metabolites (e.g., resveratrol as phytoalexin against fungi/UV/injury), implying pathogen/climate pressures eliciting stronger responses. Though minor, these confer antioxidant/anticancer benefits, enhancing nutraceutical potential; diarylheptanoids (e.g., curcumin glucoside) may be artifacts or rare microbial contributions.

In summary, pseudotargeted metabolomics effectively classified origins via polyphenol composition, revealing pathway shifts atop ellagitannin foundations: universal flavonoid/phenylpropanoid variations, unique stilbenoid defenses in BF, isoflavonoid-like/tyrosine metabolites in YN, and flavonol concentrations in XJ (Liu et al., 2025). These demonstrate metabolic plasticity under diverse conditions, emphasizing terroir's role in food chemistry and impacts on health (e.g., enhanced antioxidants from flavonols/stilbenes) and sensory qualities (e.g., bitterness from tannins) (Ali & Zeb, 2024). Future investigations should quantify hallmarks (e.g., ellagic acid, pedunculagin, catechin, resveratrol) and correlate with enzyme expression (e.g., PAL, stilbene synthase, flavonoid hydroxylases). Overall, integrating metabolomics with KEGG enrichment yields profound insights into plant food nuances, facilitating walnut origin classification and biochemical diversity comprehension.

Integration of metabolic pathway analysis illuminated linkages between walnut polyphenolic diversity and functional quality. Phenylpropanoid and flavonoid pathways emerged as pivotal determinants of antioxidant content. Flavonoid divergences modulated sensory/health attributes: flavonoids boosted antioxidant/anti-inflammatory potential while imparting subtle bitterness. Region-specific biases thus govern nutraceutical efficacy (e.g., antioxidant strength) and organoleptics via metabolic reprogramming, offering strategies like targeted engineering or breeding to elevate flux in rate-limiting steps for high-polyphenol cultivars without adaptability compromise.

These metabolic variations in polyphenol accumulation stems from intricate genotype-environment interplay, with climate and soil as primary influencers of biosynthesis in plant tissues. Environmental stress can increase oxidative load and thereby induce adaptive upregulation of phenylpropanoid pathways. Consistent with this mechanism, the arid conditions in Xinjiang may promote the accumulation of antioxidant phenolics/tannins through abiotic stimulation (Jiang et al., 2019). Yunnan's subtropical monsoon climate (high precipitation, moderate temperatures) curtails stress-induced metabolism, correlating with lower polyphenol content, though high-altitude UV may partially enhance flavonoids, offset by rainfall/drought absence. Taihang Mountains' temperate climate yields intermediate levels from moderate stimuli (drought/temperature fluctuations/nutrient stress). Soil composition modulates further: nitrogen deficiency shifts carbon to defensive polyphenols, favoring biosynthesis in arid/mountainous, nutrient-poor soils (Xinjiang) over fertile, moist southern ones (Yunnan), potentially influenced by micronutrients. Temperature/growing season length affects allocation: shorter seasons/cooler highs (Xinjiang/Taihang) redirect resources to protective metabolites, while warmer, fast-growing conditions induces dilution, lowering concentrations per dry mass. Precipitation patterns impact disease pressure—humid areas may induce defenses but reduce solar exposure/phenolic buildup. Genetically, *J. regia* (BF/YN) accrues more polyphenols than endemic *J. sigillata* (XJ), indicating biosynthetic adaptations. These interactions can shape the metabolome. Future studies integrating environmental monitoring with controlled experiments (e.g., drought/UV simulations) are needed to elucidate the mechanisms underlying cue-specific responses (Gao et al., 2025; Huang et al., 2022).

## 4 Conclusion

In conclusion, this study represents the first comprehensive application of a pseudotargeted metabolomics approach to profile walnut pellicle polyphenols, resulting in the identification of 406 polyphenolic compounds and revealing clear region-specific chemical fingerprints. The integration of broad-spectrum LC-MS/MS discovery with targeted MRM semi-quantification enabled the capture of both abundant and trace polyphenols, effectively bridging the gap between untargeted and targeted strategies. Each production region exhibited a distinctive metabolomic profile—for example, Taihang Mountain walnuts were enriched in hydrolysable tannins, Yunnan samples contained higher flavonoids and phenolic acids, whereas Xinjiang walnuts showed comparatively lower overall polyphenol content. This methodological innovation underscores the theoretical significance of pseudotargeted metabolomics in food chemistry by providing high-coverage, quantitative insights into complex food matrices. Practically, the distinct polyphenol signatures delineated for each region offer valuable tools for food traceability and quality authentication. The putatively identified region-specific polyphenol biomarkers can serve as chemical tracers to verify the provenance of walnuts and detect mislabeling, thereby safeguarding product genuineness and consumer trust. Additionally, these metabolomic insights could inform targeted breeding and agricultural practices aimed at optimizing polyphenol content for improved nutritional quality. However, we acknowledge that this study's scope was limited to a single harvest period in only three regions, which may not capture seasonal or broader geographic variability in polyphenol profiles. In addition, the putative bioactivities of the identified compounds were not experimentally validated in this work. Future studies should broaden sampling across multiple seasons and additional cultivation areas to verify the consistency of these metabolic fingerprints. Integrating multi-omics approaches would elucidate the genetic and environmental drivers underlying the observed polyphenol variation. Furthermore, correlating the putatively identified metabolite markers with specific health outcomes or functional bioactivities is warranted to confirm their nutritional significance. These efforts will ultimately enhance the utility of pseudotargeted metabolomics in advancing food authenticity, nutritional research, and the valorization of walnut byproducts.

## CRediT authorship contribution statement

**Chang Liu:** Writing – review & editing, Supervision, Project administration, Methodology, Investigation. **Mingxue Geng:** Writing – review & editing, Supervision, Project administration, Methodology, Investigation. **Jiaxin Yin:** Methodology, Investigation. **Huibo Zhao:** Methodology, Investigation. **Bing Qi:** Methodology, Investigation. **Huiqing Li:** Software, Investigation. **Di Wang:** Software, Investigation. **Yanbing Wu:** Validation, Project administration. **Shengxing Dai:** Validation, Project administration. **Min Lu:** Validation, Project administration. **Kuizhang Yao:** Validation, Project administration. **Junxia Xia:** Writing – review & editing, Supervision, Project administration. **Jiankang Deng:** Writing – review & editing, Supervision, Project administration.

## Declaration of competing interest

The authors declare that they have no known competing financial interests or personal relationships that could have appeared to influence the work reported in this paper.

## Data Availability

Data will be made available on request.

## Appendix A Supplementary data

Supplementary material Image 1
