# Supplementary material for: Classification and metabolomic profiling of walnut pellicle polyphenols using a Pseudotargeted metabolomics approach
Source: Food Chem X. 2026 Jan 30;34:103610. doi: 10.1016/j.fochx.2026.103610 (PMC12891871; doi:10.1016/j.fochx.2026.103610)
Supplement: Supplementary file 1 — Supplementary material [file mmc1.docx]

**Supplementary Material**

**Classification and Metabolomic Profiling of Walnut Pellicle Polyphenols Using a Pseudotargeted Metabolomics Approach**

Chang Liu^a,b 1^, Mingxue Geng^b,e 1^ , Jiaxin Yin^b,f^, Huibo Zhao^c,d^, Bing Qi^c,d^ , Huiqing Li^b,e^, Di Wang^c,d^, Yanbing Wu^c,d^, Shengxing Dai^c,d^, Min Lu^c,d^, Kuizhang Yao^c,d^, Junxia Xia^*c,d^ , Jiankang Deng^*^^a,b,d,e^

^a^ School of Life Science and Food Engineering, Huaiyin Institute of Technology, Huai’an 223003, China

^b^ College of Life Science, Hengshui University, Hengshui 053000, China;

^c^ Hebei Yangyuan Zhihui Beverage Co., Ltd. Hengshui 053000, China;

^d^ Hebei Key Laboratory of Walnut Nutritional Function and Processing Technology, Hengshui 053000, China;

^e^ Hebei Technology Innovation Centre of Walnut Beverage, Hengshui 053000, China;

^f^ College of Food Science & Technology, Shanghai Ocean University, Shanghai 201306

***^1^ Contributed equally to this study.***

^*^***Corresponding author*：**

***Junxia Xia****, phone number: +86 03182089919, Email:jishubu_yangyuan@163.com. Address: Hebei Yangyuan Zhihui Beverage Co., Ltd., New Zone 6 Road Fuyang 4 Road, North District of Hebei Hengshui Economic Development Zone , Hengshui053000, China.*

***Jiankang Deng,*** *phone number: +86 03182086119, Email:dengjk1989@163.com. Address: College of Life Science, Hengshui University, No. 1088 Heping West Road, Hengshui, Hebei 053000, China.*

Table S1 Per-sample metadata for the 21 walnut pellicle lots used in this study.

| ID | Region (code) | Production area | Cultivar | Harvest date |
| --- | --- | --- | --- | --- |
| YN-1 | YN (Yunnan) | Yunnan Province, Dali Bai Autonomous Prefecture, Yangbi Yi Autonomous County | Dapao | 2023-9-15 |
| YN-2 | YN (Yunnan) | Yunnan Province, Dali Bai Autonomous Prefecture, Weishan Yi and Hui Autonomous County | Jidanpi | 2023-9-10 |
| YN-3 | YN (Yunnan) | Yunnan Province, Dali Bai Autonomous Prefecture, Yunlong County | Tongzi | 2023-9-10 |
| YN-4 | YN (Yunnan) | Yunnan Province, Dali Bai Autonomous Prefecture, Yangbi Yi Autonomous County | Yuanboluo | 2023-9-15 |
| YN-5 | YN (Yunnan) | Yunnan Province, Chuxiong Yi Autonomous Prefecture, Dayao County | Santai | 2023-9-21 |
| YN-6 | YN (Yunnan) | Yunnan Province, Baoshan City | Xiaomacha | 2023-9-12 |
| BF-1 | BF (Taihang Mountains) | Shanxi Province, Luliang Mountains region | Jinlong-1 | 2023-9-25 |
| BF-2 | BF (Taihang Mountains) | Hebei Province, Shijiazhuang City, Pingshan County | Liaohe-1 | 2023-9-21 |
| BF-3 | BF (Taihang Mountains) | Shanxi Province, Jinzhong City, Zuoquan County | Xiangling | 2023-9-19 |
| BF-4 | BF (Taihang Mountains) | Hebei Province, Xingtai City, Neiqiu County | Lvling | 2023-9-21 |
| BF-5 | BF (Taihang Mountains) | Shanxi Province, Jinzhong City, Zuoquan County | Jinlong-1 | 2023-9-20 |
| BF-6 | BF (Taihang Mountains) | Henan Province, Xinxiang City, Huixian City | Yufeng | 2023-9-19 |
| BF-7 | BF (Taihang Mountains) | Hebei Province, Xingtai City | Qingxiang | 2023-9-19 |
| BF-8 | BF (Taihang Mountains) | Hebei Province, Shijiazhuang City, Zanhuang County | Thin-shell Xiang | 2023-9-23 |
| XJ-1 | XJ (Xinjiang) | Xinjiang Uygur Autonomous Region, Aksu Prefecture | Zaoshi-1 | 2023-9-2 |
| XJ-2 | XJ (Xinjiang) | Xinjiang Uygur Autonomous Region, Changji Hui Autonomous Prefecture, Changji City | Changji-1 | 2023-9-30 |
| XJ-3 | XJ (Xinjiang) | Xinjiang Uygur Autonomous Region, Changji Hui Autonomous Prefecture, Changji City | Changji-2 | 2023-9-30 |
| XJ-4 | XJ (Xinjiang) | Xinjiang Uygur Autonomous Region, Changji Hui Autonomous Prefecture, Jimusaer County | Xinxin-2 | 2023-9-26 |
| XJ-5 | XJ (Xinjiang) | Xinjiang Uygur Autonomous Region, Aksu Prefecture | Wen-185 | 2023-9-5 |
| XJ-6 | XJ (Xinjiang) | Xinjiang Uygur Autonomous Region, Kashgar Prefecture | Xinfeng | 2023-9-2 |
| XJ-7 | XJ (Xinjiang) | Xinjiang Uygur Autonomous Region, Kashgar Prefecture | Zha-343 | 2023-9-10 |

Note: All walnut lots complied with a unified commercial maturity specification: fully mature, commercial-grade in-shell walnuts harvested during the main local harvest season of the sampling year; first-grade (or equivalent) quality with no visible mold, rancidity, insect damage or sprouting. After harvest, walnuts were frozen by the suppliers and transported to the laboratory under frozen conditions. In the laboratory, walnuts were stored at −20 °C until pellicle removal and subsequent polyphenol extraction.

Table S2 ISs used in the pseudotargeted method

| Name | Abbreviation | Volume  (μl) | Concentration  (μg/mL) | ESI+ | | | | ESI- | | | |
| --- | --- | --- | --- | --- | --- | --- | --- | --- | --- | --- | --- |
|  |  |  |  | RT(min) | Precursor  ion (Da) | Product  ion (Da) | CE (V) | RT(min) | Precursor  ion (Da) | Product  ion (Da) | CE (V) |
| Cholic acid-2,2,4,4-d4 | CA-d4 | 5.0 | 0.10 | 11.27 | 395.3 | 359.3 | 15 | 14.05 | 411.3 | 347.2 | -30 |
| Chenodeoxycholic acid-2,2,4,4-d4 | CDCA-d4 | 15.0 | 0.30 | 13.05 | 379.3 | 361.3 | 30 | 15.00 | 395.3 | 377.4 | -30 |
| Decanoyl-L-carnitine-d3 HCl (N-methyl-d3) | Carnitine C10:0-d3 | 7.5 | 0.15 | 10.14 | 319.3 | 85.0 | 30 | - |  |  |  |
| Hexadecanoyl-L-carnitine-d3 HCl (N-methyl-d3) | Carnitine C16:0-d3 | 7.5 | 0.15 | 14.73 | 403.4 | 85.0 | 30 | - |  |  |  |
| Nonadecanoyl-2-hydroxy-sn-glycero-3-phosphocholine | LPC19:0 | 30.0 | 0.60 | 16.76 | 538.4 | 184.1 | 30 | - |  |  |  |
| L-phenylalanine (Ring-d5 ) | Phe-d5 | 30.0 | 0.60 | 1.60 | 171.1 | 124.9 | 15 | 3.26 | 169.0 | 152.1 | -15 |
| L-tryptophan-d5 (Indole-d5) | Trp-d5 | 160.0 | 3.20 | 2.44 | 210.1 | 192.1 | 15 | 4.22 | 208.1 | 120.1 | -15 |

Table S3 Polyphenolic compounds detected and annotated in walnut pellicle extracts via LC-MS/MS (ESI+ mode)

|  | PUBCHEM CID | Compounds | Q1 | Q3 | tR | Databases Level | Score  (Final) | Additional Information | Library Type | Molecular Formula | Adducts | **Main class** |
| --- | --- | --- | --- | --- | --- | --- | --- | --- | --- | --- | --- | --- |
| 1 | 97226 | 10-Hydroxycamptothecin | 365.1 | 203.1 | 6.91 | 2 | 0.69 | ΔMZ=0.00712 Da; 19.5 PPM | KEGG database | C20H16N2O5 | M+H | Other phenolics |
| 2 | 442679 | Eugeniin | 939.1 | 922.1 | 4.77 | 2 | 0.41 | ΔMZ=0.00255 Da; 2.7 PPM | KEGG database | C41H30O26 | M+H | Hydrolysable tannins |
| 3 | 73568 | Corilagin | 652.1 | 453.0 | 9.70 | 3 | 0.48 | ΔMZ=0.00029 Da; 0.4 PPM | Integrated database | C27H22O18 | M+NH4 | Hydrolysable tannins |
| 4 | 131752776 | Methyl 3,4-dihydroxy-5-prenylbenzoate 3-glucoside | 399.2 | 237.1 | 7.36 | 3 | 0.77 | ΔMZ=0.00225 Da; 5.6 PPM | Integrated database | C19H26O9 | M+H | Phenolic acids |
| 5 | 40467697 | Methyl nogalonate | 397.1 | 271.0 | 11.88 | 2 | 0.41 | ΔMZ=0.00169 Da; 4.2 PPM | KEGG database | C21H16O8 | M+H | Phenolic acids |
| 6 | 71668309 | Aklanonate | 397.1 | 235.1 | 7.20 | 2 | 0.93 | ΔMZ=0.00237 Da; 6.0 PPM | KEGG database | C21H16O8 | M+H | Flavonoids |
| 7 | 14034001 | 9-Hydroxy-4-methoxypsoralen 9-glucoside | 395.1 | 233.0 | 7.56 | 3 | 0.88 | ΔMZ=0.00240 Da; 6.1 PPM | Integrated database | C18H18O10 | M+H | Flavonoids / Hydrolysable tannins |
| 8 | 160190 | Aloesin | 395.1 | 202.1 | 6.09 | 2 | 0.38 | ΔMZ=0.00267 Da; 6.8 PPM | KEGG database | C19H22O9 | M+H | Proanthocyanidins |
| 9 | 68077 | Tangeretin | 373.1 | 293.1 | 5.09 | 2 | 0.34 | ΔMZ=0.00031 Da; 0.8 PPM | KEGG database | C20H20O7 | M+H | Flavonoids |
| 10 | 9945785 | 3-p-Coumaroylquinic acid | 361.1 | 303.0 | 6.12 | 3 | 0.85 | ΔMZ=0.00033 Da; 0.9 PPM | Integrated database | C16H18O8 | M+Na | Flavonoids |
| 11 | 5281789 | Licoisoflavone A | 355.1 | 303.0 | 6.14 | 2 | 0.48 | ΔMZ=0.00040 Da; 1.1 PPM | KEGG database | C20H18O6 | M+H | Flavonoids |
| 12 | 441959 | Biflorin | 355.1 | 193.0 | 4.49 | 2 | 0.69 | ΔMZ=0.00042 Da; 1.2 PPM | KEGG database | C16H18O9 | M+H | Flavonoids |
| 13 | 44259848 | Quercetagetin 7-(6''-(E)-caffeoylglucoside) | 643.1 | 178.1 | 7.99 | 3 | 0.51 | ΔMZ=0.00059 Da; 0.9 PPM | Integrated database | C30H26O16 | M+H | Flavonoids |
| 14 | 5281406 | Daphnoretin | 353.1 | 93.0 | 4.85 | 2 | 0.33 | ΔMZ=0.00278 Da; 7.9 PPM | KEGG database | C19H12O7 | M+H | Flavonoids |
| 15 | 11710066 | Parvisoflavone A | 353.1 | 243.1 | 4.82 | 3 | 0.67 | ΔMZ=0.00051 Da; 1.4 PPM | Integrated database | C20H16O6 | M+H | Flavonoids |
| 16 | 53297447 | α-(1,2-Dihydroxyethyl)-1,2,3,4-tetrahydro-7-hydroxy-9-methoxy-3,4-dioxocyclopenta[c][1]benzopyran-6-acetaldehyde | 349.1 | 303.0 | 5.22 | 2 | 1.00 | ΔMZ=0.00279 Da; 8.0 PPM | KEGG database | C17H16O8 | M+H | Flavonoids |
| 17 | 11393902 | Dryopteric acid | 349.1 | 160.1 | 8.75 | 3 | 0.50 | ΔMZ=0.00292 Da; 8.4 PPM | Integrated database | C17H16O8 | M+H | Flavonoids |
| 18 | 135438594 | Miraxanthin-V | 347.1 | 329.1 | 6.96 | 2 | 0.91 | ΔMZ=0.00710 Da; 20.5 PPM | KEGG database | C17H18N2O6 | M+H | Flavonoids |
| 19 | 46173871 | p-Hydroxybenzyldesulphoglucosinolate | 346.1 | 184.0 | 4.54 | 2 | 0.33 | ΔMZ=0.00615 Da; 17.8 PPM | KEGG database | C14H19NO7S | M+H | Flavonoids |
| 20 | 15559735 | Hibiscetin | 335.0 | 303.0 | 4.43 | 3 | 0.45 | ΔMZ=0.00049 Da; 1.5 PPM | Integrated database | C15H10O9 | M+H | Flavonoids |
| 21 | 442009 | Carnosol | 331.2 | 285.2 | 8.33 | 2 | 0.55 | ΔMZ=0.00030 Da; 0.9 PPM | KEGG database | C20H26O4 | M+H | Flavonoids |
| 22 | 442547 | Uncinatone | 327.2 | 263.1 | 8.96 | 2 | 0.47 | ΔMZ=0.00046 Da; 1.4 PPM | KEGG database | C20H22O4 | M+H | Flavonoids |
| 23 | 14057218 | Pterocaryanin B | 635.1 | 303.0 | 2.73 | 3 | 0.44 | ΔMZ=0.00011 Da; 0.2 PPM | Integrated database | C27H22O18 | M+H | Flavonoids |
| 24 | 440835 | Leucodelphidin | 323.1 | 277.0 | 5.48 | 2 | 0.86 | ΔMZ=0.00269 Da; 8.3 PPM | KEGG database | C15H14O8 | M+H | Flavonoids |
| 25 |  | 2-(3,4,5-Trihydroxyphenyl)-3,4-dihydro-2H-1-benzopyran-3,4,5,7-tetrol | 323.1 | 162.1 | 6.98 | 3 | 0.50 | ΔMZ=0.00266 Da; 8.2 PPM | Integrated database | C15H14O8 | M+H | Flavonoids |
| 26 | 73588 | Taxodione | 315.2 | 121.1 | 21.38 | 2 | 0.59 | ΔMZ=0.00270 Da; 8.6 PPM | KEGG database | C20H26O3 | M+H | Flavonoids |
| 27 | 148556 | 3-O-Acetylpinobanksin | 315.1 | 195.0 | 8.06 | 3 | 0.69 | ΔMZ=0.00834 Da; 26.5 PPM | Integrated database | C17H14O6 | M+H | Flavonoids |
| 28 | 439503 | Salicin | 309.1 | 147.0 | 7.97 | 2 | 0.69 | ΔMZ=0.00191 Da; 6.2 PPM | KEGG database | C13H18O7 | M+Na | Flavonoids |
| 29 | 441960 | Cimifugin | 307.1 | 231.0 | 7.03 | 2 | 0.66 | ΔMZ=0.00285 Da; 9.3 PPM | KEGG database | C16H18O6 | M+H | Flavonoids |
| 30 | 72277 | Epigallo catechin | 307.1 | 149.0 | 16.28 | 1 | 0.31 | ΔMZ=0.00512 Da; 16.7 PPM | Standards database | C15H14O7 | M+H | Flavonoids |
| 31 | 439533 | Taxifolin | 305.1 | 287.1 | 9.53 | 2 | 0.91 | ΔMZ=0.00007 Da; 0.2 PPM | KEGG database | C15H12O7 | M+H | Flavonoids |
| 32 | 5281855 | Ellagic acid | 303.0 | 286.0 | 6.13 | 1 | 0.91 | ΔMZ=0.00002 Da; 0.1 PPM | Standards database | C14H6O8 | M+H | Flavonoids |
| 33 | 5280343 | Quercetin | 303.0 | 286.0 | 8.62 | 1 | 1.00 | ΔMZ=0.00026 Da; 0.9 PPM | Standards database | C15H10O7 | M+H | Flavonoids |
| 34 | 21579130 | 2-[3,4-bis[[(2S,3R,4S,5S,6R)-3,4,5-Trihydroxy-6-(hydroxymethyl)oxan-2-yl]oxy]phenyl]-5,7-dihydroxychromen-4-one | 628.2 | 429.1 | 23.87 | 3 | 0.62 | ΔMZ=0.00743 Da; 11.8 PPM | Integrated database | C27H30O16 | M+NH4 | Flavonoids |
| 35 | 5281642 | 6-Hydroxyluteolin | 303.0 | 285.0 | 8.62 | 2 | 0.78 | ΔMZ=0.00032 Da; 1.1 PPM | KEGG database | C15H10O7 | M+H | Flavonoids |
| 36 | 440735 | Eriodictyol | 289.1 | 231.1 | 11.95 | 2 | 0.71 | ΔMZ=0.00030 Da; 1.1 PPM | KEGG database | C15H12O6 | M+H | Flavonoids |
| 37 | 122850 | Dihydrokaempferol | 289.1 | 224.9 | 9.48 | 1 | 0.40 | ΔMZ=0.00028 Da; 1.0 PPM | Standards database | C15H12O6 | M+H | Flavonoids |
| 38 | 4788 | Phloretin | 275.1 | 169.0 | 9.47 | 1 | 0.96 | ΔMZ=0.00026 Da; 1.0 PPM | Standards database | C15H14O5 | M+H | Flavonoids |
| 39 | 439246 | Naringenin | 273.1 | 189.1 | 6.23 | 1 | 0.77 | ΔMZ=0.00031 Da; 1.1 PPM | Standards database | C15H12O5 | M+H | Flavonoids |
| 40 | 23724670 | 6,7,4'-Trihydroxyflavanone | 273.1 | 172.0 | 7.60 | 2 | 0.35 | ΔMZ=0.00034 Da; 1.3 PPM | KEGG database | C15H12O5 | M+H | Flavonoids |
| 41 | 822798 | Pyrogallin | 205.0 | 177.1 | 9.22 | 3 | 0.80 | ΔMZ=0.00242 Da; 11.8 PPM | Integrated database | C11H8O4 | M+H | Simple phenols |
| 42 | 5280567 | 4-Methyumbelliferone | 177.1 | 159.0 | 7.57 | 1 | 0.68 | ΔMZ=0.00025 Da; 1.4 PPM | Standards database | C10H8O3 | M+H | Coumarins |
| 43 | 5316590 | Gallomyricitrin | 617.1 | 304.1 | 7.26 | 3 | 0.67 | ΔMZ=0.00017 Da; 0.3 PPM | Integrated database | C28H24O16 | M+H | Flavonoids |
| 44 | 5480249 | [(2S,3R,4S,5S,6R)-2-[2-(3,4-Dihydroxyphenyl)-5,7-dihydroxy-4-oxo-chromen-3-yl]oxy-4,5-dihydroxy-6-(hydroxymethyl)tetrahydropyran-3-yl] 3,4,5-trihydroxybenzoate | 617.1 | 304.1 | 6.02 | 3 | 0.86 | ΔMZ=0.00039 Da; 0.6 PPM | Integrated database | C28H24O16 | M+H | Flavonoids |
| 45 | 85270241 | Castacrenin A | 615.1 | 597.1 | 6.02 | 3 | 0.47 | ΔMZ=0.00008 Da; 0.1 PPM | Integrated database | C27H18O17 | M+H | Phenolic acids |
| 46 | 85262147 | Castacrenin B | 615.1 | 597.0 | 6.70 | 3 | 0.49 | ΔMZ=0.00026 Da; 0.4 PPM | Integrated database | C27H18O17 | M+H | Phenolic acids |
| 47 | 10438425 | Osmanthuside B | 593.2 | 153.0 | 7.63 | 3 | 0.95 | ΔMZ=0.00249 Da; 4.2 PPM | Integrated database | C29H36O13 | M+H | Hydrolysable tannins |
| 48 |  | 3-{(E)-2-[4-(β-D-Glucopyranosyloxy)-2-hydroxyphenyl]vinyl}-5-hydroxyphenyl β-D-glucopyranoside | 591.2 | 307.1 | 15.61 | 3 | 1.00 | ΔMZ=0.00006 Da; 0.1 PPM | Integrated database | C26H32O14 | M+Na | Flavonoids / Hydrolysable tannins |
| 49 | 73644 | Casuarictin | 937.1 | 919.1 | 4.76 | 2 | 0.47 | ΔMZ=0.00138 Da; 1.5 PPM | KEGG database | C41H28O26 | M+H | Flavonoids / Phenolic acids |
| 50 | [44259256](https://pubchem.ncbi.nlm.nih.gov/compound/44259256) | Quercetin 3-(2''-galloyl-α-L-arabinopyranoside) | 587.1 | 417.1 | 7.60 | 3 | 0.89 | ΔMZ=0.00009 Da; 0.2 PPM | Integrated database | C27H22O15 | M+H | Lignans |
| 51 | 91666354 | Quercetin 3-(6''-p-hydroxybenzoylgalactoside) | 585.1 | 304.1 | 20.87 | 3 | 0.42 | ΔMZ=0.00011 Da; 0.2 PPM | Integrated database | C28H24O14 | M+H | Proanthocyanidins |
| 52 | 45482321 | [Episyringaresinol 4'-O-β-D-glucopyranoside](https://www.ncbi.nlm.nih.gov/pcsubstance/?term=) | 581.2 | 523.2 | 8.48 | 2 | 0.75 | ΔMZ=0.00213 Da; 3.7 PPM | KEGG database | C28H36O13 | M+H | Proanthocyanidins |
| 53 | 14284599 | Epicatechin-(4β->8)-gallocatechin | 579.2 | 409.1 | 4.10 | 2 | 0.71 | ΔMZ=0.00036 Da; 0.6 PPM | KEGG database | C30H26O12 | M+H | Proanthocyanidins |
| 54 | 147299 | Procyanidin B4 | 579.1 | 409.1 | 7.00 | 2 | 0.35 | ΔMZ=0.00053 Da; 0.9 PPM | KEGG database | C30H26O12 | M+H | Proanthocyanidins |
| 55 | 124017 | Procyanidin B5 | 579.1 | 409.1 | 5.16 | 2 | 0.38 | ΔMZ=0.00011 Da; 0.2 PPM | KEGG database | C30H26O12 | M+H | Phenolic terpenoids |
| 56 | 122738 | Procyanidin B2 | 579.1 | 287.1 | 4.18 | 2 | 0.88 | ΔMZ=0.00043 Da; 0.7 PPM | KEGG database | C30H26O12 | M+H | Flavonoids |
| 57 | 101421537 | Mulberrofuran S | 577.2 | 425.1 | 7.19 | 3 | 0.81 | ΔMZ=0.00376 Da; 6.5 PPM | Integrated database | C34H24O9 | M+H | Flavonoids |
| 58 | 42607565 | Okanin 4'-(3'',4'',6''-triacetylglucoside) | 577.2 | 293.1 | 6.03 | 3 | 0.53 | ΔMZ=0.00246 Da; 4.3 PPM | Integrated database | C27H28O14 | M+H | Hydrolysable tannins |
| 59 | 42607564 | Okanin 4'-(2'',4'',6''-triacetylglucoside) | 577.2 | 287.1 | 7.40 | 3 | 0.54 | ΔMZ=0.00231 Da; 4.0 PPM | Integrated database | C27H28O14 | M+H | Stilbenoids |
| 60 | 13834145 | Casuarinin | 937.1 | 345.0 | 4.78 | 2 | 0.42 | ΔMZ=0.00055 Da; 0.6 PPM | KEGG database | C41H28O26 | M+H | Hydrolysable tannins |
| 61 | 57518718 | ε-Viniferin | 575.2 | 413.1 | 9.92 | 3 | 0.67 | ΔMZ=0.00369 Da; 6.4 PPM | Integrated database | C35H26O8 | M+H | Stilbenoids |
| 62 | 10793018 | 5,7-Dihydroxy-3',4'-dimethoxy-8-(3-hydroxy-3-methylbutyl)-isoflavone 7-glucoside | 563.2 | 401.2 | 5.17 | 3 | 0.52 | ΔMZ=0.00286 Da; 5.1 PPM | Integrated database | C28H34O12 | M+H | Flavonoids |
| 63 | 21630004 | (S)-(4-Hydroxy-3-methoxyphenyl)[(3S,4R,5S)-5-(4-hydroxy-3-methoxyphenyl)-4-(hydroxymethyl)tetrahydro-3-furanyl]methyl β-D-glucopyranoside | 561.2 | 303.0 | 10.02 | 3 | 0.53 | ΔMZ=0.00069 Da; 1.2 PPM | Integrated database | C26H34O12 | M+Na | Lignans |
| 64 | 44257094 | Epicatechin 5-O-β-D-glucopyranoside-3-benzoate | 557.2 | 303.0 | 8.55 | 3 | 0.94 | ΔMZ=0.00230 Da; 4.1 PPM | Integrated database | C28H28O12 | M+H | Flavonoids / Phenolic acids |
| 65 |  | Cyclopenta[c]pyran-4-carboxylic acid, 1,4a,5,7a-tetrahydro-7-(hydroxymethyl)-1-[[6-O-[(2E)-3-(4-hydroxyphenyl)-1-oxo-2-propen-1-yl]-β-D-glucopyranosyl]oxy]-, methyl ester | 557.2 | 153.0 | 18.20 | 3 | 1.00 | ΔMZ=0.00023 Da; 0.4 PPM | Integrated database | C26H30O12 | M+Na | Phenolic aldehydes/ketones |
| 66 | 46173978 | Lappaol C | 555.2 | 349.1 | 7.98 | 2 | 0.58 | ΔMZ=0.00246 Da; 4.4 PPM | KEGG database | C30H34O10 | M+H | Lignans |
| 67 |  | 3-(4-{[1,3-Dihydroxy-1-(4-hydroxy-3-methoxyphenyl)-2-propanyl]oxy}-3-methoxyphenyl)propyl 6-deoxy-α-L-mannopyranoside | 547.2 | 385.2 | 7.99 | 3 | 0.81 | ΔMZ=0.00010 Da; 0.2 PPM | Integrated database | C26H36O11 | M+Na | Phenolic acids |
| 68 | 10625849 | [(2R,3R,4S)-6-Hydroxy-4-(4-hydroxy-3-methoxyphenyl)-3-(hydroxymethyl)-7-methoxy-1,2,3,4-tetrahydro-2-naphthalenyl]methyl β-D-glucopyranoside | 545.2 | 512.5 | 8.58 | 3 | 0.98 | ΔMZ=0.00005 Da; 0.1 PPM | Integrated database | C26H34O11 | M+Na | Coumarins |
| 69 | 11526601 | Curcumin monoglucoside | 531.2 | 514.2 | 11.48 | 2 | 0.79 | ΔMZ=0.00282 Da; 5.3 PPM | KEGG database | C27H30O11 | M+H | Diarylheptanoids |
| 70 | 45782997 | 3-Ethenyl-4-[2-(3,4,5-trihydroxybenzoyl)oxyethyl]-2-[3,4,5-trihydroxy-6-(hydroxymethyl)oxan-2-yl]oxy-3,4-dihydro-2H-pyran-5-carboxylic acid | 529.2 | 511.0 | 7.88 | 3 | 1.00 | ΔMZ=0.00381 Da; 7.2 PPM | Integrated database | C23H28O14 | M+H | Phenolic acids |
| 71 | 168165 | Vescalagin | 935.1 | 881.0 | 6.02 | 3 | 0.41 | ΔMZ=0.00001 Da; 0.0 PPM | Integrated database | C41H26O26 | M+H | Hydrolysable tannins |
| 72 | 44260064 | 5,4'-Dihydroxy-6,7,8,3'-tetramethoxyflavone 4'-galactoside | 523.1 | 395.0 | 13.60 | 3 | 0.62 | ΔMZ=0.00193 Da; 3.7 PPM | Integrated database | C24H26O13 | M+H | Flavonoids |
| 73 | 5281777 | Iridin | 523.1 | 362.1 | 10.35 | 2 | 0.72 | ΔMZ=0.00204 Da; 3.9 PPM | KEGG database | C24H26O13 | M+H | Lignans |
| 74 | 5281780 | Isochlorogenic acid b | 517.1 | 391.2 | 7.50 | 2 | 0.53 | ΔMZ=0.00198 Da; 3.8 PPM | KEGG database | C25H24O12 | M+H | Phenolic acids |
| 75 | 23872115 | [4a-Hydroxy-7-(hydroxymethyl)-1-[3,4,5-trihydroxy-6-(hydroxymethyl)oxan-2-yl]oxy-5,7a-dihydro-1H-cyclopenta[c]pyran-5-yl] 4-hydroxybenzoate | 505.1 | 335.1 | 5.53 | 3 | 0.94 | ΔMZ=0.00019 Da; 0.4 PPM | Integrated database | C22H26O12 | M+Na | Phenolic acids |
| 76 | 44257846 | Apigenin 7-(6''-crotonylglucoside) | 501.1 | 153.0 | 7.18 | 3 | 0.98 | ΔMZ=0.00219 Da; 4.4 PPM | Integrated database | C25H24O11 | M+H | Flavonoids |
| 77 | 45359160 | 3-(1-Acetoxy-3-methylbutyl)-6-{2-hydroxy-4-methyl-6-[(1E)-3-oxo-1-buten-1-yl]phenoxy}-2-methoxybenzoic acid | 493.2 | 419.2 | 11.45 | 3 | 0.89 | ΔMZ=0.00589 Da; 12.0 PPM | Integrated database | C26H30O8 | M+Na | Flavonoids |
| 78 | 44258500 | 6-Hydroxyluteolin 6,7-dimethyl ether 4'-glucoside | 491.2 | 474.2 | 7.38 | 3 | 0.89 | ΔMZ=0.00883 Da; 18.0 PPM | Integrated database | C24H26O11 | M+H | Flavonoids |
| 79 | 74978029 | Kaempferol 3-(6-acetylgalactoside) | 491.1 | 178.1 | 8.18 | 3 | 0.70 | ΔMZ=0.00023 Da; 0.5 PPM | Integrated database | C23H22O12 | M+H | Flavonoids |
| 80 | 51136399 | 5-Hydroxy-3-[4-hydroxy-2-[(2S,3R,4S,5S,6R)-3,4,5-trihydroxy-6-(hydroxymethyl)oxan-2-yl]oxyphenyl]-7-methoxychromen-4-one | 485.1 | 85.1 | 19.67 | 3 | 0.68 | ΔMZ=0.00722 Da; 14.9 PPM | Integrated database | C22H22O11 | M+Na | Flavonoids |
| 81 | 440221 | 1-O,6-O-Digalloyl-β-D-glucose | 485.1 | 303.0 | 4.91 | 2 | 0.93 | ΔMZ=0.00038 Da; 0.8 PPM | KEGG database | C20H20O14 | M+H | Hydrolysable tannins |
| 82 | 124022 | 3,5-Di-O-galloyl-4-O-digalloylquinic acid | 801.1 | 335.0 | 4.52 | 2 | 0.37 | ΔMZ=0.00061 Da; 0.8 PPM | KEGG database | C35H28O22 | M+H | Hydrolysable tannins |
| 83 | 93039 | Catalposide | 483.1 | 277.0 | 9.91 | 2 | 0.81 | ΔMZ=0.00224 Da; 4.6 PPM | KEGG database | C22H26O12 | M+H | Phenolic acids |
| 84 | 44257804 | Apigenin 7-(6''-ethylglucuronide) | 475.1 | 429.2 | 6.48 | 3 | 0.86 | ΔMZ=0.00246 Da; 5.2 PPM | Integrated database | C23H22O11 | M+H | Flavonoids |
| 85 | 14861229 | 4''-O-Acetylafzelin | 475.1 | 303.0 | 6.64 | 3 | 0.66 | ΔMZ=0.00039 Da; 0.8 PPM | Integrated database | C23H22O11 | M+H | Flavonoids |
| 86 | 44258953 | Kaempferol 3-(2''-acetylrhamnoside) | 475.1 | 303.0 | 6.78 | 3 | 0.66 | ΔMZ=0.00262 Da; 5.5 PPM | Integrated database | C23H22O11 | M+H | Flavonoids |
| 87 | [25799413](https://pubchem.ncbi.nlm.nih.gov/compound/25799413) | 1-[2-(β-D-Glucopyranosyloxy)-4,6-dihydroxyphenyl]-3-(4-methoxyphenyl)-1-propanone | 473.1 | 427.2 | 8.72 | 3 | 0.73 | ΔMZ=0.00029 Da; 0.6 PPM | Integrated database | C22H26O10 | M+Na | Phenolic aldehydes/ketones |
| 88 | 38362596 | (2S,3S,4S,5R,6S)-3,4,5-Trihydroxy-6-[5-hydroxy-6-methoxy-2-(4-methoxyphenyl)-4-oxochromen-7-yl]oxyoxane-2-carboxylic acid | 473.1 | 391.0 | 10.65 | 3 | 0.90 | ΔMZ=0.00299 Da; 6.3 PPM | Integrated database | C23H22O12 | M+H-H2O | Flavonoids |
| 89 |  | 2-(β-D-Glucopyranosyloxy)benzyl (2E)-3-(3,4-dihydroxyphenyl)acrylate | 471.1 | 453.0 | 10.16 | 3 | 0.57 | ΔMZ=0.00013 Da; 0.3 PPM | Integrated database | C22H24O10 | M+Na | Phenolic acids |
| 90 | 5280459 | Quercitrin | 471.1 | 339.0 | 14.40 | 1 | 0.53 | ΔMZ=0.00032 Da; 0.7 PPM | Standards database | C21H20O11 | M+H | Flavonoids |
| 91 | 44257156 | Catechin-4-ol 3'-methyl ether 3-O-α-L-rhamnopyranoside | 467.2 | 305.1 | 11.04 | 3 | 0.97 | ΔMZ=0.00206 Da; 4.4 PPM | Integrated database | C22H26O11 | M+H | Flavonoids |
| 92 | 14035439 | Castanin | 785.1 | 465.1 | 3.25 | 3 | 0.58 | ΔMZ=0.00036 Da; 0.5 PPM | Integrated database | C34H24O22 | M+H | Hydrolysable tannins |
| 93 | 125142 | Isomucronulator 7-O-glucoside | 465.2 | 304.1 | 6.35 | 3 | 0.69 | ΔMZ=0.00227 Da; 4.9 PPM | Integrated database | C23H28O10 | M+H | Flavonoids |
| 94 | 42607774 | Bracteatin 6-O-glucoside | 465.1 | 448.1 | 4.37 | 2 | 0.35 | ΔMZ=0.00027 Da; 0.6 PPM | KEGG database | C21H20O12 | M+H | Flavonoids |
| 95 | 25203368 | Quercetin-3-glucoside | 465.1 | 304.1 | 6.35 | 2 | 0.76 | ΔMZ=0.00032 Da; 0.7 PPM | KEGG database | C21H20O12 | M+H | Flavonoids |
| 96 | 5281673 | Myricitrin | 465.1 | 303.1 | 6.29 | 1 | 0.31 | ΔMZ=0.00015 Da; 0.3 PPM | Standards database | C21H20O12 | M+H | Flavonoids |
| 97 | 51136585 | 2-Hydroxycyclohexyl 2-O-[(2E)-3-(3,4-Dihydroxyphenyl)-2-propenoyl]-β-D-glucopyranoside | 463.2 | 395.0 | 6.87 | 3 | 0.65 | ΔMZ=0.00016 Da; 0.4 PPM | Integrated database | C21H28O10 | M+Na | Phenolic acids |
| 98 | 13607752 | Luteolin-7-O-glucuronide | 463.1 | 445.1 | 8.46 | 2 | 0.45 | ΔMZ=0.00244 Da; 5.3 PPM | KEGG database | C21H18O12 | M+H | Flavonoids |
| 99 | 5281810 | Tectoridin | 463.1 | 324.1 | 4.75 | 2 | 0.63 | ΔMZ=0.00236 Da; 5.1 PPM | KEGG database | C22H22O11 | M+H | Flavonoids |
| 100 | 5320735 | [3-Hydroxy-4-[(2S,3R,4S,5S,6R)-3,4,5-trihydroxy-6-(hydroxymethyl)oxan-2-yl]oxyphenyl]methyl 3,4-dihydroxybenzoate | 461.1 | 153.0 | 16.11 | 3 | 0.97 | ΔMZ=0.00210 Da; 4.5 PPM | Integrated database | C20H22O11 | M+Na | Phenolic aldehydes/ketones |
| 101 |  | 5-Hydroxy pseudo baptigenin 7-O-glucoside | 461.1 | 153.0 | 7.29 | 3 | 0.73 | ΔMZ=0.00007 Da; 0.2 PPM | Integrated database | C22H20O11 | M+H | Flavonoids |
| 102 | 6072 | Phlorizin | 459.1 | 412.2 | 4.98 | 2 | 0.57 | ΔMZ=0.00023 Da; 0.5 PPM | KEGG database | C21H24O10 | M+Na | Flavonoids |
| 103 | 44259470 | Myricetin 3-(2'',3''-digalloylrhamnoside) | 769.1 | 335.0 | 12.50 | 3 | 0.46 | ΔMZ=0.00161 Da; 2.1 PPM | Integrated database | C35H28O20 | M+H | Flavonoids / Hydrolysable tannins |
| 104 | 53398699 | 6''-O-Acetyldaidzin | 459.1 | 177.1 | 9.63 | 3 | 0.54 | ΔMZ=0.00042 Da; 0.9 PPM | Integrated database | C23H22O10 | M+H | Flavonoids |
| 105 | 44257119 | Epigallocatechin 3-O-vanillate | 457.1 | 153.0 | 7.82 | 3 | 1.00 | ΔMZ=0.00281 Da; 6.1 PPM | Integrated database | C23H20O10 | M+H | Flavonoids / Phenolic acids |
| 106 | 5281714 | Gnetin A | 455.2 | 425.1 | 7.85 | 2 | 0.94 | ΔMZ=0.00354 Da; 7.8 PPM | KEGG database | C28H22O6 | M+H | Stilbenoids |
| 107 | 44257155 | Catechin-4-ol 3-O-α-L-rhamnopyranoside | 453.1 | 407.0 | 9.97 | 3 | 0.78 | ΔMZ=0.00245 Da; 5.4 PPM | Integrated database | C21H24O11 | M+H | Flavonoids |
| 108 | 442260 | Auriculoside | 451.2 | 393.2 | 13.92 | 2 | 0.88 | ΔMZ=0.00184 Da; 4.1 PPM | KEGG database | C22H26O10 | M+H | Flavonoids |
| 109 | 54699185 | Lonchocarpenin | 449.2 | 317.2 | 12.00 | 2 | 0.47 | ΔMZ=0.00299 Da; 6.6 PPM | KEGG database | C27H28O6 | M+H | Flavonoids |
| 110 | 6449879 | (2R,3S)-2-[(3,4-Dihydroxyphenyl)methyl]-2-hydroxy-3-[(E)-3-(4-hydroxy-3-methoxyphenyl)prop-2-enoyl]oxybutanedioic acid | 449.1 | 318.0 | 6.79 | 3 | 0.63 | ΔMZ=0.00011 Da; 0.3 PPM | Integrated database | C21H20O11 | M+H | Flavonoids |
| 111 | 5276454 | Catechin 3-O-gallate | 443.1 | 331.1 | 7.01 | 3 | 0.53 | ΔMZ=0.00220 Da; 5.0 PPM | Integrated database | C22H18O10 | M+H | Flavonoids / Hydrolysable tannins |
| 112 | 14463111 | Robinetinidol 3-O-gallate | 443.1 | 291.1 | 6.40 | 3 | 0.92 | ΔMZ=0.00033 Da; 0.7 PPM | Integrated database | C22H18O10 | M+H | Flavonoids / Hydrolysable tannins |
| 113 | 6419835 | (-)-Catechin 3-O-gallate | 443.1 | 153.0 | 5.76 | 3 | 0.58 | ΔMZ=0.00258 Da; 5.8 PPM | Integrated database | C22H18O10 | M+H | Flavonoids / Hydrolysable tannins |
| 114 | 44258000 | Quercetin 3-(2'',6''-digalloylgalactoside) | 769.1 | 303.0 | 9.80 | 3 | 0.51 | ΔMZ=0.00087 Da; 1.1 PPM | Integrated database | C35H28O20 | M+H | Flavonoids / Hydrolysable tannins |
| 115 | 44260021 | Flaccidine | 443.1 | 153.0 | 5.76 | 3 | 0.50 | ΔMZ=0.00268 Da; 6.0 PPM | Integrated database | C23H22O9 | M+H | Flavonoids |
| 116 | 10478277 | sec-o-Glucosylhamaudol | 439.2 | 307.1 | 8.72 | 2 | 0.72 | ΔMZ=0.00280 Da; 6.4 PPM | KEGG database | C21H26O10 | M+H | Flavonoids |
| 117 | 21722046 | Orotinichalcone | 437.2 | 303.1 | 19.67 | 3 | 0.93 | ΔMZ=0.00270 Da; 6.2 PPM | Integrated database | C26H28O6 | M+H | Flavonoids |
| 118 | 46882581 | Melledonol | 435.2 | 417.1 | 6.11 | 3 | 0.62 | ΔMZ=0.00262 Da; 6.0 PPM | Integrated database | C23H30O8 | M+H | Flavonoids |
| 119 | 5320861 | Quercetin-3-β-D-xyloside | 435.1 | 418.1 | 5.65 | 3 | 0.63 | ΔMZ=0.00031 Da; 0.7 PPM | Integrated database | C20H18O11 | M+H | Flavonoids |
| 120 | 23724768 | 8-C-Glucosylnaringenin | 435.1 | 392.1 | 8.24 | 2 | 0.80 | ΔMZ=0.00249 Da; 5.7 PPM | KEGG database | C21H22O10 | M+H | Flavonoids |
| 121 | 44260086 | 5,2'-Dihydroxy-3,6,7,8,4',5'-hexamethoxyflavone | 435.1 | 391.1 | 8.23 | 3 | 0.45 | ΔMZ=0.00218 Da; 5.0 PPM | Integrated database | C21H22O10 | M+H | Flavonoids |
| 122 | 101789466 | Eriodictin | 435.1 | 390.0 | 8.25 | 3 | 0.52 | ΔMZ=0.00043 Da; 1.0 PPM | Integrated database | C21H22O10 | M+H | Flavonoids |
| 123 | 5490064 | Avicularin | 435.1 | 304.0 | 9.76 | 3 | 0.48 | ΔMZ=0.00014 Da; 0.3 PPM | Integrated database | C20H18O11 | M+H | Flavonoids |
| 124 | 92794 | Naringenin 7-O-β-D-glucoside | 435.1 | 303.0 | 5.77 | 2 | 0.80 | ΔMZ=0.00024 Da; 0.6 PPM | KEGG database | C21H22O10 | M+H | Flavonoids |
| 125 | 6440594 | Occidentoside | 705.2 | 543.1 | 8.91 | 3 | 0.99 | ΔMZ=0.00309 Da; 4.4 PPM | Integrated database | C36H32O15 | M+H | Flavonoids |
| 126 | 5318659 | Phlorizin chalcone | 435.1 | 303.0 | 6.60 | 2 | 0.39 | ΔMZ=0.00267 Da; 6.1 PPM | KEGG database | C21H22O10 | M+H | Flavonoids |
| 127 | 5280507 | Sinapyl alcohol | 421.2 | 191.1 | 7.31 | 1 | 0.59 | ΔMZ=0.00033 Da; 0.8 PPM | Standards database | C11H14O4 | 2M+H | Phenolic acids |
| 128 | 5487077 | 5,7,2'-Trihydroxy-3,6,8,4',5'-pentamethoxyflavone | 421.1 | 303.0 | 2.51 | 3 | 0.50 | ΔMZ=0.00024 Da; 0.6 PPM | Integrated database | C20H20O10 | M+H | Flavonoids |
| 129 | 637213 | Rhaponticin | 421.1 | 114.1 | 7.10 | 2 | 0.97 | ΔMZ=0.00235 Da; 5.6 PPM | KEGG database | C21H24O9 | M+H | Stilbenoids |
| 130 | 162305 | 6-[4-Hydroxy-2-methyl-6-[(2S,3R,4S,5S,6R)-3,4,5-trihydroxy-6-(hydroxymethyl)oxan-2-yl]oxyphenyl]-4-methoxypyran-2-one | 411.1 | 383.0 | 4.46 | 3 | 1.00 | ΔMZ=0.00264 Da; 6.4 PPM | Integrated database | C19H22O10 | M+H | Flavonoids |
| 131 | 5495929 | 9-Hydroxycalabaxanthone | 409.2 | 353.1 | 14.30 | 3 | 1.00 | ΔMZ=0.00212 Da; 5.2 PPM | Integrated database | C24H24O6 | M+H | Xanthones |
| 132 | 11143989 | Garcimangosone B | 409.2 | 353.1 | 13.03 | 3 | 1.00 | ΔMZ=0.00198 Da; 4.8 PPM | Integrated database | C24H24O6 | M+H | Xanthones |
| 133 | 5459223 | 4'-O-Prenylalpinumisoflavone | 405.2 | 387.0 | 7.22 | 3 | 0.90 | ΔMZ=0.00339 Da; 8.4 PPM | Integrated database | C25H24O5 | M+H | Flavonoids |
| 134 | 95168 | Osajin | 405.2 | 153.0 | 10.79 | 2 | 0.79 | ΔMZ=0.00319 Da; 7.9 PPM | KEGG database | C25H24O5 | M+H | Flavonoids |
| 135 | 442744 | Frangulin B | 403.1 | 385.1 | 8.96 | 2 | 0.96 | ΔMZ=0.00007 Da; 0.2 PPM | KEGG database | C20H18O9 | M+H | Quinones |

ID: Unique identifier for the compound. PUBCHEM CID: PubChem Compound ID for structural reference. Compounds: Name of the annotated metabolite (e.g., catechin, gallic acid). Q1: Precursor ion (m/z) used for quantification (Quantifier ion in MRM). Q3: Product ion (*m/z*) used for qualification (Qualifier ion in MRM). tR: Retention time (minutes) in the LC-MS method. Parameter settings used in MRM-Ion Pair Finder: Δm/z (MS1) = 0.0100 Da; Δm/z (MS2) = 0.0500 Da; MS² scoring method = forward; reference noise (unknown/standard MS²) = 1.0/200.0; minimum neighboring fragments to merge a peak cluster = 2; maximum fragments investigated = 20.

Table S4 Polyphenolic compounds detected and annotated in walnut pellicle extracts via LC-MS/MS (ESI-mode)

|  | PUBCHEM CID | Compounds | Q1 | Q3 | tR | Databases Level | Score  (Final) | Additional Information | Library Type | Molecular Formula | Adducts | Main class |
| --- | --- | --- | --- | --- | --- | --- | --- | --- | --- | --- | --- | --- |
| 1 | 70949 | Gentisate aldehyde | 137.0 | 93.0 | 4.49 | 1 | 0.86 | ΔMZ=0.00132 Da; 9.7 PPM | Standards database | C7H6O3 | M-H | Phenolic aldehydes/ketones |
| 2 | 338 | Salicylic acid | 137.0 | 93.0 | 3.05 | 1 | 0.86 | ΔMZ=0.00169 Da; 12.3 PPM | Standards database | C7H6O3 | M-H | Phenolic acids |
| 3 | 121805 | 2-Methoxyresorcinol | 139.0 | 124.0 | 2.68 | 3 | 0.89 | ΔMZ=0.00083 Da; 5.9 PPM | Integrated database | C7H8O3 | M-H | Simple phenols |
| 4 | 19 | 2,3-Dihydroxybenzoic Acid | 153.0 | 109.0 | 3.97 | 1 | 0.79 | ΔMZ=0.00028 Da; 1.9 PPM | Standards database | C7H6O4 | M-H | Phenolic acids |
| 5 | 1491 | 2,4-Dihydroxybenzoic Acid | 153.0 | 123.0 | 1.47 | 1 | 0.85 | ΔMZ=0.00215 Da; 14.0 PPM | Standards database | C7H6O4 | M-H | Phenolic acids |
| 6 | 637542 | trans-p-Hydroxycinnamic acid | 163.0 | 147.0 | 7.60 | 3 | 0.55 | ΔMZ=0.00866 Da; 53.1 PPM | Integrated database | C9H8O3 | M-H | Phenolic acids |
| 7 | 547 | 3,4-Dihydroxyphenylacetic acid | 167.0 | 123.0 | 3.55 | 3 | 0.45 | ΔMZ=0.00036 Da; 2.2 PPM | Integrated database | C8H8O4 | M-H | Phenolic acids |
| 8 | 11874 | 2,3,4-Trihydroxybenzoic acid | 169.0 | 126.0 | 1.77 | 3 | 0.93 | ΔMZ=0.00066 Da; 3.9 PPM | Integrated database | C7H6O5 | M-H | Phenolic acids |
| 9 | 66520 | Phloroglucinol carboxylic acid | 169.0 | 126.0 | 1.76 | 1 | 0.46 | ΔMZ=0.00178 Da; 10.6 PPM | Standards database | C7H6O5 | M-H | Phenolic acids |
| 10 | 5280567 | 4-Methylumbelliferone | 176.0 | 173.0 | 4.11 | 2 | 0.37 | ΔMZ=0.00769 Da; 43.7 PPM | KEGG database | C10H8O3 | M-H | Coumarins |
| 11 | 5281416 | Esculetin | 177.0 | 133.0 | 5.32 | 1 | 0.76 | ΔMZ=0.00146 Da; 8.2 PPM | Standards database | C9H6O4 | M-H | Coumarins |
| 12 | 5280536 | 4-Hydroxy-3-methoxycinnamaldehyde | 177.1 | 175.0 | 7.96 | 1 | 0.50 | ΔMZ=0.00211 Da; 11.9 PPM | Standards database | C10H10O3 | M-H | Phenolic aldehydes/ketones |
| 13 | 636708 | 2-Hydroxy-3-(4-hydroxyphenyl)propenoate | 179.0 | 151.0 | 8.19 | 2 | 0.37 | ΔMZ=0.00056 Da; 3.1 PPM | KEGG database | C9H8O4 | M-H | Phenolic acids |
| 14 | 6443769 | 3-(3,5-Dihydroxyphenyl)-2-propenoic acid | 179.0 | 164.0 | 9.31 | 3 | 0.43 | ΔMZ=0.00050 Da; 2.8 PPM | Integrated database | C9H8O4 | M-H | Phenolic acids |
| 15 | 689043 | Caffeic acid | 179.0 | 135.0 | 4.10 | 1 | 0.75 | ΔMZ=0.00239 Da; 13.4 PPM | Standards database | C9H8O4 | M-H | Phenolic acids |
| 16 | 5282146 | trans-2,3-Dihydroxycinnamate | 179.0 | 136.0 | 2.05 | 2 | 0.86 | ΔMZ=0.00054 Da; 3.0 PPM | KEGG database | C9H8O4 | M-H | Phenolic acids |
| 17 | 24135 | 1-(2,6-Dihydroxy-4-methoxyphenyl)ethanone | 181.1 | 167.0 | 14.58 | 1 | 0.61 | ΔMZ=0.00128 Da; 7.1 PPM | Standards database | C9H10O4 | M-H | Phenolic aldehydes/ketones |
| 18 | 8655 | 4-Hydroxy-3,5-dimethoxybenzaldehyde | 181.1 | 153.0 | 4.89 | 3 | 0.58 | ΔMZ=0.00003 Da; 0.2 PPM | Integrated database | C9H10O4 | M-H | Phenolic aldehydes/ketones |
| 19 | 78016 | 4-O-Methylgallic acid | 183.0 | 168.0 | 4.84 | 3 | 0.91 | ΔMZ=0.00055 Da; 3.0 PPM | Integrated database | C8H8O5 | M-H | Phenolic acids |
| 20 | 76600 | Methyl 2,4,6-trihydroxybenzoate | 183.0 | 168.0 | 8.39 | 3 | 0.88 | ΔMZ=0.00056 Da; 3.0 PPM | Integrated database | C8H8O5 | M-H | Phenolic acids |
| 21 | 7428 | Methyl gallate | 183.0 | 168.0 | 6.22 | 1 | 0.67 | ΔMZ=0.00143 Da; 7.8 PPM | Standards database | C8H8O5 | M-H | Phenolic acids |
| 22 | 169539 | (-)-trans-3,4-Dihydro-4,8-dihydroxy-3-methyl-1H-2-benzopyran-1-one | 193.0 | 175.0 | 5.01 | 3 | 0.98 | ΔMZ=0.00063 Da; 3.3 PPM | Integrated database | C10H10O4 | M-H | Coumarins |
| 23 | 348315909 | 2,5-Dihydroxycinnamic acid methyl ester | 193.1 | 175.0 | 10.31 | 3 | 0.87 | ΔMZ=0.00050 Da; 2.6 PPM | Integrated database | C10H10O4 | M-H | Phenolic acids |
| 24 | 709 | 3-(4-Hydroxy-3-methoxy-phenyl)prop-2-enoic acid | 193.1 | 178.0 | 10.33 | 3 | 0.83 | ΔMZ=0.00030 Da; 1.6 PPM | Integrated database | C10H10O4 | M-H | Phenolic acids |
| 25 | 66654 | Xanthoxylin | 195.1 | 136.1 | 6.08 | 2 | 0.53 | ΔMZ=0.00059 Da; 3.0 PPM | KEGG database | C10H12O4 | M-H | Coumarins |
| 26 | 21677748 | 3-(3,4-Dihydroxyphenyl)lactate | 197.0 | 182.0 | 8.97 | 2 | 0.44 | ΔMZ=0.00052 Da; 2.6 PPM | KEGG database | C9H10O5 | M-H | Phenolic acids |
| 27 | 1245 | Dl-4-Hydroxy-3-methoxymandelic acid | 197.0 | 182.0 | 7.44 | 1 | 0.58 | ΔMZ=0.00165 Da; 8.4 PPM | Standards database | C9H10O5 | M-H | Phenolic acids |
| 28 | 16547 | 2, 4-Diacetylphloroglucinol | 209.0 | 165.1 | 9.98 | 1 | 0.70 | ΔMZ=0.00002 Da; 0.1 PPM | Standards database | C10H10O5 | M-H | Simple phenols |
| 29 | 5084146 | 4-(Butoxymethyl)-2-methoxyphenol | 209.1 | 121.0 | 2.02 | 3 | 0.79 | ΔMZ=0.00042 Da; 2.0 PPM | Integrated database | C12H18O3 | M-H | Simple phenols |
| 30 | 3083616 | 8-Hydroxy-6,7-dimethoxy-2H-chromen-2-one | 221.0 | 189.0 | 4.29 | 3 | 0.92 | ΔMZ=0.00037 Da; 1.7 PPM | Integrated database | C11H10O5 | M-H | Coumarins |
| 31 | 8512 | 1-Hydroxyanthraquinone | 223.0 | 205.0 | 0.84 | 1 | 0.30 | ΔMZ=0.00513 Da; 23.0 PPM | Standards database | C14H8O3 | M-H | Quinones |
| 32 | 9920917 | Dehydrochorismic acid | 223.0 | 123.0 | 14.00 | 3 | 1.00 | ΔMZ=0.00739 Da; 33.1 PPM | Integrated database | C10H8O6 | M-H | Phenolic acids |
| 33 | 637775 | Sinapic acid | 223.1 | 195.0 | 8.85 | 1 | 0.36 | ΔMZ=0.00388 Da; 17.4 PPM | Standards database | C11H12O5 | M-H | Phenolic acids |
| 34 | 445154 | Resveratrol | 228.1 | 184.1 | 9.19 | 2 | 0.63 | ΔMZ=0.00898 Da; 39.4 PPM | KEGG database | C14H12O3 | M-H | Stilbenoids |
| 35 | 591742 | (S)-2,3-Dihydro-7-hydroxy-2-methyl-4-oxo-4H-1-benzopyran-5-acetic acid | 235.1 | 191.1 | 9.03 | 3 | 0.57 | ΔMZ=0.00040 Da; 1.7 PPM | Integrated database | C12H12O5 | M-H | Coumarins |
| 36 | 5381458 | Radicinin | 235.1 | 220.0 | 6.58 | 3 | 0.66 | ΔMZ=0.00029 Da; 1.2 PPM | Integrated database | C12H12O5 | M-H | Other phenolics |
| 37 | 25774975 | (3R,4S)-4,6,8-Trihydroxy-7-methoxy-3-methyl-3,4-dihydro-1H-isochromen-1-one | 239.1 | 211.0 | 5.16 | 3 | 0.58 | ΔMZ=0.00010 Da; 0.4 PPM | Integrated database | C11H12O6 | M-H | Coumarins |
| 38 | 45359356 | 4,6-Dihydroxy-3-(1-hydroxyethyl)-5-methoxy-3H-2-benzofuran-1-one | 239.1 | 179.0 | 6.39 | 3 | 0.88 | ΔMZ=0.00027 Da; 1.1 PPM | Integrated database | C11H12O6 | M-H | Coumarins |
| 39 | 20980930 | Galloylglycerol | 243.1 | 169.0 | 3.57 | 3 | 0.81 | ΔMZ=0.00022 Da; 0.9 PPM | Integrated database | C10H12O7 | M-H | Hydrolysable tannins |
| 40 | 667639 | Piceatannol | 243.1 | 179.0 | 9.70 | 2 | 0.68 | ΔMZ=0.00781 Da; 32.1 PPM | KEGG database | C14H12O4 | M-H | Stilbenoids |
| 41 | 5281254 | Hispidol | 253.1 | 195.1 | 0.77 | 2 | 0.46 | ΔMZ=0.00580 Da; 22.9 PPM | KEGG database | C15H10O4 | M-H | Flavonoids |
| 42 | 6683 | Purpurin | 255.0 | 213.1 | 11.06 | 2 | 0.74 | ΔMZ=0.00006 Da; 0.2 PPM | KEGG database | C14H8O5 | M-H | Quinones |
| 43 | 638278 | Isoliquiritigenin | 255.1 | 213.1 | 11.07 | 1 | 0.59 | ΔMZ=0.00031 Da; 1.2 PPM | Standards database | C15H12O4 | M-H | Flavonoids |
| 44 | 5319688 | 2'-O-Methylisoliquiritigenin | 269.1 | 179.1 | 0.77 | 2 | 0.34 | ΔMZ=0.00602 Da; 22.4 PPM | KEGG database | C16H14O4 | M-H | Flavonoids |
| 45 | 161871 | Fukiic acid | 271.0 | 211.0 | 2.58 | 3 | 0.76 | ΔMZ=0.00033 Da; 1.2 PPM | Integrated database | C11H12O8 | M-H | Phenolic acids |
| 46 | 440936 | Arbutin | 271.1 | 161.0 | 2.01 | 1 | 0.93 | ΔMZ=0.00110 Da; 4.0 PPM | Standards database | C12H16O7 | M-H | Simple phenols |
| 47 | 9838356 | Dihydrogenistein | 271.1 | 211.0 | 9.50 | 2 | 0.69 | ΔMZ=0.00001 Da; 0.1 PPM | KEGG database | C15H12O5 | M-H | Flavonoids |
| 48 | 442410 | Garbanzol | 271.1 | 211.0 | 9.63 | 2 | 0.85 | ΔMZ=0.00005 Da; 0.2 PPM | KEGG database | C15H12O5 | M-H | Flavonoids |
| 49 | 439246 | Naringenin | 271.1 | 203.0 | 14.63 | 1 | 0.79 | ΔMZ=0.00041 Da; 1.5 PPM | Standards database | C15H12O5 | M-H | Flavonoids |
| 50 | 42607905 | Naringerin | 271.1 | 177.0 | 8.97 | 1 | 0.90 | ΔMZ=0.00439 Da; 16.2 PPM | Standards database | C15H12O5 | M-H | Flavonoids |
| 51 | 24891369 | N-[4'-hydroxy-(E)-cinnamoyl]-L-aspartic acid | 278.1 | 216.1 | 3.16 | 3 | 0.78 | ΔMZ=0.00002 Da; 0.1 PPM | Integrated database | C13H13NO6 | M-H | Phenolic acids |
| 52 | 131751430 | Feruloyl-2-hydroxyputrescine | 279.1 | 161.0 | 6.58 | 3 | 0.93 | ΔMZ=0.00984 Da; 35.2 PPM | Integrated database | C14H20N2O4 | M-H | Phenolic acids |
| 53 | 131752962 | 2-O-Caffeoyltartronic acid | 281.0 | 237.0 | 0.68 | 3 | 0.89 | ΔMZ=0.00009 Da; 0.3 PPM | Integrated database | C12H10O8 | M-H | Phenolic acids |
| 54 | 131750880 | 3'-Methoxyfukiic acid | 285.1 | 153.0 | 3.63 | 3 | 0.86 | ΔMZ=0.00033 Da; 1.1 PPM | Integrated database | C12H14O8 | M-H | Phenolic acids |
| 55 | 132594 | Uralenneoside | 285.1 | 165.0 | 3.80 | 3 | 0.52 | ΔMZ=0.00004 Da; 0.1 PPM | Integrated database | C12H14O8 | M-H | Hydrolysable tannins |
| 56 | 181994 | (+-)-Dalbergioidin | 287.1 | 269.0 | 11.12 | 2 | 0.63 | ΔMZ=0.00018 Da; 0.6 PPM | KEGG database | C15H12O6 | M-H | Flavonoids |
| 57 | 21932272 | 2,4',5,7-Tetrahydroxyflavanone | 287.1 | 243.1 | 10.07 | 3 | 0.64 | ΔMZ=0.00024 Da; 0.8 PPM | Integrated database | C15H12O6 | M-H | Flavonoids |
| 58 | 122850 | Dihydrokaempferol | 287.1 | 205.0 | 3.68 | 1 | 0.74 | ΔMZ=0.00022 Da; 0.8 PPM | Standards database | C15H12O6 | M-H | Flavonoids |
| 59 | 182232 | (+)-Epicatechin | 289.0 | 245.0 | 4.73 | 1 | 0.97 | ΔMZ=0.00063 Da; 2.2 PPM | Standards database | C15H14O6 | M-H | Flavonoids |
| 60 | 72276 | (-)-Epicatechin | 289.1 | 271.1 | 4.88 | 3 | 0.80 | ΔMZ=0.00096 Da; 3.3 PPM | Integrated database | C15H14O6 | M-H | Flavonoids |
| 61 | 9064 | (+)-Catechin | 289.1 | 245.1 | 4.87 | 1 | 0.86 | ΔMZ=0.00064 Da; 2.2 PPM | Standards database | C15H14O6 | M-H | Flavonoids |
| 62 | 5281786 | Phaseolic acid | 295.0 | 251.1 | 4.22 | 2 | 0.89 | ΔMZ=0.00020 Da; 0.7 PPM | KEGG database | C13H12O8 | M-H | Phenolic acids |
| 63 | 5281779 | Irilone | 297.0 | 279.0 | 14.34 | 2 | 0.95 | ΔMZ=0.00001 Da; 0.0 PPM | KEGG database | C16H10O6 | M-H | Flavonoids |
| 64 | 15413154 | 7-Hydroxy-5-methoxy-6,8-dimethylflavanone | 297.1 | 179.1 | 3.75 | 3 | 0.47 | ΔMZ=0.00575 Da; 19.4 PPM | Integrated database | C18H18O4 | M-H | Flavonoids |
| 65 | 123917 | Enterolactone | 297.1 | 179.1 | 1.18 | 2 | 0.62 | ΔMZ=0.00608 Da; 20.5 PPM | KEGG database | C18H18O4 | M-H | Lignans |
| 66 | 159278 | Salidroside | 299.1 | 239.1 | 3.94 | 2 | 0.81 | ΔMZ=0.00002 Da; 0.1 PPM | KEGG database | C14H20O7 | M-H | Phenolic acids |
| 67 | 5281642 | 6-Hydroxyluteolin | 301.0 | 169.0 | 3.26 | 2 | 0.95 | ΔMZ=0.00983 Da; 32.7 PPM | KEGG database | C15H10O7 | M-H | Flavonoids |
| 68 | 128853 | Delphinidin | 301.0 | 273.0 | 6.01 | 1 | 0.39 | ΔMZ=0.00261 Da; 8.7 PPM | Standards database | C15H10O7 | M-H | Flavonoids |
| 69 | 442757 | Nanafrocin | 301.1 | 205.0 | 23.18 | 2 | 0.32 | ΔMZ=0.00003 Da; 0.1 PPM | KEGG database | C16H14O6 | M-H | Quinones |
| 70 | 5320471 | Viscidulin I | 301.1 | 286.0 | 8.03 | 3 | 0.77 | ΔMZ=0.00823 Da; 27.3 PPM | Integrated database | C15H10O7 | M-H | Flavonoids |
| 71 | 471 | (+/-)-Taxifolin | 304.1 | 286.0 | 6.55 | 3 | 0.73 | ΔMZ=0.00395 Da; 13.0 PPM | Integrated database | C15H12O7 | M-H | Flavonoids |
| 72 | 54675870 | 2-Protocatechoylphloroglucinolcarboxylate | 305.0 | 291.0 | 10.11 | 2 | 0.68 | ΔMZ=0.00000 Da; 0.0 PPM | KEGG database | C14H10O8 | M-H | Phenolic acids |
| 73 | 9882981 | (-)-Gallocatechin | 305.1 | 219.1 | 7.30 | 1 | 0.74 | ΔMZ=0.00117 Da; 3.8 PPM | Standards database | C15H14O7 | M-H | Flavonoids |
| 74 | 5281724 | 4'-Prenyloxyresveratrol | 311.1 | 205.1 | 3.16 | 2 | 0.97 | ΔMZ=0.00012 Da; 0.4 PPM | KEGG database | C19H20O4 | M-H | Stilbenoids |
| 75 | 5317284 | 6-Methoxyluteolin | 315.1 | 313.1 | 12.68 | 3 | 0.44 | ΔMZ=0.00846 Da; 26.9 PPM | Integrated database | C16H12O7 | M-H | Flavonoids |
| 76 | 14235076 | Eriodictyol 7,3'-dimethyl ether | 315.1 | 184.0 | 6.63 | 3 | 0.93 | ΔMZ=0.00661 Da; 21.0 PPM | Integrated database | C17H16O6 | M-H | Flavonoids |
| 77 | 5281691 | Rhamnetin | 315.1 | 300.0 | 7.02 | 2 | 0.53 | ΔMZ=0.00034 Da; 1.1 PPM | KEGG database | C16H12O7 | M-H | Flavonoids |
| 78 | 18406287 | Isopulegone caffeate | 315.2 | 233.0 | 11.01 | 3 | 0.86 | ΔMZ=0.00296 Da; 9.4 PPM | Integrated database | C19H24O4 | M-H | Phenolic acids |
| 79 | 5281654 | Isorhamnetin | 316.1 | 241.0 | 6.16 | 2 | 0.48 | ΔMZ=0.00703 Da; 22.2 PPM | KEGG database | C16H12O7 | M-H | Flavonoids |
| 80 | 56658060 | Dihydroisorhamnetin | 317.1 | 301.0 | 7.03 | 3 | 0.42 | ΔMZ=0.00003 Da; 0.1 PPM | Integrated database | C16H14O7 | M-H | Flavonoids |
| 81 | 5319108 | 3,4,8,9,10-Pentahydroxy-6-oxo-6H-benzo[c]chromene-1-[carboxylate](https://www.ncbi.nlm.nih.gov/pcsubstance/?term=) | 319.0 | 275.0 | 8.18 | 3 | 0.89 | ΔMZ=0.00015 Da; 0.5 PPM | Integrated database | C14H8O9 | M-H | Coumarins |
| 82 | 1038132 | 2,3-Dihydro-2,3-dihydroxy-4-(4-methoxyphenyl)-1H-phenalen-1-one | 319.1 | 301.1 | 0.69 | 3 | 0.53 | ΔMZ=0.00390 Da; 12.2 PPM | Integrated database | C20H16O4 | M-H | Flavonoids |
| 83 | 102210469 | 3-p-Coumaroyl-1,5-quinolactone | 319.1 | 173.0 | 7.37 | 3 | 0.65 | ΔMZ=0.00004 Da; 0.1 PPM | Integrated database | C16H16O7 | M-H | Phenolic acids |
| 84 | 341 | 3,4-Dihydroxy-5-(3,4,5-trihydroxybenzoyloxy)benzoic acid；  Digallic acid | 321.0 | 170.0 | 3.18 | 3 | 0.73 | ΔMZ=0.00013 Da; 0.4 PPM | Integrated database | C14H10O9 | M-H | Hydrolysable tannins |
| 85 | 85362234 | 4-(3,4-Dihydroxyphenyl)-2,3-dihydro-2,3-dihydroxy-1H-phenalen-1-one | 321.1 | 303.1 | 0.85 | 3 | 0.48 | ΔMZ=0.00588 Da; 18.3 PPM | Integrated database | C19H14O5 | M-H | Phenolic acids |
| 86 | 124052 | Glabridin | 323.1 | 169.0 | 4.65 | 2 | 0.95 | ΔMZ=0.00599 Da; 18.5 PPM | KEGG database | C20H20O4 | M-H | Flavonoids |
| 87 | 14158116 | 1-O-p-Coumaroyl-β-D-glucose | 325.1 | 235.1 | 4.35 | 3 | 0.98 | ΔMZ=0.00020 Da; 0.6 PPM | Integrated database | C15H18O8 | M-H | Phenolic acids |
| 88 | 10471692 | 3-(4-Hydroxyphenyl)-3-oxopropyl-β-D-glucopyranoside | 327.1 | 290.1 | 8.58 | 3 | 0.40 | ΔMZ=0.00017 Da; 0.5 PPM | Integrated database | C15H20O8 | M-H | Phenolic acids |
| 89 | 45783012 | 4-Acetyl-3-hydroxy-5-methylphenyl β-D-glucopyranoside | 327.1 | 211.0 | 8.56 | 3 | 0.90 | ΔMZ=0.00030 Da; 0.9 PPM | Integrated database | C15H20O8 | M-H | Simple phenols |
| 90 | 66065 | Bergenin | 327.1 | 207.0 | 2.42 | 2 | 0.39 | ΔMZ=0.00014 Da; 0.4 PPM | KEGG database | C14H16O9 | M-H | Hydrolysable tannins |
| 91 | 131751404 | 1,3,5-Trihydroxy-6,7-dimethoxy-2-methylanthraquinone | 329.1 | 253.1 | 9.92 | 3 | 0.83 | ΔMZ=0.00765 Da; 23.2 PPM | Integrated database | C17H14O7 | M-H | Quinones |
| 92 | 85366474 | 3-(3,4-Dihydroxyphenyl)-1-propanol 3'-glucoside | 329.1 | 149.1 | 9.01 | 3 | 0.73 | ΔMZ=0.00008 Da; 0.2 PPM | Integrated database | C15H22O8 | M-H | Phenolic acids |
| 93 | 85305084 | 3'-Glucosyl-2',4',6'-trihydroxyacetophenone | 329.1 | 269.1 | 10.62 | 3 | 0.81 | ΔMZ=0.00014 Da; 0.4 PPM | Integrated database | C14H18O9 | M-H | Phenolic aldehydes/ketones |
| 94 | 3083845 | Demethoxysudachitin | 329.1 | 313.1 | 7.89 | 3 | 0.41 | ΔMZ=0.00768 Da; 23.4 PPM | Integrated database | C17H14O7 | M-H | Flavonoids |
| 95 | 12085264 | Pilosin | 329.1 | 313.1 | 7.93 | 3 | 0.52 | ΔMZ=0.00758 Da; 23.0 PPM | Integrated database | C17H14O7 | M-H | Flavonoids |
| 96 | 21120349 | 2-Galloyl-D-glucose | 331.1 | 271.0 | 6.09 | 3 | 0.87 | ΔMZ=0.00014 Da; 0.4 PPM | Integrated database | C13H16O10 | M-H | Hydrolysable tannins |
| 97 | 14345564 | 3-Glucogallic acid | 331.1 | 313.1 | 2.17 | 3 | 0.82 | ΔMZ=0.00005 Da; 0.2 PPM | Integrated database | C13H16O10 | M-H | Phenolic acids |
| 98 | 14428089 | 4-Glucosyl gallate | 331.1 | 273.1 | 10.19 | 3 | 0.89 | ΔMZ=0.00017 Da; 0.5 PPM | Integrated database | C13H16O10 | M-H | Hydrolysable tannins |
| 99 | 124202066 | 4-Hydroxy-5-(dihydroxyphenyl)-valeric acid-O-methyl-O-sulphate | 335.0 | 184.0 | 8.45 | 3 | 0.92 | ΔMZ=0.00299 Da; 8.9 PPM | Integrated database | C12H16O9S | M-H | Phenolic acids |
| 100 | 54676038 | Dicumarol | 335.1 | 299.1 | 9.09 | 2 | 0.31 | ΔMZ=0.00206 Da; 6.1 PPM | KEGG database | C19H12O6 | M-H | Coumarins |
| 101 | 6441280 | 3-p-Coumaroylquinic acid | 337.1 | 191.1 | 4.04 | 3 | 0.85 | ΔMZ=0.00690 Da; 20.5 PPM | Integrated database | C16H18O8 | M-H | Phenolic acids |
| 102 | 78012803 | Hydrojuglone glucoside | 337.1 | 173.0 | 5.37 | 3 | 0.52 | ΔMZ=0.00001 Da; 0.0 PPM | Integrated database | C16H18O8 | M-H | Quinones |
| 103 | 5281766 | 4-p-Coumaroylquinic acid | 337.1 | 191.1 | 5.35 | 2 | 0.79 | ΔMZ=0.00045 Da; 1.3 PPM | KEGG database | C16H18O8 | M-H | Phenolic acids |
| 104 | 78190001 | α-Hydrojuglone 4-O-b-D-glucoside | 337.1 | 176.0 | 5.48 | 3 | 0.94 | ΔMZ=0.00018 Da; 0.5 PPM | Integrated database | C16H18O8 | M-H | Quinones |
| 105 | 5281761 | 1-Caffeoyl-β-D-glucose | 341.1 | 179.0 | 0.85 | 2 | 0.83 | ΔMZ=0.00011 Da; 0.3 PPM | KEGG database | C15H18O9 | M-H | Phenolic acids |
| 106 | 6148082 | Glucocaffeic acid | 341.1 | 179.0 | 4.10 | 3 | 0.79 | ΔMZ=0.00005 Da; 0.2 PPM | Integrated database | C15H18O9 | M-H | Phenolic acids |
| 107 | 131751630 | 4',6'-Dihydroxy-2'-methoxyacetophenone 6'-glucoside | 343.1 | 283.1 | 9.10 | 3 | 0.70 | ΔMZ=0.00019 Da; 0.5 PPM | Integrated database | C15H20O9 | M-H | Phenolic aldehydes/ketones |
| 108 | 5281695 | Santin | 343.1 | 313.1 | 7.59 | 3 | 0.43 | ΔMZ=0.00679 Da; 19.8 PPM | Integrated database | C18H16O7 | M-H | Flavonoids |
| 109 | 329584 | 3,3'-Bisjuglone | 345.0 | 301.0 | 5.19 | 3 | 0.50 | ΔMZ=0.00579 Da; 16.8 PPM | Integrated database | C20H10O6 | M-H | Quinones |
| 110 | 53297447 | α-(1,2-Dihydroxyethyl)-1,2,3,4-tetrahydro-7-hydroxy-9-methoxy-3,4-dioxocyclopenta[c][1]benzopyran-6-acetaldehyde | 347.1 | 315.1 | 5.64 | 2 | 0.41 | ΔMZ=0.00006 Da; 0.2 PPM | KEGG database | C17H16O8 | M-H | Quinones |
| 111 | 131751960 | 6''-O-Acetylholocalin | 352.1 | 232.1 | 9.02 | 3 | 0.42 | ΔMZ=0.00009 Da; 0.2 PPM | Integrated database | C16H19NO8 | M-H | Flavonoids |
| 112 | 1794427 | Chlorogenic Acid | 353.1 | 192.1 | 0.86 | 3 | 0.81 | ΔMZ=0.00009 Da; 0.3 PPM | Integrated database | C16H18O9 | M-H | Phenolic acids |
| 113 | 12302892 | (+)-Chebulic acid | 355.0 | 338.0 | 1.56 | 3 | 0.41 | ΔMZ=0.00000 Da; 0.0 PPM | Integrated database | C14H12O11 | M-H | Hydrolysable tannins |
| 114 | 13962927 | 1-O-Feruloylglucose | 355.1 | 194.1 | 6.72 | 3 | 0.91 | ΔMZ=0.00033 Da; 0.9 PPM | Integrated database | C16H20O9 | M-H | Phenolic acids |
| 115 | 23725 | Psoromic acid | 357.1 | 183.0 | 9.62 | 3 | 0.92 | ΔMZ=0.00007 Da; 0.2 PPM | Integrated database | C18H14O8 | M-H | Other phenolics |
| 116 |  | 1-O-(3-Hydroxy-4,5-dimethoxybenzoyl)hexopyranose | 359.1 | 313.1 | 0.84 | 3 | 0.69 | ΔMZ=0.00029 Da; 0.8 PPM | Integrated database | C15H20O10 | M-H | Phenolic acids |
| 117 | 100986273 | 6'-Methoxypolygoacetophenoside | 359.1 | 313.1 | 0.83 | 3 | 0.73 | ΔMZ=0.00014 Da; 0.4 PPM | Integrated database | C15H20O10 | M-H | Phenolic aldehydes/ketones |
| 118 | 65373 | Secoisolariciresinol | 361.2 | 346.1 | 12.55 | 2 | 0.36 | ΔMZ=0.00026 Da; 0.7 PPM | KEGG database | C20H26O6 | M-H | Lignans |
| 119 | 11760306 | 1-Hydroxy-4-[(2S,3R,4S,5S,6R)-3,4,5-trihydroxy-6-(hydroxymethyl)oxan-2-yl]oxynaphthalene-2-carboxylic acid | 365.1 | 275.1 | 7.21 | 3 | 0.82 | ΔMZ=0.00020 Da; 0.5 PPM | Integrated database | C17H18O9 | M-H | Quinones |
| 120 | 131752769 | 3-O-Caffeoyl-4-O-methylquinic acid | 367.1 | 301.0 | 4.54 | 3 | 0.78 | ΔMZ=0.00251 Da; 6.8 PPM | Integrated database | C17H20O9 | M-H | Phenolic acids |
| 121 | 9799386 | 3-Feruloylquinic acid | 367.1 | 301.0 | 4.98 | 3 | 0.79 | ΔMZ=0.00037 Da; 1.0 PPM | Integrated database | C17H20O9 | M-H | Phenolic acids |
| 122 | 443019 | Sesamolinol | 371.1 | 325.1 | 0.85 | 2 | 0.69 | ΔMZ=0.00581 Da; 15.7 PPM | KEGG database | C20H20O7 | M-H | Lignans |
| 123 | 13870578 | Fragransin C1 | 373.2 | 179.1 | 5.65 | 3 | 1.00 | ΔMZ=0.00032 Da; 0.9 PPM | Integrated database | C21H26O6 | M-H | Lignans |
| 124 |  | 3,5,7-Trihydroxy-2-(3-hydroxy-4-methoxyphenyl)-6,8-dimethoxy-4H-chromen-4-one | 375.1 | 201.0 | 2.02 | 3 | 0.71 | ΔMZ=0.00278 Da; 7.4 PPM | Integrated database | C18H16O9 | M-H | Flavonoids |
| 125 | 12311234 | Limocitrol | 375.1 | 201.0 | 4.11 | 3 | 0.71 | ΔMZ=0.00290 Da; 7.7 PPM | Integrated database | C18H16O9 | M-H | Flavonoids |
| 126 | 363452 | Naphthoherniarin | 375.1 | 177.1 | 9.82 | 3 | 0.70 | ΔMZ=0.00239 Da; 6.4 PPM | Integrated database | C22H16O6 | M-H | Coumarins |
| 127 | 23900015 | Geshoidin | 377.1 | 341.1 | 0.84 | 3 | 0.77 | ΔMZ=0.00229 Da; 6.1 PPM | Integrated database | C18H18O9 | M-H | Coumarins |
| 128 | 6168296 | 1-O-Sinapoylglucose | 385.1 | 177.1 | 7.50 | 3 | 0.88 | ΔMZ=0.00012 Da; 0.3 PPM | Integrated database | C17H22O10 | M-H | Phenolic acids |
| 129 | 131751962 | 2,4,6-Phenanthrenetriol 2-O-b-D-glucoside | 387.1 | 342.1 | 0.85 | 3 | 0.42 | ΔMZ=0.00573 Da; 14.8 PPM | Integrated database | C20H20O8 | M-H | Other phenolics |
| 130 | 21593928 | 4'-Hydroxy-3',5,6,7,8-pentamethoxyflavone | 387.1 | 342.1 | 0.85 | 3 | 0.66 | ΔMZ=0.00618 Da; 16.0 PPM | Integrated database | C20H20O8 | M-H | Flavonoids |
| 131 | 10200272 | 5-Hydroxy-3,3',4',7,8-pentamethoxyflavone | 387.1 | 342.1 | 0.85 | 3 | 0.71 | ΔMZ=0.00593 Da; 15.3 PPM | Integrated database | C20H20O8 | M-H | Flavonoids |
| 132 | 23757095 | 1a,2,3,4,5b,11,11a,11b-Octahydro-10,11,11a-trihydroxy-3-methyl-benz[3,4]anthra[1,2-b]oxirene-5,6-dione | 387.1 | 342.1 | 0.85 | 3 | 0.96 | ΔMZ=0.00577 Da; 14.9 PPM | Integrated database | C19H18O6 | M+FA-H | Quinones |
| 133 | 197678 | Shinflavanone | 389.2 | 301.0 | 5.38 | 3 | 0.44 | ΔMZ=0.00616 Da; 15.8 PPM | Integrated database | C25H26O4 | M-H | Flavonoids |
| 134 | 11003703 | Garcimangosone D | 391.1 | 281.1 | 5.98 | 3 | 0.83 | ΔMZ=0.00004 Da; 0.1 PPM | Integrated database | C19H20O9 | M-H | Flavonoids |
| 135 | 131753170 | Vitisidin A | 398.1 | 322.1 | 6.70 | 3 | 0.86 | ΔMZ=0.00265 Da; 6.7 PPM | Integrated database | C20H15O9 | M-H | Flavonoids |
| 136 | 124202072 | 1-O-Sinapoylglucose | 401.1 | 356.1 | 5.74 | 3 | 0.86 | ΔMZ=0.00016 Da; 0.4 PPM | Integrated database | C17H22O11 | M-H | Phenolic acids |
| 137 | 500825926 | Sericetin | 403.2 | 343.1 | 5.73 | 3 | 0.55 | ΔMZ=0.00564 Da; 14.0 PPM | Integrated database | C25H24O5 | M-H | Flavonoids |
| 138 | 56776287 | 3,5-Dihydroxy-2-(4-hydroxyphenyl)-3,4-dihydro-2H-chromen-7-yl pentofuranoside | 405.1 | 282.1 | 7.87 | 3 | 0.47 | ΔMZ=0.00014 Da; 0.3 PPM | Integrated database | C20H22O9 | M-H | Flavonoids |
| 139 | 13888255 | Afzelechin 7-apioside | 405.1 | 243.1 | 7.46 | 3 | 0.73 | ΔMZ=0.00015 Da; 0.4 PPM | Integrated database | C20H22O9 | M-H | Flavonoids |
| 140 | 3084295 | Poliothyrsoside; Nigracin | 405.1 | 329.1 | 7.29 | 3 | 0.94 | ΔMZ=0.00037 Da; 0.9 PPM | Integrated database | C20H22O9 | M-H | Lignans |
| 141 | 10319154 | Kanzonol Z | 405.2 | 329.1 | 7.28 | 3 | 0.83 | ΔMZ=0.00225 Da; 5.6 PPM | Integrated database | C25H26O5 | M-H | Flavonoids |
| 142 | 25245324 | Xanthohumol E | 405.2 | 345.2 | 11.43 | 3 | 0.61 | ΔMZ=0.00586 Da; 14.5 PPM | Integrated database | C25H26O5 | M-H | Flavonoids |
| 143 | 637213 | Rhaponticin | 419.1 | 307.1 | 16.15 | 2 | 0.93 | ΔMZ=0.00007 Da; 0.2 PPM | KEGG database | C21H24O9 | M-H | Stilbenoids |
| 144 | 51136539 | Catechin 7-arabinofuranoside | 421.1 | 281.1 | 6.10 | 3 | 0.84 | ΔMZ=0.00019 Da; 0.5 PPM | Integrated database | C20H22O10 | M-H | Flavonoids |
| 145 | 20979907 | Rumexoside | 421.1 | 311.1 | 14.99 | 3 | 0.57 | ΔMZ=0.00003 Da; 0.1 PPM | Integrated database | C20H22O10 | M-H | Stilbenoids |
| 146 | 325518 | Cajanone | 421.2 | 385.2 | 7.88 | 3 | 0.41 | ΔMZ=0.00228 Da; 5.4 PPM | Integrated database | C25H26O6 | M-H | Flavonoids |
| 147 | 11964494 | 3-(2,4-Dihydroxyphenyl)-5,7-dihydroxy-6,8-bis(3-methylbut-2-enyl)-2,3-dihydrochromen-4-one | 423.2 | 387.2 | 9.04 | 3 | 0.97 | ΔMZ=0.00292 Da; 6.9 PPM | Integrated database | C25H28O6 | M-H | Flavonoids |
| 148 | 10342292 | Kuwanon E | 423.2 | 243.1 | 5.25 | 3 | 0.98 | ΔMZ=0.00594 Da; 14.0 PPM | Integrated database | C25H28O6 | M-H | Flavonoids |
| 149 | 275535139 | Orientanol E; 3-(2,4-Dihydroxyphenyl)-5,7-dihydroxy-6,8-bis(3-methyl-2-buten-1-yl)-2,3-dihydro-4H-chromen-4-one | 423.2 | 379.0 | 7.19 | 3 | 0.96 | ΔMZ=0.00228 Da; 5.4 PPM | Integrated database | C25H28O6 | M-H | Flavonoids |
| 150 | 11968848 | Quercetin-3-O-α-d-arabinofuranoside | 433.0 | 410.5 | 4.27 | 3 | 1.00 | ΔMZ=0.00629 Da; 14.5 PPM | Integrated database | C20H18O11 | M-H | Flavonoids |
| 151 | 6441059 | (2R,3S)-2-[(3,4-Dihydroxyphenyl)methyl]-3-[(E)-3-(3,4-dihydroxyphenyl)prop-2-enoyl]oxy-2-hydroxybutanedioic acid | 433.1 | 313.1 | 10.23 | 3 | 0.86 | ΔMZ=0.00021 Da; 0.5 PPM | Integrated database | C20H18O11 | M-H | Flavonoids |
| 152 | 131751154 | 2-O-Caffeoylarbutin | 433.1 | 313.1 | 10.90 | 3 | 0.84 | ΔMZ=0.00004 Da; 0.1 PPM | Integrated database | C21H22O10 | M-H | Phenolic acids |
| 153 | 5490064 | Avicularin | 433.1 | 415.2 | 11.21 | 3 | 0.81 | ΔMZ=0.00007 Da; 0.2 PPM | Integrated database | C20H18O11 | M-H | Flavonoids |
| 154 | 9910767 | 5-Hydroxy-2-(4-hydroxyphenyl)-7-[(2S,3R,4S,5S,6R)-3,4,5-trihydroxy-6-(hydroxymethyl)oxan-2-yl]oxy-2,3-dihydrochromen-4-one | 433.1 | 313.1 | 6.09 | 3 | 0.90 | ΔMZ=0.00604 Da; 13.9 PPM | Integrated database | C21H22O10 | M-H | Flavonoids |
| 155 | 40473202 | (-)-Naringenin 7-β-D-glucoside | 433.1 | 387.2 | 10.88 | 3 | 1.00 | ΔMZ=0.00390 Da; 9.0 PPM | Integrated database | C21H22O10 | M-H | Flavonoids |
| 156 | 92794 | Prunin | 433.1 | 387.2 | 5.03 | 3 | 1.00 | ΔMZ=0.00603 Da; 13.9 PPM | Integrated database | C21H22O10 | M-H | Flavonoids |
| 157 | 21629801 | (-)-Epigallocatechin 3-cinnamate | 435.1 | 389.1 | 5.42 | 3 | 0.55 | ΔMZ=0.00009 Da; 0.2 PPM | Integrated database | C24H20O8 | M-H | Flavonoids |
| 158 | 5491388 | Homomangiferin | 435.1 | 341.1 | 7.32 | 3 | 0.86 | ΔMZ=0.00026 Da; 0.6 PPM | Integrated database | C20H20O11 | M-H | Flavonoids |
| 159 | 46915712 | Isochinomin | 435.1 | 417.1 | 6.43 | 3 | 0.52 | ΔMZ=0.00009 Da; 0.2 PPM | Integrated database | C20H20O11 | M-H | Coumarins |
| 160 | 9912668 | Phloridzin | 435.1 | 297.1 | 11.90 | 1 | 0.70 | ΔMZ=0.00081 Da; 1.9 PPM | Standards database | C21H24O10 | M-H | Phenolic acids |
| 161 | 131753138 | Taxifolin 3-arabinoside | 435.1 | 301.0 | 5.62 | 3 | 0.43 | ΔMZ=0.00015 Da; 0.3 PPM | Integrated database | C20H20O11 | M-H | Flavonoids |
| 162 | 6451798 | Trilobatin | 435.1 | 303.1 | 6.42 | 3 | 0.49 | ΔMZ=0.00015 Da; 0.3 PPM | Integrated database | C21H24O10 | M-H | Flavonoids |
| 163 | 6072 | Phlorizin | 436.1 | 303.0 | 5.63 | 2 | 0.56 | ΔMZ=0.00417 Da; 9.6 PPM | KEGG database | C21H24O10 | M-H | Phenolic acids |
| 164 | 156269 | Loquatoside | 437.1 | 329.1 | 8.22 | 3 | 0.41 | ΔMZ=0.00012 Da; 0.3 PPM | Integrated database | C20H22O11 | M-H | Flavonoids |
| 165 | 471393 | 7-O-Galloylcatechin | 441.1 | 289.1 | 6.30 | 3 | 0.97 | ΔMZ=0.00027 Da; 0.6 PPM | Integrated database | C22H18O10 | M-H | Hydrolysable tannins |
| 166 | 44259088 | Kaempferide 3-rhamnoside | 445.1 | 307.1 | 6.71 | 3 | 0.67 | ΔMZ=0.00011 Da; 0.3 PPM | Integrated database | C22H22O10 | M-H | Flavonoids |
| 167 |  | 2-(3,4-Dihydroxyphenyl)-5,7-dihydroxy-3-[(3,4,5-trihydroxy-6-methyloxan-2-yl)oxy]-4H-chromen-4-one | 447.1 | 302.0 | 11.22 | 3 | 0.85 | ΔMZ=0.00649 Da; 14.5 PPM | Integrated database | C21H20O11 | M-H | Hydrolysable tannins |
| 168 | 165340543 | (2R,3S)-2,3-Dihydro-5,5a(2),7,7a(2)-tetrahydroxy-2-(4-hydroxyphenyl)[3,8a(2)-bi-4H-1-benzopyran]-4,4a(2)-dione | 447.1 | 323.0 | 17.61 | 3 | 0.87 | ΔMZ=0.00011 Da; 0.2 PPM | Integrated database | C24H16O9 | M-H | Flavonoids |
| 169 | 131752855 | Aromadendrin 4'-methyl ether 7-rhamnoside | 447.1 | 285.1 | 6.53 | 3 | 0.80 | ΔMZ=0.00004 Da; 0.1 PPM | Integrated database | C22H24O10 | M-H | Flavonoids |
| 170 | 10095180 | Kaempferol 7-O-β-D-glucopyranoside | 447.1 | 301.0 | 9.07 | 1 | 0.65 | ΔMZ=0.00111 Da; 2.5 PPM | Standards database | C21H20O11 | M-H | Flavonoids |
| 171 | 5748601 | Quercetin 7-O-rhamnoside | 447.1 | 302.0 | 3.92 | 3 | 0.59 | ΔMZ=0.00080 Da; 1.8 PPM | Integrated database | C21H20O11 | M-H | Flavonoids |
| 172 | 114776 | Isoorientin | 448.1 | 302.0 | 9.06 | 2 | 0.85 | ΔMZ=0.00399 Da; 8.9 PPM | KEGG database | C21H20O11 | M-H | Flavonoids |
| 173 | 119258 | Astilbin | 449.1 | 303.1 | 9.01 | 2 | 0.69 | ΔMZ=0.00002 Da; 0.0 PPM | KEGG database | C21H22O11 | M-H | Flavonoids |
| 174 | 10797 | Chrysanthemin | 449.1 | 311.1 | 8.94 | 2 | 0.62 | ΔMZ=0.00003 Da; 0.1 PPM | KEGG database | C21H21O11 | M-H | Flavonoids |
| 175 | 101781 | Flavanomarein | 449.1 | 431.2 | 10.02 | 3 | 0.54 | ΔMZ=0.00027 Da; 0.6 PPM | Integrated database | C21H22O11 | M-H | Flavonoids |
| 176 | 10765955 | Maesopsin 6-glucoside | 449.1 | 308.0 | 6.51 | 3 | 0.66 | ΔMZ=0.00076 Da; 1.7 PPM | Integrated database | C21H22O11 | M-H | Flavonoids |
| 177 | 6441269 | Marein | 449.1 | 308.0 | 6.51 | 3 | 0.48 | ΔMZ=0.00026 Da; 0.6 PPM | Integrated database | C21H22O11 | M-H | Flavonoids |
| 178 | 46173973 | Neocarthamin | 449.1 | 389.2 | 11.99 | 3 | 0.64 | ΔMZ=0.00013 Da; 0.3 PPM | Integrated database | C21H22O11 | M-H | Flavonoids |
| 179 | 9889961 | Phloretin 2'-O-glucuronide | 449.1 | 303.1 | 11.40 | 3 | 0.50 | ΔMZ=0.00030 Da; 0.7 PPM | Integrated database | C21H22O11 | M-H | Phenolic acids |
| 180 | 6474788 | (-)-Epigallocatechin 3-p-coumaroate | 451.1 | 342.1 | 9.00 | 3 | 0.67 | ΔMZ=0.00024 Da; 0.5 PPM | Integrated database | C24H20O9 | M-H | Flavonoids |
| 181 | 25796664 | [(2R,3S,4S,5R,6S)-6-(3,4-Dihydroxybenzoyl)oxy-3,4,5-trihydroxyoxan-2-yl]methyl 3,4-dihydroxybenzoate | 451.1 | 299.1 | 7.89 | 3 | 0.50 | ΔMZ=0.00002 Da; 0.1 PPM | Integrated database | C20H20O12 | M-H | Phenolic acids |
| 182 | 75411984 | 1,5,8-Trihydroxy-4-methoxy-3-[(2S,3R,4S,5S,6R)-3,4,5-trihydroxy-6-(hydroxymethyl)oxan-2-yl]oxyxanthen-9-one | 451.1 | 405.2 | 12.79 | 3 | 0.49 | ΔMZ=0.00004 Da; 0.1 PPM | Integrated database | C20H20O12 | M-H | Xanthones |
| 183 |  | 1,5,8-Trihydroxy-4-methoxy-9-oxo-9H-xanthen-3-yl-D-glucopyranoside | 451.1 | 405.2 | 7.02 | 3 | 0.49 | ΔMZ=0.00013 Da; 0.3 PPM | Integrated database | C20H20O12 | M-H | Xanthones |
| 184 | 73533 | (+)-Catechin 7-O-β-D-xyloside | 451.1 | 341.1 | 6.72 | 2 | 0.70 | ΔMZ=0.00011 Da; 0.3 PPM | KEGG database | C20H22O10 | M-H | Flavonoids |
| 185 | 131751470 | Viniferifuran | 451.1 | 341.1 | 6.75 | 3 | 0.85 | ΔMZ=0.00581 Da; 12.9 PPM | Integrated database | C28H20O6 | M-H | Lignans |
| 186 | 53398699 | 6''-O-Acetyldaidzin | 457.1 | 281.1 | 7.78 | 3 | 0.76 | ΔMZ=0.00021 Da; 0.5 PPM | Integrated database | C23H22O10 | M-H | Flavonoids |
| 187 | 131752779 | 5,7-Dihydroxy-2',6-dimethoxyisoflavone 7-rhamnoside | 459.1 | 307.1 | 6.91 | 3 | 0.60 | ΔMZ=0.00002 Da; 0.0 PPM | Integrated database | C23H24O10 | M-H | Flavonoids |
| 188 | 14034234 | 6-Cinnamoyl-1-galloylglucose | 461.1 | 313.1 | 11.45 | 3 | 0.81 | ΔMZ=0.00015 Da; 0.3 PPM | Integrated database | C22H22O11 | M-H | Hydrolysable tannins |
| 189 | 442659 | Swertiajaponin | 461.1 | 235.1 | 7.67 | 2 | 0.94 | ΔMZ=0.00023 Da; 0.5 PPM | KEGG database | C22H22O11 | M-H | Flavonoids |
| 190 | 38358688 | (3-hydroxy-4-{[(2S,3R,4S,5S,6R)-3,4,5-trihydroxy-6-(hydroxymethyl)oxan-2-yl]oxy}phenyl)methyl 4-hydroxy-3,5-dimethoxybenzoate | 463.1 | 323.1 | 8.28 | 3 | 0.77 | ΔMZ=0.00056 Da; 1.2 PPM | Integrated database | C22H26O12 | M-H2O-H | Phenolic acids |
| 191 | 443650 | Delphinidin 3-O-glucoside | 463.1 | 302.0 | 4.13 | 2 | 0.34 | ΔMZ=0.00057 Da; 1.2 PPM | KEGG database | C21H21O12 | M-2H | Flavonoids |
| 192 | 18625123 | Hesperetin 5-O-glucoside | 463.1 | 302.1 | 7.16 | 3 | 0.78 | ΔMZ=0.00000 Da; 0.0 PPM | Integrated database | C22H24O11 | M-H | Flavonoids |
| 193 | 147394 | Hesperetin 7-O-glucoside | 463.1 | 302.1 | 7.92 | 2 | 0.61 | ΔMZ=0.00015 Da; 0.3 PPM | KEGG database | C22H24O11 | M-H | Flavonoids |
| 194 | 5281673 | Myricitrin | 463.1 | 427.2 | 6.54 | 1 | 0.85 | ΔMZ=0.00072 Da; 1.5 PPM | Standards database | C21H20O12 | M-H | Flavonoids |
| 195 | 5280804 | Quercetin 3-D-glucoside | 463.1 | 302.0 | 3.41 | 1 | 0.75 | ΔMZ=0.00148 Da; 3.2 PPM | Standards database | C21H20O12 | M-H | Flavonoids |
| 196 | 76969982 | (-)-Epicatechin-3'-O-glucuronide | 465.1 | 447.1 | 4.63 | 3 | 0.44 | ΔMZ=0.00031 Da; 0.7 PPM | Integrated database | C21H22O12 | M-H | Flavonoids |
| 197 | 38359194 | (2S,3S)-3,5,7-Trihydroxy-2-[4-hydroxy-3-[(2S,3R,4S,5S,6R)-3,4,5-trihydroxy-6-(hydroxymethyl)oxan-2-yl]oxyphenyl]-2,3-dihydrochromen-4-one | 465.1 | 447.1 | 10.17 | 3 | 0.47 | ΔMZ=0.00038 Da; 0.8 PPM | Integrated database | C21H22O12 | M-H | Flavonoids |
| 198 | 441663 | Silandrin | 465.1 | 451.0 | 5.65 | 2 | 0.55 | ΔMZ=0.00577 Da; 12.4 PPM | KEGG database | C25H22O9 | M-H | Flavonoids |
| 199 | 136784551 | Sanguisorbic acid dilactone | 469.0 | 460.0 | 4.72 | 3 | 0.62 | ΔMZ=0.00014 Da; 0.3 PPM | Integrated database | C21H10O13 | M-H | Hydrolysable tannins |
| 200 | 5282102 | Kaempferol-3-glucoside | 469.1 | 447.0 | 4.87 | 1 | 0.75 | ΔMZ=0.00932 Da; 19.9 PPM | Standards database | C21H20O11 | M+Na-2H | Flavonoids |
| 201 | 9804842 | (-)-Epigallocatechin 3-(3-methyl-gallate) | 471.1 | 435.1 | 7.02 | 3 | 0.64 | ΔMZ=0.00060 Da; 1.3 PPM | Integrated database | C23H20O11 | M-H | Hydrolysable tannins |
| 202 |  | 2-({6-O-[(2E)-3-(4-Hydroxyphenyl)-2-propenoyl]-D-glucopyranosyl}oxy)-3-phenylacrylic acid | 471.1 | 307.1 | 7.41 | 3 | 0.93 | ΔMZ=0.00569 Da; 12.1 PPM | Integrated database | C24H24O10 | M-H | Phenolic acids |
| 203 | 90470472 | m-Trigallic acid | 473.0 | 457.5 | 6.07 | 3 | 0.54 | ΔMZ=0.00075 Da; 1.6 PPM | Integrated database | C21H14O13 | M-H | Hydrolysable tannins |
| 204 | 184999 | Orientin 7,3'-dimethyl ether | 475.1 | 313.1 | 6.87 | 3 | 0.81 | ΔMZ=0.00036 Da; 0.8 PPM | Integrated database | C23H24O11 | M-H | Flavonoids |
| 205 | 131750802 | 4'-O-methyl-(-)-epicatechin-7-O-β-glucuronide | 477.1 | 473.0 | 8.58 | 3 | 0.89 | ΔMZ=0.00010 Da; 0.2 PPM | Integrated database | C23H26O11 | M-H | Flavonoids |
| 206 | 176449 | Petunidin 3-O-glucoside | 477.1 | 265.1 | 13.59 | 2 | 0.97 | ΔMZ=0.00054 Da; 1.1 PPM | KEGG database | C22H23O12 | M-2H | Flavonoids |
| 207 | 131752619 | 1,2'-Di-O-galloylhamamelofuranose | 483.1 | 467.0 | 6.05 | 3 | 0.52 | ΔMZ=0.00018 Da; 0.4 PPM | Integrated database | C20H20O14 | M-H | Hydrolysable tannins |
| 208 | 3332212 | 1,6-Digalloyl-β-D-glucopyranose | 483.1 | 452.0 | 5.63 | 3 | 0.82 | ΔMZ=0.00001 Da; 0.0 PPM | Integrated database | C20H20O14 | M-H | Hydrolysable tannins |
| 209 |  | 2-(3,4-Dihydroxyphenyl)-5,7-dihydroxy-6-[3,4,5-trihydroxy-6-(hydroxymethyl)oxan-2-yl]-4H-chromen-4-one | 483.1 | 400.0 | 2.98 | 3 | 0.81 | ΔMZ=0.00776 Da; 16.1 PPM | Integrated database | C21H20O11 | M+Cl | Hydrolysable tannins |
| 210 | 54116668 | 2,6-Digalloylglucose | 483.1 | 332.1 | 3.56 | 3 | 0.78 | ΔMZ=0.00030 Da; 0.6 PPM | Integrated database | C20H20O14 | M-H | Hydrolysable tannins |
| 211 | 73157750 | Gallic acid 4-O-(6-galloylglucoside) | 483.1 | 331.1 | 3.58 | 3 | 0.72 | ΔMZ=0.00020 Da; 0.4 PPM | Integrated database | C20H20O14 | M-H | Hydrolysable tannins |
| 212 | 44257914 | 2''-O-Acetylisoorientin | 489.1 | 459.1 | 11.19 | 3 | 0.79 | ΔMZ=0.00005 Da; 0.1 PPM | Integrated database | C23H22O12 | M-H | Flavonoids |
| 213 | 131753196 | Irisolidone 7-O-glucuronide | 489.1 | 337.1 | 6.97 | 3 | 0.52 | ΔMZ=0.00022 Da; 0.4 PPM | Integrated database | C23H22O12 | M-H | Flavonoids |
| 214 | 74978029 | Kaempferol 3-(6-acetylgalactoside) | 489.1 | 327.1 | 6.17 | 3 | 0.64 | ΔMZ=0.00008 Da; 0.2 PPM | Integrated database | C23H22O12 | M-H | Flavonoids |
| 215 | 165366242 | Edulisin I | 489.2 | 301.0 | 3.78 | 3 | 0.91 | ΔMZ=0.00566 Da; 11.6 PPM | Integrated database | C28H26O8 | M-H | Lignans |
| 216 | 73357140 | Licoagroside A | 491.1 | 313.1 | 7.01 | 3 | 0.51 | ΔMZ=0.00023 Da; 0.5 PPM | Integrated database | C23H24O12 | M-H | Flavonoids |
| 217 | 94409 | Malvidin 3-galactoside | 491.1 | 323.1 | 6.78 | 3 | 0.85 | ΔMZ=0.00017 Da; 0.3 PPM | Integrated database | C23H25O12 | M-2H | Flavonoids |
| 218 | 14055563 | 2-O-Galloylsucrose | 493.1 | 332.1 | 10.10 | 3 | 0.83 | ΔMZ=0.00597 Da; 12.1 PPM | Integrated database | C19H26O15 | M-H | Hydrolysable tannins |
| 219 | 14055553 | 4'-O-Galloylsucrose | 493.1 | 449.0 | 5.44 | 3 | 0.83 | ΔMZ=0.00002 Da; 0.1 PPM | Integrated database | C19H26O15 | M-H | Hydrolysable tannins |
| 220 | 14055548 | 6'-O-Galloylsucrose | 493.1 | 481.1 | 8.05 | 3 | 0.72 | ΔMZ=0.00011 Da; 0.2 PPM | Integrated database | C19H26O15 | M-H | Hydrolysable tannins |
| 221 | 5281675 | Orientin | 493.1 | 341.1 | 5.76 | 2 | 0.44 | ΔMZ=0.00053 Da; 1.1 PPM | KEGG database | C21H20O11 | M+FA-H | Flavonoids |
| 222 | 9917512 | Pimentol | 493.1 | 341.1 | 8.07 | 3 | 0.56 | ΔMZ=0.00020 Da; 0.4 PPM | Integrated database | C23H26O12 | M-H | Flavonoids |
| 223 | 76972097 | 4'-Methyl-(-)-epigallocatechin 3'-glucuronide | 495.1 | 477.1 | 8.83 | 3 | 0.53 | ΔMZ=0.00007 Da; 0.2 PPM | Integrated database | C22H24O13 | M-H | Flavonoids |
| 224 | 74083657 | Lyoniresinol 9'-sulfate | 499.1 | 463.1 | 10.15 | 3 | 0.40 | ΔMZ=0.00364 Da; 7.3 PPM | Integrated database | C22H28O11S | M-H | Lignans |
| 225 | 73077146 | (S)-Multifidol 2-[apiosyl-(1->6)-glucoside] | 503.2 | 193.0 | 7.81 | 3 | 0.99 | ΔMZ=0.00026 Da; 0.5 PPM | Integrated database | C22H32O13 | M-H | Lignans |
| 226 | 57509548 | 3,4,5-Trihydroxy-6-[5-hydroxy-2-(4-hydroxy-3-methoxyphenyl)-3-methoxy-4-oxochromen-7-yl]oxyoxane-2-carboxylic acid | 505.1 | 353.1 | 5.86 | 3 | 1.00 | ΔMZ=0.00032 Da; 0.6 PPM | Integrated database | C23H22O13 | M-H | Lignans |
| 227 | 131752878 | Isobiflorin 6''-gallate | 505.1 | 353.1 | 4.99 | 3 | 0.44 | ΔMZ=0.00005 Da; 0.1 PPM | Integrated database | C23H22O13 | M-H | Hydrolysable tannins |
| 228 | 60208892 | 5,8-Dihydroxy-4-oxo-1,2,3,4-tetrahydro-1-naphthalenyl 6-O-(3,4,5-trihydroxybenzoyl)-β-D-glucopyranoside | 507.1 | 331.1 | 8.34 | 3 | 0.68 | ΔMZ=0.00051 Da; 1.0 PPM | Integrated database | C23H24O13 | M-H | Flavonoids |
| 229 | 131752227 | 4',8-Dimethylgossypetin 3-glucoside | 507.1 | 487.2 | 12.89 | 3 | 0.91 | ΔMZ=0.00058 Da; 1.1 PPM | Integrated database | C23H24O13 | M-H | Flavonoids |
| 230 | 51136399 | 5-Hydroxy-3-[4-hydroxy-2-[(2S,3R,4S,5S,6R)-3,4,5-trihydroxy-6-(hydroxymethyl)oxan-2-yl]oxyphenyl]-7-methoxychromen-4-one | 507.1 | 345.2 | 9.77 | 3 | 0.53 | ΔMZ=0.00049 Da; 1.0 PPM | Integrated database | C22H22O11 | M+FA-H | Flavonoids |
| 231 | 5281708 | Daidzein | 507.1 | 323.1 | 11.40 | 1 | 0.97 | ΔMZ=0.00490 Da; 9.7 PPM | Standards database | C15H10O4 | 2M-H | Flavonoids |
| 232 | 5320958 | Rheidin B | 507.1 | 332.1 | 1.46 | 3 | 0.50 | ΔMZ=0.00998 Da; 19.7 PPM | Integrated database | C30H20O8 | M-H | Anthraquinones |
| 233 | 14539906 | Spinacetin 3-glucoside | 507.1 | 489.1 | 5.36 | 3 | 0.93 | ΔMZ=0.00032 Da; 0.6 PPM | Integrated database | C23H24O13 | M-H | Flavonoids |
| 234 | 131752438 | (7'R,8'R)-4,7'-Epoxy-3'-methoxy-4',5,9,9'-lignanetetrol 9'-glucoside | 507.2 | 489.2 | 8.15 | 3 | 0.44 | ΔMZ=0.00059 Da; 1.2 PPM | Integrated database | C25H32O11 | M-H | Lignans |
| 235 | 5320384 | Palmidin A | 509.1 | 485.5 | 9.15 | 3 | 0.54 | ΔMZ=0.00575 Da; 11.3 PPM | Integrated database | C30H22O8 | M-H | Hydrolysable tannins |
| 236 | 14558383 | (7'R,8'R)-4,7'-Epoxy-3',5-dimethoxy-4',9,9'-lignanetriol 9'-glucoside | 521.2 | 331.1 | 6.93 | 3 | 0.57 | ΔMZ=0.00073 Da; 1.4 PPM | Integrated database | C26H34O11 | M-H | Lignans |
| 237 | 74407576 | Isolariciresinol 4'-O-β-D-glucoside | 521.2 | 331.1 | 6.93 | 3 | 0.61 | ΔMZ=0.00038 Da; 0.7 PPM | Integrated database | C26H34O11 | M-H | Lignans |
| 238 | 45360052 | 2-[4-[3-[3,4-Dihydroxy-4-(hydroxymethyl)oxolan-2-yl]oxy-4,5-dihydroxy-6-(hydroxymethyl)oxan-2-yl]oxyphenyl]-7-hydroxy-2,3-dihydrochromen-4-one | 531.2 | 337.1 | 7.90 | 3 | 0.43 | ΔMZ=0.00055 Da; 1.0 PPM | Integrated database | C26H30O13 | M-H2O-H | Phenolic acids |
| 239 |  | 2,4a,6-Trihydroxy-8-isopropyl-1,1-dimethyl-2,3,4,4a,5,10,11,11a-octahydro-1H-dibenzo[a,d][7]annulen-7-yl β-D-glucopyranoside | 541.3 | 495.3 | 10.50 | 3 | 0.92 | ΔMZ=0.00013 Da; 0.2 PPM | Integrated database | C26H40O9 | M+FA-H | Other phenolics |
| 240 | 147299 | Procyanidin B4 | 577.1 | 408.1 | 9.17 | 2 | 0.56 | ΔMZ=0.00045 Da; 0.8 PPM | KEGG database | C30H26O12 | M-H | Proanthocyanidins |
| 241 | 124017 | Procyanidin B5 | 577.1 | 407.1 | 8.26 | 2 | 0.81 | ΔMZ=0.00037 Da; 0.6 PPM | KEGG database | C30H26O12 | M-H | Proanthocyanidins |
| 242 | 474540 | Procyanidin B6 | 577.1 | 425.1 | 6.97 | 3 | 0.54 | ΔMZ=0.00050 Da; 0.9 PPM | Integrated database | C30H26O12 | M-H | Proanthocyanidins |
| 243 | 13990892 | Procyanidin B7 | 577.1 | 425.1 | 7.32 | 3 | 0.54 | ΔMZ=0.00026 Da; 0.5 PPM | Integrated database | C30H26O12 | M-H | Proanthocyanidins |
| 244 | 9851181 | Isorhoifolin | 577.2 | 313.1 | 12.56 | 3 | 1.00 | ΔMZ=0.00762 Da; 13.2 PPM | Integrated database | C27H30O14 | M-H | Flavonoids |
| 245 | 16203170 | Gambiriin A1 | 579.2 | 301.0 | 6.08 | 2 | 0.33 | ΔMZ=0.00015 Da; 0.3 PPM | KEGG database | C30H28O12 | M-H | Flavonoids |
| 246 | 24039364 | [(2R,3S,4S,5R,6S)-6-[3,5-Dihydroxy-4-[3-(4-hydroxyphenyl)propanoyl]phenoxy]-3,4,5-trihydroxyoxan-2-yl]methyl 3,4,5-trihydroxybenzoate | 587.1 | 425.1 | 15.40 | 3 | 0.69 | ΔMZ=0.00064 Da; 1.1 PPM | Integrated database | C28H28O14 | M-H | Flavonoids |
| 247 | 101223813 | 4'-Hydroxyanigorootin | 589.1 | 554.2 | 5.80 | 3 | 0.72 | ΔMZ=0.00349 Da; 5.9 PPM | Integrated database | C38H22O7 | M-H | Flavonoids |
| 248 | 45360286 | 5,7-Dihydroxy-2-(4-hydroxyphenyl)-3-[3,5,7-trihydroxy-2-(4-hydroxyphenyl)-3,4-dihydro-2H-chromen-8-yl]-2,3-dihydrochromen-4-one | 589.1 | 554.2 | 13.39 | 3 | 0.68 | ΔMZ=0.00191 Da; 3.2 PPM | Integrated database | C30H24O10 | M+FA-H | Flavonoids |
| 249 | 85270241 | Castacrenin A | 613.0 | 481.1 | 7.34 | 3 | 0.43 | ΔMZ=0.00104 Da; 1.7 PPM | Integrated database | C27H18O17 | M-H | Flavonoids |
| 250 | 85262147 | Castacrenin B | 613.0 | 391.5 | 4.10 | 3 | 0.49 | ΔMZ=0.00061 Da; 1.0 PPM | Integrated database | C27H18O17 | M-H | Flavonoids |
| 251 | 14034241 | 6-O-p-Coumaroyl-1,2-di-O-galloyl-b-D-glucopyranose | 629.1 | 477.1 | 12.69 | 3 | 0.75 | ΔMZ=0.00055 Da; 0.9 PPM | Integrated database | C29H26O16 | M-H | Phenolic acids |
| 252 | 74978295 | 2'-C-Methylmyricetin 3-rhamnoside 5'-gallate | 629.1 | 465.1 | 6.50 | 3 | 0.80 | ΔMZ=0.00046 Da; 0.7 PPM | Integrated database | C29H26O16 | M-H | Flavonoids |
| 253 | 131752572 | 2-O-(4-Hydroxycinnamoyl)-1,6-di-O-galloyl-β-D-glucopyranose | 629.1 | 567.2 | 11.40 | 3 | 0.57 | ΔMZ=0.00035 Da; 0.6 PPM | Integrated database | C29H26O16 | M-H | Hydrolysable tannins |
| 254 | 44259469 | Myricetin 7-(6''-galloylglucoside) | 631.1 | 613.0 | 5.28 | 3 | 0.88 | ΔMZ=0.00135 Da; 2.1 PPM | Integrated database | C28H24O17 | M-H | Flavonoids |
| 255 | 15698968 | Vescalin | 631.1 | 613.0 | 4.06 | 3 | 0.74 | ΔMZ=0.00037 Da; 0.6 PPM | Integrated database | C27H20O18 | M-H | Hydrolysable tannins |
| 256 | 71776765 | [(1S,21S,22R,23R)-6,7,8,11,12,13,22,23-Octahydroxy-3,16-dioxo-2,17,20-trioxatetracyclo[17.3.1.04,9.010,15]tricosa-4,6,8,10,12,14-hexaen-21-yl]3,4,5-trihydroxybenzoate | 633.1 | 463.1 | 9.71 | 3 | 0.71 | ΔMZ=0.00029 Da; 0.5 PPM | Integrated database | C27H22O18 | M-H | Lignans |
| 257 | 131752871 | 3'-(2'',3''-Digalloylglucosyl)-phloroacetophenone | 633.1 | 465.1 | 6.31 | 3 | 0.75 | ΔMZ=0.00037 Da; 0.6 PPM | Integrated database | C28H26O17 | M-H | Hydrolysable tannins |
| 258 | 131752872 | 3'-(2'',6''-Digalloylglucosyl)-phloroacetophenone | 633.1 | 465.1 | 15.23 | 3 | 0.93 | ΔMZ=0.00046 Da; 0.7 PPM | Integrated database | C28H26O17 | M-H | Hydrolysable tannins |
| 259 | 73568 | Corilagin | 633.1 | 301.0 | 11.22 | 2 | 0.95 | ΔMZ=0.00032 Da; 0.5 PPM | KEGG database | C27H22O18 | M-H | Hydrolysable tannins |
| 260 | 165367140 | Pterocaryanin B | 633.1 | 482.1 | 8.71 | 3 | 0.94 | ΔMZ=0.00073 Da; 1.1 PPM | Integrated database | C27H22O18 | M-H | Hydrolysable tannins |
| 261 | 472274129 | Punicacortein A | 633.1 | 481.1 | 8.42 | 3 | 0.91 | ΔMZ=0.00050 Da; 0.8 PPM | Integrated database | C27H22O18 | M-H | Hydrolysable tannins |
| 262 | 20056239 | Punicacortein B | 633.1 | 301.0 | 8.87 | 3 | 1.00 | ΔMZ=0.00022 Da; 0.3 PPM | Integrated database | C27H22O18 | M-H | Hydrolysable tannins |
| 263 | 13917512 | Sanguiin H4 | 633.1 | 481.1 | 2.62 | 3 | 0.84 | ΔMZ=0.00000 Da; 0.0 PPM | Integrated database | C27H22O18 | M-H | Hydrolysable tannins |
| 264 | 452707 | Gallotannin | 635.1 | 483.1 | 8.55 | 3 | 0.80 | ΔMZ=0.00010 Da; 0.2 PPM | Integrated database | C27H24O18 | M-H | Hydrolysable tannins |
| 265 | 5322038 | [(2R,3R,4S,5R,6S)-3-Hydroxy-2-(hydroxymethyl)-5,6-bis[(3,4,5-trihydroxybenzoyl)oxy]oxan-4-yl] 3,4,5-trihydroxybenzoate | 635.1 | 484.1 | 14.80 | 3 | 0.79 | ΔMZ=0.00029 Da; 0.5 PPM | Integrated database | C27H24O18 | M-H | Hydrolysable tannins |
| 266 |  | 1,2,3-Tris-O-(3,4,5-trihydroxybenzoyl)-D-glucopyranose | 635.1 | 483.1 | 8.57 | 3 | 0.78 | ΔMZ=0.00041 Da; 0.7 PPM | Integrated database | C27H24O18 | M-H | Hydrolysable tannins |
| 267 | 21722046 | Orotinichalcone | 635.1 | 483.1 | 8.55 | 3 | 0.981025647 | ΔMZ=0.00041 Da; 0.6 PPM | Integrated database | C27H24O18 | M-H | Hydrolysable tannins |
| 268 | 14284610 | 1,4,6-Trigalloyl-β-D-glucopyranose | 635.1 | 484.1 | 4.51 | 3 | 0.63 | ΔMZ=0.00041 Da; 0.6 PPM | Integrated database | C27H24O18 | M-H | Hydrolysable tannins |
| 269 | 440308 | 1,2,6-Trigalloylglucose | 635.1 | 483.1 | 7.59 | 2 | 0.90 | ΔMZ=0.00064 Da; 1.0 PPM | KEGG database | C27H24O18 | M-H | Hydrolysable tannins |
| 270 | 101317827 | Bisnorbadioquinone A | 649.1 | 606.1 | 2.94 | 3 | 0.51 | ΔMZ=0.00588 Da; 9.1 PPM | Integrated database | C34H18O14 | M-H | Quinones |
| 271 | 21159144 | Eujambin | 657.1 | 301.0 | 6.18 | 3 | 1.00 | ΔMZ=0.00045 Da; 0.7 PPM | Integrated database | C30H26O17 | M-H | Lignans |
| 272 | 5317340 | [6-[(5,7-Dihydroxy-4-methyl-1,2,3,4-tetrahydronaphthalen-2-yl)oxy]-4,5-dihydroxy-3-[(E)-3-(4-hydroxyphenyl)prop-2-enoyl]oxyoxan-2-yl]methyl (E)-3-(4-hydroxyphenyl)prop-2-enoate | 685.2 | 667.2 | 11.89 | 3 | 0.85 | ΔMZ=0.00682 Da; 9.9 PPM | Integrated database | C35H36O12 | M+K-2H | Flavonoids |
| 273 |  | α-D-Glucopyranoside, 3,6-bis-O-[(2E)-3-(4-hydroxy-3-methoxyphenyl)-1-oxo-2-propen-1-yl]-β-D-fructofuranosyl | 693.2 | 355.1 | 6.97 | 3 | 0.63 | ΔMZ=0.00173 Da; 2.5 PPM | Integrated database | C32H38O17 | M-H | Phenolic acids |
| 274 |  | 1,7-Bis(4-hydroxyphenyl)-3-heptanyl 2-O-[(2R,3R,4R)-3,4-dihydroxy-4-(hydroxymethyl)tetrahydro-2-furanyl]-6-O-[(2S,3R,4R)-3,4-dihydroxy-4-(hydroxymethyl)tetrahydro-2-furanyl]-D-glucopyranoside | 725.3 | 301.0 | 7.66 | 3 | 0.71 | ΔMZ=0.00049 Da; 0.7 PPM | Integrated database | C35H50O16 | M-H | Diarylheptanoids |
| 275 | 15593122 | ent-Epicatechin-(4α->8)-ent-epicatechin 3'-gallate | 729.1 | 603.1 | 5.55 | 3 | 0.81 | ΔMZ=0.00081 Da; 1.1 PPM | Integrated database | C37H30O16 | M-H | Proanthocyanidins |
| 276 | 12795889 | ent-Epicatechin-(4α->8)-ent-epicatechin 3-gallate | 729.1 | 451.1 | 3.49 | 3 | 0.49 | ΔMZ=0.00034 Da; 0.5 PPM | Integrated database | C37H30O16 | M-H | Proanthocyanidins |
| 277 | 14035439 | Castanin | 783.1 | 481.1 | 3.53 | 3 | 0.95 | ΔMZ=0.00095 Da; 1.2 PPM | Integrated database | C34H24O22 | M-H | Hydrolysable tannins |
| 278 | 165359404 | Granatin A | 783.1 | 481.1 | 3.53 | 3 | 0.93 | ΔMZ=0.00080 Da; 1.0 PPM | Integrated database | C34H24O22 | M-H | Hydrolysable tannins |
| 279 | 471120 | Heterophylliin A | 785.1 | 634.1 | 6.68 | 3 | 0.85 | ΔMZ=0.00152 Da; 1.9 PPM | Integrated database | C34H26O22 | M-H | Hydrolysable tannins |
| 280 | 14429413 | Sanguiin H1 | 785.1 | 633.1 | 6.48 | 3 | 0.87 | ΔMZ=0.00114 Da; 1.5 PPM | Integrated database | C34H26O22 | M-H | Hydrolysable tannins |
| 281 | 14411424 | 1,4-Di-O-galloyl-3,6-(R)-hexahydroxydiphenoyl-b-D-glucopyranose | 785.1 | 741.1 | 4.97 | 3 | 0.84 | ΔMZ=0.00064 Da; 0.8 PPM | Integrated database | C34H26O22 | M-H | Hydrolysable tannins |
| 282 | 471531 | 1,3,4,6-Tetra-O-galloyl glucose | 787.1 | 635.1 | 8.50 | 3 | 0.87 | ΔMZ=0.00102 Da; 1.3 PPM | Integrated database | C34H28O22 | M-H | Hydrolysable tannins |
| 283 | 5321843 | [(2R,3R,4S,5R,6S)-2-(Hydroxymethyl)-4,5,6-tris[(3,4,5-trihydroxybenzoyl)oxy]oxan-3-yl] 3,4,5-trihydroxybenzoate | 787.1 | 635.1 | 16.35 | 3 | 0.88 | ΔMZ=0.00078 Da; 1.0 PPM | Integrated database | C34H28O22 | M-H | Hydrolysable tannins |
| 284 | 73178 | [(2R,3R,4S,5R,6S)-3-Hydroxy-4,5,6-tris[(3,4,5-trihydroxybenzoyl)oxy]oxan-2-yl]methyl 3,4,5-trihydroxybenzoate | 787.1 | 635.1 | 7.10 | 3 | 0.82 | ΔMZ=0.00079 Da; 1.0 PPM | Integrated database | C34H28O22 | M-H | Hydrolysable tannins |
| 285 | 131752622 | 1,2',3,5-Tetra-O-galloylhamamelofuranose | 787.1 | 635.1 | 8.37 | 3 | 1.00 | ΔMZ=0.00135 Da; 1.7 PPM | Integrated database | C34H28O22 | M-H | Hydrolysable tannins |
| 286 | 5153644 | 1,2,3,6-Tetragalloyl-β-D-glucopyranose | 787.1 | 635.1 | 5.46 | 2 | 0.88 | ΔMZ=0.00084 Da; 1.1 PPM | KEGG database | C34H28O22 | M-H | Hydrolysable tannins |
| 287 | 21637585 | Punigluconin | 801.1 | 783.1 | 5.65 | 3 | 0.64 | ΔMZ=0.00127 Da; 1.6 PPM | Integrated database | C34H26O23 | M-H | Hydrolysable tannins |
| 288 | 131752595 | Sanguiin H7 | 801.1 | 783.1 | 5.65 | 3 | 0.55 | ΔMZ=0.00045 Da; 0.6 PPM | Integrated database | C34H26O23 | M-H | Hydrolysable tannins |
| 289 | 14824938 | Heterophylliin E | 907.1 | 889.1 | 3.99 | 3 | 0.50 | ΔMZ=0.00235 Da; 2.6 PPM | Integrated database | C40H28O25 | M-H | Hydrolysable tannins |
| 290 | 168165 | Vescalagin | 933.1 | 785.1 | 4.41 | 3 | 0.98 | ΔMZ=0.00147 Da; 1.6 PPM | Integrated database | C41H26O26 | M-H | Hydrolysable tannins |
| 291 | 73644 | Casuarictin | 935.1 | 917.1 | 6.16 | 2 | 0.89 | ΔMZ=0.00096 Da; 1.0 PPM | KEGG database | C41H28O26 | M-H | Hydrolysable tannins |
| 292 | 151590 | Eugeniin | 937.1 | 785.1 | 5.01 | 2 | 0.89 | ΔMZ=0.00085 Da; 0.9 PPM | KEGG database | C41H30O26 | M-H | Hydrolysable tannins |
| 293 | 85368941 | Sanguiin H11 | 951.1 | 908.1 | 4.04 | 3 | 0.58 | ΔMZ=0.00133 Da; 1.4 PPM | Integrated database | C41H28O27 | M-H | Hydrolysable tannins |

Table S5 Top 10 metabolite markers (by VIP score in PLS-DA) distinguishing walnut pellicles by region, with the region of highest expression for each metabolite.

| Index | Vip | BF mean | YN mean | XJ mean |
| --- | --- | --- | --- | --- |
| HMG | 2.18 | 570417.56 | 391297.63 | 205300.81 |
| 4-(Butoxymethyl)-2-methoxyphenol | 1.91 | 102504.13 | 519919.81 | 113348.26 |
| Phaseolic acid | 1.90 | 283143.62 | 1169420.06 | 458829.72 |
| DHC-MA | 1.80 | 13717.93 | 36144.09 | 11299.45 |
| Gambiriin A1 | 1.79 | 28137.96 | 83304.67 | 38626.95 |
| Casuarinin | 1.79 | 8569444.83 | 6888518.18 | 2827129.80 |
| Shinflavanone | 1.74 | 11373.70 | 26571.79 | 13486.37 |
| 1-O-p-Coumaroyl-β-D-glucose | 1.73 | 21096.04 | 92559.83 | 27815.18 |
| THMI | 1.69 | 9904.32 | 48596.57 | 20501.50 |
| Gentisate aldehyde | 1.63 | 55440.03 | 128078.30 | 67113.10 |

Note: 6-[4-hydroxy-2-methyl-6-[(2S,3R,4S,5S,6R)-3,4,5-trihydroxy-6-(hydroxymethyl)oxan-2-yl]oxyphenyl]-4-methoxypyran-2-one: HMG; 2R,3S)-2-[(3,4-dihydroxyphenyl)methyl]-3-[(E)-3-(3,4-dihydroxyphenyl)prop-2-enoyl]oxy-2-hydroxybutanedioic acid: DHC-MA; 4,6,8-Trihydroxy-7-methoxy-3-methyl-3,4-dihydro-1H-isochromen-1-one: THMI.

Table S6 Aggregated confusion matrix (true vs. predicted classes) for the three-class PLS-DA model (BF, YN, XJ) under stratified 7-fold CV (50 repeats).

| True\Pred | BF | YN | XJ | Row total |
| --- | --- | --- | --- | --- |
| BF | 1894 | 32 | 99 | 2025 |
| YN | 105 | 1468 | 2 | 1575 |
| XJ | 1 | 0 | 1649 | 1650 |
| Col total | 2000 | 1500 | 1750 | 5250 |

Table S7 Classification metrics derived from the cross-validated PLS-DA predictions.

| True\Pred | Recall / Sensitivity | Balanced accuracy | MCC (one-vs-rest) | Overall accuracy | Balanced accuracy |
| --- | --- | --- | --- | --- | --- |
| BF | 0.935 | 0.947 | 0.905 |  |  |
| YN | 0.932 | 0.979 | 0.937 |  |  |
| XJ | 0.999 | 0.942 | 0.957 |  |  |
| Macro / overall | 0.956 | 0.956 | 0.933 | 0.954 | 0.956 |

Table S8 Full list of p-values, q-values, and VIP scores

| Compounds | p-value | q-value | VIP | Significant  q＜0.05 |
| --- | --- | --- | --- | --- |
| 6-[4-Hydroxy-2-methyl-6-[(2S,3R,4S,5S,6R)-3,4,5-trihydroxy-6-(hydroxymethyl)oxan-2-yl]oxyphenyl]-4-methoxypyran-2-one | 1.1062E-14 | 4.49115E-12 | 2.18071773 | TRUE |
| 4-(Butoxymethyl)-2-methoxyphenol | 2.37295E-10 | 1.20427E-08 | 1.913258875 | TRUE |
| Phaseolic acid | 1.38537E-10 | 9.37432E-09 | 1.901183494 | TRUE |
| (2R,3S)-2-[(3,4-dihydroxyphenyl)methyl]-3-[(E)-3-(3,4-dihydroxyphenyl)prop-2-enoyl]oxy-2-hydroxybutanedioic acid | 8.39052E-11 | 8.64745E-09 | 1.804747308 | TRUE |
| Gambiriin A1 | 1.05057E-10 | 8.64745E-09 | 1.79243994 | TRUE |
| Casuarinin | 1.4212E-11 | 2.88503E-09 | 1.789933008 | TRUE |
| Shinflavanone | 4.28632E-09 | 1.08765E-07 | 1.742595843 | TRUE |
| 1-O-p-Coumaroyl-β-D-glucose | 8.24047E-09 | 1.85868E-07 | 1.729945887 | TRUE |
| (3R,4S)-4,6,8-Trihydroxy-7-methoxy-3-methyl-3,4-dihydro-1H-isochromen-1-one | 6.25446E-10 | 2.30847E-08 | 1.688423025 | TRUE |
| Gentisate aldehyde | 7.29561E-09 | 1.74236E-07 | 1.628814375 | TRUE |
| Casuarictin | 1.06805E-08 | 2.28226E-07 | 1.615793179 | TRUE |
| trans-2,3-Dihydroxycinnamate | 2.66129E-07 | 3.48543E-06 | 1.571420844 | TRUE |
| Kaempferol -3-glucoside | 5.19336E-07 | 6.38941E-06 | 1.532455381 | TRUE |
| Lyoniresinol 9'-sulfate | 1.47582E-05 | 0.000101557 | 1.500186045 | TRUE |
| 8-Hydroxy-6,7-dimethoxy-2H-chromen-2-one | 6.61437E-08 | 1.16758E-06 | 1.478388496 | TRUE |
| 1-Caffeoyl-β-D-glucose | 1.0472E-06 | 1.03699E-05 | 1.476330005 | TRUE |
| [(1S,21S,22R,23R)-6,7,8,11,12,13,22,23-Octahydroxy-3,16-dioxo-2,17,20-trioxatetracyclo[17.3.1.04,9.010,15]tricosa-4,6,8,10,12,14-hexaen-21-yl]3,4,5-trihydroxybenzoate | 1.71271E-09 | 4.96686E-08 | 1.458948321 | TRUE |
| [4a-Hydroxy-7-(hydroxymethyl)-1-[3,4,5-trihydroxy-6-(hydroxymethyl)oxan-2-yl]oxy-5,7a-dihydro-1H-cyclopenta[c]pyran-5-yl] 4-hydroxybenzoate | 3.28734E-10 | 1.48296E-08 | 1.447178811 | TRUE |
| Vescalagin | 1.55563E-09 | 4.96686E-08 | 1.444009539 | TRUE |
| Myricetin 3-(2'',3''-digalloylrhamnoside) | 2.39916E-07 | 3.35882E-06 | 1.44361006 | TRUE |
| Prunin | 2.29771E-09 | 6.21914E-08 | 1.44143917 | TRUE |
| Galloylglycerol | 4.93125E-06 | 4.25976E-05 | 1.431891633 | TRUE |
| [(2R,3R,4S,5R,6S)-2-(Hydroxymethyl)-4,5,6-tris[(3,4,5-trihydroxybenzoyl)oxy]oxan-3-yl] 3,4,5-trihydroxybenzoate | 4.07723E-08 | 7.52435E-07 | 1.429214587 | TRUE |
| Quercetin-3-O-α-d-arabinofuranoside | 5.84056E-07 | 6.77504E-06 | 1.423530116 | TRUE |
| 2,3-Dihydroxybenzoic Acid | 1.46025E-06 | 1.37875E-05 | 1.415622662 | TRUE |
| Quercetin 3-(2'',6''-digalloylgalactoside) | 2.85977E-05 | 0.000163531 | 1.409319475 | TRUE |
| Castacrenin A | 2.78049E-08 | 5.6444E-07 | 1.403232315 | TRUE |
| α-(1,2-Dihydroxyethyl)-1,2,3,4-tetrahydro-7-hydroxy-9-methoxy-3,4-dioxocyclopenta[c][1]benzopyran-6-acetaldehyde | 3.80371E-10 | 1.54431E-08 | 1.400267392 | TRUE |
| (2S,3S)-3,5,7-Trihydroxy-2-[4-hydroxy-3-[(2S,3R,4S,5S,6R)-3,4,5-trihydroxy-6-(hydroxymethyl)oxan-2-yl]oxyphenyl]-2,3-dihydrochromen-4-one | 0.00137048 | 0.003918415 | 1.392251982 | TRUE |
| Dihydrokaempferol | 0.001678296 | 0.004635294 | 1.385193999 | TRUE |
| 3-(1-Acetoxy-3-methylbutyl)-6-{2-hydroxy-4-methyl-6-[(1E)-3-oxo-1-buten-1-yl]phenoxy}-2-methoxybenzoic acid | 2.70105E-05 | 0.000161269 | 1.383108266 | TRUE |
| 3-(2,4-Dihydroxyphenyl)-5,7-dihydroxy-6,8-bis(3-methylbut-2-enyl)-2,3-dihydrochromen-4-one | 1.67638E-05 | 0.000113435 | 1.363227902 | TRUE |
| [3-Hydroxy-4-[(2S,3R,4S,5S,6R)-3,4,5-trihydroxy-6-(hydroxymethyl)oxan-2-yl]oxyphenyl]methyl 3,4-dihydroxybenzoate | 0.000142794 | 0.000644159 | 1.357311199 | TRUE |
| Edulisin I | 5.46899E-07 | 6.53062E-06 | 1.35117697 | TRUE |
| 2-O-Caffeoyltartronic acid | 0.026613462 | 0.050095898 | 1.350340769 | FALSE |
| 5-Hydroxy-2-(4-hydroxyphenyl)-7-[(2S,3R,4S,5S,6R)-3,4,5-trihydroxy-6-(hydroxymethyl)oxan-2-yl]oxy-2,3-dihydrochromen-4-one | 3.79319E-08 | 7.33351E-07 | 1.34964673 | TRUE |
| 7-Hydroxy-5-methoxy-6,8-dimethylflavanone | 7.06859E-05 | 0.000354302 | 1.345137192 | TRUE |
| Granatin A | 6.47757E-07 | 7.1682E-06 | 1.341679584 | TRUE |
| 2-Hydroxy-3-(4-hydroxyphenyl)propenoate | 8.08655E-06 | 6.31373E-05 | 1.332114023 | TRUE |
| Swertiajaponin | 0.000574553 | 0.001943904 | 1.329866631 | TRUE |
| 1-Hydroxy-4-[(2S,3R,4S,5S,6R)-3,4,5-trihydroxy-6-(hydroxymethyl)oxan-2-yl]oxynaphthalene-2-carboxylic acid | 2.57967E-05 | 0.000156321 | 1.32691636 | TRUE |
| (-)-Epigallocatechin 3-cinnamate | 1.74718E-10 | 1.01337E-08 | 1.317716084 | TRUE |
| Catechin 7-arabinofuranoside | 0.400398406 | 0.461823162 | 1.314084636 | FALSE |
| Methyl gallate | 0.000189519 | 0.000793243 | 1.313790065 | TRUE |
| Okanin 4'-(3'',4'',6''-triacetylglucoside) | 4.29017E-07 | 5.44316E-06 | 1.300690015 | TRUE |
| Procyanidin B5 | 0.001868195 | 0.00505658 | 1.300315418 | TRUE |
| Spinacetin 3-glucoside | 0.000304907 | 0.001135707 | 1.295406535 | TRUE |
| 1-O,6-O-Digalloyl-β-D-glucose | 0.000464714 | 0.001653891 | 1.290496451 | TRUE |
| 1,4-Di-O-galloyl-3,6-(R)-hexahydroxydiphenoyl-b-D-glucopyranose | 0.000434402 | 0.001560771 | 1.288020362 | TRUE |
| Radicinin | 6.70915E-07 | 7.1682E-06 | 1.287326682 | TRUE |
| Melledonol | 6.49482E-05 | 0.000333785 | 1.278460662 | TRUE |
| Kaempferide 3-rhamnoside | 0.000523796 | 0.001817616 | 1.275712021 | TRUE |
| Apigenin 7-(6''-ethylglucuronide) | 7.69185E-05 | 0.00038084 | 1.272873549 | TRUE |
| Methyl 3,4-dihydroxy-5-prenylbenzoate 3-glucoside | 0.00213758 | 0.005747399 | 1.264129249 | TRUE |
| Sinapyl alcohol | 0.008174457 | 0.01864511 | 1.263651317 | TRUE |
| 3,4-Dihydroxyphenylacetic acid | 8.47566E-05 | 0.000414593 | 1.262318671 | TRUE |
| 5,2'-Dihydroxy-3,6,7,8,4',5'-hexamethoxyflavone | 0.546541078 | 0.590148079 | 1.262316239 | FALSE |
| 3-Ethenyl-4-[2-(3,4,5-trihydroxybenzoyl)oxyethyl]-2-[3,4,5-trihydroxy-6-(hydroxymethyl)oxan-2-yl]oxy-3,4-dihydro-2H-pyran-5-carboxylic acid | 2.5475E-07 | 3.44761E-06 | 1.261087886 | TRUE |
| Flaccidine | 7.85417E-06 | 6.25254E-05 | 1.256244559 | TRUE |
| Rhamnetin | 7.3854E-06 | 6.11933E-05 | 1.25503431 | TRUE |
| Delphinidin | 1.71818E-05 | 0.000114358 | 1.254052322 | TRUE |
| 2-({6-O-[(2E)-3-(4-Hydroxyphenyl)-2-propenoyl]-D-glucopyranosyl}oxy)-3-phenylacrylic acid | 2.76651E-05 | 0.000161324 | 1.252946184 | TRUE |
| 2'-C-Methylmyricetin 3-rhamnoside 5'-gallate | 0.002498625 | 0.00667396 | 1.251686033 | TRUE |
| 2-O-Caffeoylarbutin | 0.000304131 | 0.001135707 | 1.246919389 | TRUE |
| [(2S,3R,4S,5S,6R)-2-[2-(3,4-Dihydroxyphenyl)-5,7-dihydroxy-4-oxo-chromen-3-yl]oxy-4,5-dihydroxy-6-(hydroxymethyl)tetrahydropyran-3-yl] 3,4,5-trihydroxybenzoate | 0.001067926 | 0.003235656 | 1.246802692 | TRUE |
| Secoisolariciresinol | 0.0819786 | 0.124994661 | 1.24047388 | FALSE |
| 3-p-Coumaroyl-1,5-quinolactone | 0.438066841 | 0.489959056 | 1.236670849 | FALSE |
| Quercetin 7-O-rhamnoside | 1.09083E-06 | 1.05447E-05 | 1.236624077 | TRUE |
| 7-O-Galloylcatechin | 0.000721311 | 0.002270172 | 1.232353273 | TRUE |
| 5,4'-Dihydroxy-6,7,8,3'-tetramethoxyflavone 4'-galactoside | 0.000166521 | 0.000726961 | 1.231873994 | TRUE |
| 5,7-Dihydroxy-3',4'-dimethoxy-8-(3-hydroxy-3-methylbutyl)-isoflavone 7-glucoside | 0.000669413 | 0.002174252 | 1.228069332 | TRUE |
| (-)-Naringenin 7-O-β-D-glucoside | 0.000246705 | 0.000991706 | 1.227033121 | TRUE |
| Naringerin | 0.000256986 | 0.001003233 | 1.226747931 | TRUE |
| (-)-Catechin 3-O-gallate | 1.37224E-05 | 9.66621E-05 | 1.226460793 | TRUE |
| Heterophylliin A | 0.000691541 | 0.002195863 | 1.221404488 | TRUE |
| Isorhamnetin | 6.53338E-07 | 7.1682E-06 | 1.215784498 | TRUE |
| Daidzein | 2.06761E-07 | 3.10908E-06 | 1.212428183 | TRUE |
| Miraxanthin-V | 1.77208E-07 | 2.76718E-06 | 1.211603162 | TRUE |
| Robinetinidol 3-O-gallate | 3.44513E-05 | 0.000189016 | 1.211347431 | TRUE |
| Sanguiin H4 | 0.00135146 | 0.003891438 | 1.206855638 | TRUE |
| Phloretin | 1.63379E-06 | 1.47404E-05 | 1.194465733 | TRUE |
| Epigallocatechin 3-O-vanillate | 0.000536975 | 0.001847557 | 1.192727137 | TRUE |
| Naringenin | 0.000187928 | 0.000793243 | 1.190283533 | TRUE |
| Frangulin B | 0.088016084 | 0.131600757 | 1.188544621 | FALSE |
| 3-p-Coumaroylquinic acid | 0.000107835 | 0.000521203 | 1.188127585 | TRUE |
| Garbanzol | 0.001481942 | 0.004207472 | 1.182394407 | TRUE |
| Iridin | 1.38089E-05 | 9.66621E-05 | 1.181702138 | TRUE |
| Garcimangosone D | 0.792954978 | 0.806866469 | 1.181701128 | FALSE |
| 4'-Prenyloxyresveratrol | 0.008376301 | 0.01878883 | 1.181184726 | TRUE |
| Kanzonol Z | 7.04477E-05 | 0.000354302 | 1.181027585 | TRUE |
| Isochinomin | 0.006211624 | 0.014748066 | 1.177154311 | TRUE |
| 4-O-Methylgallic acid | 0.00013758 | 0.000637377 | 1.174790088 | TRUE |
| Resveratrol | 2.78145E-05 | 0.000161324 | 1.171809114 | TRUE |
| (-)-Epicatechin | 0.002806173 | 0.007398092 | 1.170982636 | TRUE |
| 2,3,4-Trihydroxybenzoic acid | 0.000349204 | 0.001277269 | 1.170245 | TRUE |
| 1,5,8-Trihydroxy-4-methoxy-3-[(2S,3R,4S,5S,6R)-3,4,5-trihydroxy-6-(hydroxymethyl)oxan-2-yl]oxyxanthen-9-one | 0.277361498 | 0.341238691 | 1.168868244 | FALSE |
| 3'-Methoxyfukiic acid | 1.52951E-07 | 2.58743E-06 | 1.16789622 | TRUE |
| 3'-(2'',3''-Digalloylglucosyl)-phloroacetophenone | 2.35975E-05 | 0.000147872 | 1.166589982 | TRUE |
| Sanguiin H11 | 0.002866582 | 0.007508596 | 1.15538032 | TRUE |
| 4-Hydroxy-3,5-dimethoxybenzaldehyde | 0.000184093 | 0.000786757 | 1.15446395 | TRUE |
| Santin | 6.73328E-06 | 5.69523E-05 | 1.153549103 | TRUE |
| (S)-2,3-Dihydro-7-hydroxy-2-methyl-4-oxo-4H-1-benzopyran-5-acetic acid | 0.00068868 | 0.002195863 | 1.150333002 | TRUE |
| 1a,2,3,4,5b,11,11a,11b-Octahydro-10,11,11a-trihydroxy-3-methyl-benz[3,4]anthra[1,2-b]oxirene-5,6-dione | 0.00795328 | 0.018243117 | 1.148369505 | TRUE |
| Dicumarol | 0.004846566 | 0.012146333 | 1.147769083 | TRUE |
| Chrysanthemin | 3.36296E-05 | 0.000187036 | 1.147365893 | TRUE |
| Carnosol | 0.001853905 | 0.00505158 | 1.142216057 | TRUE |
| (+)-Catechin | 0.00060506 | 0.002013559 | 1.141943928 | TRUE |
| Poliothyrsoside; Nigracin | 6.47355E-05 | 0.000333785 | 1.140639812 | TRUE |
| 8-C-Glucosylnaringenin | 0.001322352 | 0.00385461 | 1.131212977 | TRUE |
| 6-Hydroxyluteolin | 0.01093457 | 0.023489076 | 1.116943851 | TRUE |
| Arbutin | 0.194540557 | 0.253966129 | 1.116383097 | FALSE |
| Eriodictin | 7.16809E-07 | 7.46216E-06 | 1.114978895 | TRUE |
| Salidroside | 0.269726912 | 0.332854487 | 1.113854404 | FALSE |
| trans-p-Hydroxycinnamic acid | 1.90809E-05 | 0.000122965 | 1.110034572 | TRUE |
| Phlorizin chalcone | 0.08642405 | 0.129956164 | 1.109435829 | FALSE |
| ent-Epicatechin-(4α->8)-ent-epicatechin 3-gallate | 0.000309863 | 0.001143678 | 1.107275916 | TRUE |
| Cyclopenta[c]pyran-4-carboxylic acid, 1,4a,5,7a-tetrahydro-7-(hydroxymethyl)-1-[[6-O-[(2E)-3-(4-hydroxyphenyl)-1-oxo-2-propen-1-yl]-β-D-glucopyranosyl]oxy]-, methyl ester | 0.000147515 | 0.000658146 | 1.102931279 | TRUE |
| 3,5-Di-O-galloyl-4-O-digalloylquinic acid | 0.000543756 | 0.001855167 | 1.102307617 | TRUE |
| Malvidin 3-galactoside | 1.63289E-06 | 1.47404E-05 | 1.098183364 | TRUE |
| Purpurin | 0.010323219 | 0.022412978 | 1.096997009 | TRUE |
| 4'-O-Prenylalpinumisoflavone | 0.000158224 | 0.00069825 | 1.094731679 | TRUE |
| 4-(3,4-Dihydroxyphenyl)-2,3-dihydro-2,3-dihydroxy-1H-phenalen-1-one | 0.000131609 | 0.000621319 | 1.094583498 | TRUE |
| Kuwanon E | 0.005732918 | 0.013937513 | 1.094195121 | TRUE |
| Pilosin | 2.35592E-07 | 3.35882E-06 | 1.093116023 | TRUE |
| 1,5,8-Trihydroxy-4-methoxy-9-oxo-9H-xanthen-3-yl-D-glucopyranoside | 0.006186421 | 0.014748066 | 1.091158883 | TRUE |
| Vescalin | 0.003991137 | 0.010321028 | 1.090719582 | TRUE |
| Limocitrol | 0.201416513 | 0.260430269 | 1.090517657 | FALSE |
| 3,4,5-Trihydroxy-6-[5-hydroxy-2-(4-hydroxy-3-methoxyphenyl)-3-methoxy-4-oxochromen-7-yl]oxyoxane-2-carboxylic acid | 4.87695E-05 | 0.000260532 | 1.089995458 | TRUE |
| 4-Acetyl-3-hydroxy-5-methylphenyl β-D-glucopyranoside | 1.2316E-05 | 9.09145E-05 | 1.089909688 | TRUE |
| 4'-Hydroxy-3',5,6,7,8-pentamethoxyflavone | 0.00155631 | 0.004373941 | 1.089572234 | TRUE |
| Isoliquiritigenin | 0.026125417 | 0.049565043 | 1.089220378 | TRUE |
| 2-(3,4-Dihydroxyphenyl)-5,7-dihydroxy-3-[(3,4,5-trihydroxy-6-methyloxan-2-yl)oxy]-4H-chromen-4-one | 0.000198565 | 0.000819427 | 1.089100829 | TRUE |
| 2-Methoxyresorcinol | 1.34931E-05 | 9.66621E-05 | 1.088903071 | TRUE |
| 10-Hydroxycamptothecin | 0.033882382 | 0.061411818 | 1.082049024 | FALSE |
| (2R,3S)-2-[(3,4-Dihydroxyphenyl)methyl]-2-hydroxy-3-[(E)-3-(4-hydroxy-3-methoxyphenyl)prop-2-enoyl]oxybutanedioic acid | 0.686011684 | 0.713831757 | 1.081802466 | FALSE |
| Dl-4-Hydroxy-3-methoxymandelic acid | 0.000139721 | 0.000637377 | 1.080306404 | TRUE |
| Epicatechin-(4β->8)-gallocatechin | 0.00013941 | 0.000637377 | 1.078176655 | TRUE |
| Dihydroisorhamnetin | 0.000256029 | 0.001003233 | 1.073521709 | TRUE |
| Demethoxysudachitin | 0.62415516 | 0.659914049 | 1.064500569 | FALSE |
| Curcumin monoglucoside | 1.1945E-05 | 8.98086E-05 | 1.064244282 | TRUE |
| Castanin | 0.00012881 | 0.000615257 | 1.063633771 | TRUE |
| Glucocaffeic acid | 0.061060146 | 0.101185384 | 1.062519758 | FALSE |
| Quercetin | 0.046778684 | 0.080135636 | 1.062345144 | FALSE |
| 3-{(E)-2-[4-(β-D-Glucopyranosyloxy)-2-hydroxyphenyl]vinyl}-5-hydroxyphenyl β-D-glucopyranoside | 1.09505E-05 | 8.38853E-05 | 1.061773613 | TRUE |
| (+-)-Dalbergioidin | 0.004997094 | 0.01229588 | 1.059741297 | TRUE |
| 2,4,6-Phenanthrenetriol 2-O-b-D-glucoside | 0.009913069 | 0.021755168 | 1.058930256 | TRUE |
| Procyanidin B4 | 0.159602951 | 0.215995994 | 1.057922027 | FALSE |
| α-D-Glucopyranoside, 3,6-bis-O-[(2E)-3-(4-hydroxy-3-methoxyphenyl)-1-oxo-2-propen-1-yl]-β-D-fructofuranosyl | 0.421723876 | 0.476935637 | 1.056646743 | FALSE |
| Loquatoside | 0.002636214 | 0.006995443 | 1.056642948 | TRUE |
| 4'-O-Galloylsucrose | 1.82614E-05 | 0.000119583 | 1.056223729 | TRUE |
| Enterolactone | 0.008370691 | 0.01878883 | 1.055820122 | TRUE |
| 2,3-Dihydro-2,3-dihydroxy-4-(4-methoxyphenyl)-1H-phenalen-1-one | 0.161781368 | 0.216963759 | 1.054330873 | FALSE |
| 2-O-Galloylsucrose | 0.067852977 | 0.108032583 | 1.051809304 | FALSE |
| Gallomyricitrin | 0.075477538 | 0.116961376 | 1.049983442 | FALSE |
| Quercetin 3-D-glucoside | 0.001207049 | 0.003577093 | 1.049485292 | TRUE |
| Delphinidin 3-O-glucoside | 0.00079219 | 0.002455185 | 1.046551558 | TRUE |
| 5-Hydroxy-3,3',4',7,8-pentamethoxyflavone | 0.020260041 | 0.039546042 | 1.045926105 | TRUE |
| Ellagic acid | 0.001206984 | 0.003577093 | 1.044229341 | TRUE |
| Heterophylliin E | 0.00061403 | 0.002026799 | 1.042554764 | TRUE |
| 2,5-Dihydroxycinnamic acid methyl ester | 0.012771079 | 0.026454377 | 1.036715052 | TRUE |
| Aloesin | 0.281672017 | 0.345494981 | 1.033652586 | FALSE |
| Biflorin | 0.022912095 | 0.044086779 | 1.033559231 | TRUE |
| Irisolidone 7-O-glucuronide | 0.049792021 | 0.084231502 | 1.026122193 | FALSE |
| Quercetin 3-(6''-p-hydroxybenzoylgalactoside) | 5.84891E-05 | 0.000308397 | 1.014911948 | TRUE |
| Phloroglucinol carboxylic acid | 0.000519301 | 0.001817554 | 1.014786582 | TRUE |
| (-)-Epicatechin-3'-O-glucuronide | 0.000252273 | 0.001003233 | 1.01184323 | TRUE |
| 2,4a,6-Trihydroxy-8-isopropyl-1,1-dimethyl-2,3,4,4a,5,10,11,11a-octahydro-1H-dibenzo[a,d][7]annulen-7-yl β-D-glucopyranoside | 0.00178558 | 0.004898281 | 1.007918221 | TRUE |
| α-Hydrojuglone 4-O-b-D-glucoside | 0.055879898 | 0.093694787 | 1.007497453 | FALSE |
| Sericetin | 0.005900623 | 0.014175461 | 1.000374505 | TRUE |
| Dihydrogenistein | 0.056078407 | 0.093694787 | 1.000214316 | FALSE |
| Quercitrin | 0.616296371 | 0.656735766 | 0.99997704 | FALSE |
| Orotinichalcone | 0.12849087 | 0.179887218 | 0.997391325 | FALSE |
| Procyanidin B2 | 0.000468467 | 0.001653891 | 0.993833755 | TRUE |
| 2-Hydroxycyclohexyl 2-O-[(2E)-3-(3,4-Dihydroxyphenyl)-2-propenoyl]-β-D-glucopyranoside | 1.70512E-07 | 2.76718E-06 | 0.993382545 | TRUE |
| 6''-O-Acetyldaidzin | 0.028965309 | 0.053944567 | 0.98680291 | FALSE |
| Pimentol | 0.093421352 | 0.136928047 | 0.983047912 | FALSE |
| Hispidol | 0.212796532 | 0.27340314 | 0.982805769 | FALSE |
| 4,6-Dihydroxy-3-(1-hydroxyethyl)-5-methoxy-3H-2-benzofuran-1-one | 2.12811E-06 | 1.87829E-05 | 0.979010247 | TRUE |
| 6''-O-Acetylholocalin | 0.004598472 | 0.011596147 | 0.978568964 | TRUE |
| 4'-O-methyl-(-)-epicatechin-7-O-β-glucuronide | 0.091842451 | 0.13608772 | 0.978110474 | FALSE |
| 6'-O-Galloylsucrose | 0.001138093 | 0.00342271 | 0.97766482 | TRUE |
| 3,4-Dihydroxy-5-(3,4,5-trihydroxybenzoyloxy)benzoic acid;Digallic acid | 0.265260887 | 0.329302446 | 0.97419879 | FALSE |
| Punicacortein A | 0.00405708 | 0.010425156 | 0.974044323 | TRUE |
| 5-Hydroxy-3-[4-hydroxy-2-[(2S,3R,4S,5S,6R)-3,4,5-trihydroxy-6-(hydroxymethyl)oxan-2-yl]oxyphenyl]-7-methoxychromen-4-one | 0.089954027 | 0.133777783 | 0.971404831 | FALSE |
| Cajanone | 0.001270356 | 0.003737423 | 0.970822113 | TRUE |
| 1,6-Digalloyl-β-D-glucopyranose | 0.133053124 | 0.184998522 | 0.968858327 | FALSE |
| Uncinatone | 0.000292539 | 0.001110009 | 0.966539279 | TRUE |
| Isoorientin | 0.000580688 | 0.001948423 | 0.966247901 | TRUE |
| Quercetin 3-(2''-galloyl-α-L-arabinopyranoside) | 0.000666591 | 0.002174252 | 0.965559152 | TRUE |
| Taxodione | 0.008740175 | 0.019497313 | 0.965446484 | TRUE |
| ε-Viniferin | 0.000271698 | 0.001040654 | 0.963997561 | TRUE |
| 9-Hydroxycalabaxanthone | 0.012272738 | 0.025552471 | 0.963801799 | TRUE |
| Marein | 0.037840879 | 0.06738332 | 0.961658267 | FALSE |
| Viscidulin I | 0.000209863 | 0.000852043 | 0.9610334 | TRUE |
| Hydrojuglone glucoside | 0.334493236 | 0.401787733 | 0.958489863 | FALSE |
| (3-hydroxy-4-{[(2S,3R,4S,5S,6R)-3,4,5-trihydroxy-6-(hydroxymethyl)oxan-2-yl]oxy}phenyl)methyl 4-hydroxy-3,5-dimethoxybenzoate | 0.000980705 | 0.00299373 | 0.957911635 | TRUE |
| Caffeic acid | 0.116985047 | 0.167239188 | 0.957241113 | FALSE |
| Maesopsin 6-glucoside | 0.053411741 | 0.089979946 | 0.948564376 | FALSE |
| 1-O-Sinapoylglucose | 0.001329176 | 0.00385461 | 0.938938715 | TRUE |
| Corilagin | 0.042265801 | 0.073647704 | 0.933293976 | FALSE |
| 4-Hydroxy-5-(dihydroxyphenyl)-valeric acid-O-methyl-O-sulphate | 0.008350082 | 0.01878883 | 0.932364945 | TRUE |
| Lonchocarpenin | 0.088166025 | 0.131600757 | 0.928985611 | FALSE |
| Vitisidin A | 0.06185849 | 0.101678328 | 0.928936498 | FALSE |
| Tectoridin | 0.961390814 | 0.961390814 | 0.927130832 | FALSE |
| 6-Methoxyluteolin | 0.018026207 | 0.035875686 | 0.922838661 | TRUE |
| 2'-O-Methylisoliquiritigenin | 0.004311192 | 0.010939649 | 0.922075498 | TRUE |
| Garcimangosone B | 0.007458379 | 0.017402883 | 0.921822598 | TRUE |
| 3-(4-Hydroxyphenyl)-3-oxopropyl-β-D-glucopyranoside | 0.001649696 | 0.00458751 | 0.921552657 | TRUE |
| Kaempferol 7-O-β-D-glucopyranoside | 0.000261182 | 0.001009905 | 0.919354382 | TRUE |
| Fukiic acid | 0.037076896 | 0.066607166 | 0.918381812 | FALSE |
| (2R,3S)-2,3-Dihydro-5,5a(2),7,7a(2)-tetrahydroxy-2-(4-hydroxyphenyl)[3,8a(2)-bi-4H-1-benzopyran]-4,4a(2)-dione | 0.07233732 | 0.113393637 | 0.917284625 | FALSE |
| Sesamolinol | 0.006389312 | 0.014994571 | 0.917038639 | TRUE |
| Bergenin | 0.004895832 | 0.012194526 | 0.916135095 | TRUE |
| 2-Galloyl-D-glucose | 0.006341888 | 0.014969806 | 0.915921055 | TRUE |
| Occidentoside | 0.07288097 | 0.113806438 | 0.915900554 | FALSE |
| Palmidin A | 0.00784047 | 0.018086538 | 0.914958338 | TRUE |
| Phloretin 2'-O-glucuronide | 0.000360972 | 0.001308523 | 0.914695549 | TRUE |
| 3-O-Caffeoyl-4-O-methylquinic acid | 0.098267531 | 0.14299863 | 0.914104406 | FALSE |
| Bracteatin 6-O-glucoside | 0.014432541 | 0.029445286 | 0.914015063 | TRUE |
| Sanguisorbic acid dilactone | 0.04015268 | 0.070267189 | 0.913101427 | FALSE |
| 1,2,3-Tris-O-(3,4,5-trihydroxybenzoyl)-D-glucopyranose | 0.002928899 | 0.007622648 | 0.911413508 | TRUE |
| 6-O-p-Coumaroyl-1,2-di-O-galloyl-b-D-glucopyranose | 0.01648293 | 0.033460348 | 0.910694845 | TRUE |
| Afzelechin 7-apioside | 0.048245643 | 0.081957034 | 0.908544357 | FALSE |
| Sanguiin H1 | 0.198155924 | 0.257856747 | 0.907047895 | FALSE |
| Glabridin | 2.43966E-05 | 0.000150076 | 0.903851662 | TRUE |
| Xanthoxylin | 0.01762867 | 0.035538642 | 0.901954726 | TRUE |
| Pyrogallin | 0.007597959 | 0.017627266 | 0.901882419 | TRUE |
| (7'R,8'R)-4,7'-Epoxy-3',5-dimethoxy-4',9,9'-lignanetriol 9'-glucoside | 0.030429177 | 0.056155663 | 0.901661003 | FALSE |
| Licoagroside A | 0.001562122 | 0.004373941 | 0.899927564 | TRUE |
| 2-[3,4-bis[[(2S,3R,4S,5S,6R)-3,4,5-Trihydroxy-6-(hydroxymethyl)oxan-2-yl]oxy]phenyl]-5,7-dihydroxychromen-4-one | 0.043830899 | 0.075724873 | 0.894867534 | FALSE |
| Isolariciresinol 4'-O-β-D-glucoside | 0.039344345 | 0.069314114 | 0.891899987 | FALSE |
| Irilone | 0.081588319 | 0.124994661 | 0.891778802 | FALSE |
| Auriculoside | 0.223700186 | 0.282056757 | 0.890783182 | FALSE |
| 2-(3,4-Dihydroxyphenyl)-5,7-dihydroxy-6-[3,4,5-trihydroxy-6-(hydroxymethyl)oxan-2-yl]-4H-chromen-4-one | 0.01943469 | 0.038118281 | 0.889181071 | TRUE |
| Eujambin | 0.082581667 | 0.125035786 | 0.887194311 | FALSE |
| 5,8-Dihydroxy-4-oxo-1,2,3,4-tetrahydro-1-naphthalenyl 6-O-(3,4,5-trihydroxybenzoyl)-β-D-glucopyranoside | 0.029460484 | 0.05461624 | 0.885644711 | FALSE |
| Procyanidin B7 | 7.64631E-06 | 6.20881E-05 | 0.883522576 | TRUE |
| Isomucronulator 7-O-glucoside | 0.014321383 | 0.029445286 | 0.882635361 | TRUE |
| 3'-(2'',6''-Digalloylglucosyl)-phloroacetophenone | 0.01188328 | 0.024869133 | 0.880344816 | TRUE |
| sec-o-Glucosylhamaudol | 0.024497198 | 0.046914445 | 0.880057772 | TRUE |
| 3-(4-Hydroxy-3-methoxy-phenyl)prop-2-enoic acid | 0.009841339 | 0.021715128 | 0.879543164 | TRUE |
| Nanafrocin | 0.039437341 | 0.069314114 | 0.874646191 | FALSE |
| Homomangiferin | 0.000199811 | 0.000819427 | 0.874067101 | TRUE |
| Sanguiin H7 | 0.124784938 | 0.175303407 | 0.868699341 | FALSE |
| 4-p-Coumaroylquinic acid | 0.004975012 | 0.01229588 | 0.86782575 | TRUE |
| 1,2'-Di-O-galloylhamamelofuranose | 0.172753734 | 0.228462592 | 0.86697183 | FALSE |
| 1,2,6-Trigalloylglucose | 0.540141496 | 0.586356812 | 0.864864255 | FALSE |
| 1,4,6-Trigalloyl-β-D-glucopyranose | 0.071215737 | 0.112504238 | 0.864534033 | FALSE |
| Kaempferol 3-(2''-acetylrhamnoside) | 0.01168681 | 0.024584688 | 0.863816021 | TRUE |
| Feruloyl-2-hydroxyputrescine | 0.943467976 | 0.945797527 | 0.8630075 | FALSE |
| Quercetin-3-glucoside | 0.00515696 | 0.012612806 | 0.862067264 | TRUE |
| N-[4'-hydroxy-(E)-cinnamoyl]-L-aspartic acid | 0.037803475 | 0.06738332 | 0.861229422 | FALSE |
| Esculetin | 0.065579653 | 0.105238495 | 0.860126579 | FALSE |
| Procyanidin B6 | 1.59266E-09 | 4.96686E-08 | 0.857885092 | TRUE |
| 3,4,8,9,10-Pentahydroxy-6-oxo-6H-benzo[c]chromene-1-carboxylate | 0.0308354 | 0.056610559 | 0.857104565 | FALSE |
| Kaempferol 3-(6-acetylgalactoside) | 0.164319365 | 0.218733319 | 0.856399735 | FALSE |
| 3-O-Acetylpinobanksin | 1.06496E-10 | 8.64745E-09 | 0.855972482 | TRUE |
| Cyclohexanecarboxylic acid, 1,3,4-trihydroxy-5-[[(2E)-3-(4-hydroxyphenyl)-1-oxo-2-propen-1-yl]oxy]-, (1R,3R,4S,5R)- | 0.376694333 | 0.440743225 | 0.854549917 | FALSE |
| Tangeretin | 0.162645735 | 0.217217659 | 0.849773999 | FALSE |
| Sinapic acid | 0.64303985 | 0.674610282 | 0.843704805 | FALSE |
| 4-Methyumbelliferone | 0.161921229 | 0.216963759 | 0.843274697 | FALSE |
| Isobiflorin 6''-gallate | 0.384195488 | 0.446943748 | 0.841639032 | FALSE |
| (-)-Gallocatechin | 0.011534726 | 0.024518842 | 0.840887901 | TRUE |
| (-)-trans-3,4-Dihydro-4,8-dihydroxy-3-methyl-1H-2-benzopyran-1-one | 0.337965547 | 0.403570624 | 0.838395679 | FALSE |
| (7'R,8'R)-4,7'-Epoxy-3'-methoxy-4',5,9,9'-lignanetetrol 9'-glucoside | 0.121158785 | 0.172217467 | 0.838023177 | FALSE |
| 4'-Hydroxyanigorootin | 0.510927757 | 0.557625455 | 0.833868708 | FALSE |
| Chlorogenic acid | 0.000171141 | 0.000739185 | 0.831233161 | TRUE |
| Hesperetin 5-O-glucoside | 0.000692292 | 0.002195863 | 0.827528983 | TRUE |
| Punigluconin | 0.045206173 | 0.077769942 | 0.825320529 | FALSE |
| Episyringaresinol 4'-O-β-D-glucopyranoside | 0.216150511 | 0.275965747 | 0.825044314 | FALSE |
| Catechin-4-ol 3'-methyl ether 3-O-α-L-rhamnopyranoside | 0.027902965 | 0.052205547 | 0.820638292 | FALSE |
| (+)-Chebulic acid | 0.033794648 | 0.061411818 | 0.819518588 | FALSE |
| 1,2',3,5-Tetra-O-galloylhamamelofuranose | 0.064656728 | 0.10512584 | 0.818850156 | FALSE |
| Salicylic acid | 0.000790229 | 0.002455185 | 0.818284382 | TRUE |
| [(2R,3R,4S,5R,6S)-3-Hydroxy-4,5,6-tris[(3,4,5-trihydroxybenzoyl)oxy]oxan-2-yl]methyl 3,4,5-trihydroxybenzoate | 0.362748671 | 0.427470033 | 0.817774961 | FALSE |
| Petunidin 3-O-glucoside | 0.01784406 | 0.035688121 | 0.816400553 | TRUE |
| Salicin | 0.014362826 | 0.029445286 | 0.814688423 | TRUE |
| Avicularin | 0.339680969 | 0.404429541 | 0.813807817 | FALSE |
| 2,4-Dihydroxybenzoic Acid | 0.011665399 | 0.024584688 | 0.813261768 | TRUE |
| Eugeniin | 2.36742E-05 | 0.000147872 | 0.804582332 | TRUE |
| 1,2,3,6-Tetragalloyl-β-D-glucopyranose | 7.87748E-07 | 7.99564E-06 | 0.802474657 | TRUE |
| Methyl 2,4,6-trihydroxybenzoate | 0.065071373 | 0.10512584 | 0.802274027 | FALSE |
| Epicatechin 5-O-β-D-glucopyranoside-3-benzoate | 0.217888114 | 0.276445545 | 0.799158954 | FALSE |
| 2-[4-[3-[3,4-Dihydroxy-4-(hydroxymethyl)oxolan-2-yl]oxy-4,5-dihydroxy-6-(hydroxymethyl)oxan-2-yl]oxyphenyl]-7-hydroxy-2,3-dihydrochromen-4-one | 0.043731996 | 0.075724873 | 0.796199383 | FALSE |
| 3-(3,5-Dihydroxyphenyl)-2-propenoic acid | 0.024733556 | 0.047144713 | 0.794727043 | TRUE |
| 6,7,4'-Trihydroxyflavanone | 0.426128273 | 0.479246755 | 0.791347498 | FALSE |
| (+)-Catechin 7-O-β-D-xyloside | 0.019100918 | 0.037645499 | 0.782390032 | TRUE |
| (-)-Epigallocatechin 3-(3-methyl-gallate) | 0.112482859 | 0.161371169 | 0.779282803 | FALSE |
| 2,4',5,7-Tetrahydroxyflavanone | 0.2170134 | 0.276198873 | 0.777700942 | FALSE |
| Orientin 7,3'-dimethyl ether | 0.13463489 | 0.186558927 | 0.776117811 | FALSE |
| 2''-O-Acetylisoorientin | 0.022342425 | 0.043195355 | 0.774727214 | TRUE |
| Eriodictyol 7,3'-dimethyl ether | 0.011497321 | 0.024518842 | 0.772484806 | TRUE |
| ent-Epicatechin-(4α->8)-ent-epicatechin 3'-gallate | 0.000886115 | 0.002725474 | 0.77063151 | TRUE |
| (S)-Multifidol 2-[apiosyl-(1->6)-glucoside] | 0.030954542 | 0.056610559 | 0.770303554 | FALSE |
| 1,7-Bis(4-hydroxyphenyl)-3-heptanyl 2-O-[(2R,3R,4R)-3,4-dihydroxy-4-(hydroxymethyl)tetrahydro-2-furanyl]-6-O-[(2S,3R,4R)-3,4-dihydroxy-4-(hydroxymethyl)tetrahydro-2-furanyl]-D-glucopyranoside | 0.584106287 | 0.62737342 | 0.768323225 | FALSE |
| Neocarthamin | 0.121877739 | 0.172217467 | 0.768239267 | FALSE |
| Castacrenin B | 0.528955902 | 0.575753609 | 0.767694427 | FALSE |
| Quercetagetin 7-(6''-(E)-caffeoylglucoside) | 0.353737886 | 0.419934449 | 0.766542422 | FALSE |
| Isorhoifolin | 0.074748656 | 0.116275686 | 0.764467778 | FALSE |
| Catalposide | 0.503132094 | 0.55155458 | 0.763954453 | FALSE |
| 3'-Glucosyl-2',4',6'-trihydroxyacetophenone | 0.010301776 | 0.022412978 | 0.763517706 | TRUE |
| Phloridzin | 0.443916169 | 0.493780724 | 0.762578527 | FALSE |
| [(2R,3R,4S)-6-Hydroxy-4-(4-hydroxy-3-methoxyphenyl)-3-(hydroxymethyl)-7-methoxy-1,2,3,4-tetrahydro-2-naphthalenyl]methyl β-D-glucopyranoside | 0.092798501 | 0.136928047 | 0.762114625 | FALSE |
| 2-(β-D-Glucopyranosyloxy)benzyl (2E)-3-(3,4-dihydroxyphenyl)acrylate | 0.292736787 | 0.356910317 | 0.762066751 | FALSE |
| Myricetin 7-(6''-galloylglucoside) | 0.034891743 | 0.062960212 | 0.76056834 | FALSE |
| Orientin | 0.122164115 | 0.172217467 | 0.753112649 | FALSE |
| Astilbin | 0.047193854 | 0.080507162 | 0.752508686 | FALSE |
| Dryopteric acid | 0.402175423 | 0.462353741 | 0.7521561 | FALSE |
| 6-Cinnamoyl-1-galloylglucose | 0.060032029 | 0.09988936 | 0.74931246 | FALSE |
| Leucodelphidin | 0.038069164 | 0.067493802 | 0.7489547 | FALSE |
| 1-O-Feruloylglucose | 0.061390272 | 0.101318904 | 0.747520613 | FALSE |
| [(2R,3S,4S,5R,6S)-6-(3,4-Dihydroxybenzoyl)oxy-3,4,5-trihydroxyoxan-2-yl]methyl 3,4-dihydroxybenzoate | 0.0932574 | 0.136928047 | 0.744784916 | FALSE |
| Eriodictyol | 0.167018008 | 0.221599056 | 0.744150419 | FALSE |
| Methyl nogalonate | 0.137231923 | 0.18951075 | 0.743155874 | FALSE |
| (S)-(4-Hydroxy-3-methoxyphenyl)[(3S,4R,5S)-5-(4-hydroxy-3-methoxyphenyl)-4-(hydroxymethyl)tetrahydro-3-furanyl]methyl β-D-glucopyranoside | 0.1569287 | 0.213087131 | 0.735303239 | FALSE |
| 4-Hydroxy-3-methoxycinnamaldehyde | 0.067145006 | 0.107326269 | 0.732600508 | FALSE |
| 1,3,5-Trihydroxy-6,7-dimethoxy-2-methylanthraquinone | 0.131184314 | 0.183026912 | 0.72990062 | FALSE |
| 4',8-Dimethylgossypetin 3-glucoside | 0.405587431 | 0.462551957 | 0.727039942 | FALSE |
| Aklanonate | 0.010627632 | 0.022951162 | 0.71808358 | TRUE |
| Gallotannin | 0.017681787 | 0.035538642 | 0.71344153 | TRUE |
| Lappaol C | 0.399730244 | 0.461823162 | 0.71207751 | FALSE |
| 1,3,4,6-Tetra-O-galloyl glucose | 0.020453089 | 0.039731838 | 0.698289412 | TRUE |
| 3,3'-Bisjuglone | 0.137772807 | 0.189612745 | 0.696054315 | FALSE |
| 2, 4-Diacetylphloroglucinol | 0.312298137 | 0.377360248 | 0.693065774 | FALSE |
| 2-O-(4-Hydroxycinnamoyl)-1,6-di-O-galloyl-β-D-glucopyranose | 0.266037444 | 0.329302446 | 0.692051485 | FALSE |
| Rhaponticin | 0.147352867 | 0.201431865 | 0.688979469 | FALSE |
| Orientanol E; 3-(2,4-Dihydroxyphenyl)-5,7-dihydroxy-6,8-bis(3-methyl-2-buten-1-yl)-2,3-dihydro-4H-chromen-4-one | 0.160231042 | 0.216125591 | 0.681572703 | FALSE |
| [(2R,3R,4S,5R,6S)-3-Hydroxy-2-(hydroxymethyl)-5,6-bis[(3,4,5-trihydroxybenzoyl)oxy]oxan-4-yl] 3,4,5-trihydroxybenzoate | 0.61893913 | 0.656750588 | 0.679100487 | FALSE |
| p-Hydroxybenzyldesulphoglucosinolate | 0.204707787 | 0.263845593 | 0.678981334 | FALSE |
| 4''-O-Acetylafzelin | 0.214301731 | 0.274468463 | 0.678522195 | FALSE |
| 3,5,7-Trihydroxy-2-(3-hydroxy-4-methoxyphenyl)-6,8-dimethoxy-4H-chromen-4-one | 0.004211678 | 0.010754348 | 0.671012617 | TRUE |
| Osmanthuside B | 0.096952483 | 0.141592475 | 0.663277286 | FALSE |
| Silandrin | 0.101651483 | 0.146870115 | 0.660263922 | FALSE |
| 5,7,2'-Trihydroxy-3,6,8,4',5'-pentamethoxyflavone | 0.404248706 | 0.462353741 | 0.658391794 | FALSE |
| Viniferifuran | 0.550763164 | 0.593129561 | 0.652654166 | FALSE |
| 6'-Methoxypolygoacetophenoside | 0.634739567 | 0.669361725 | 0.648770548 | FALSE |
| 1-[2-(β-D-Glucopyranosyloxy)-4,6-dihydroxyphenyl]-3-(4-methoxyphenyl)-1-propanone | 0.81285465 | 0.82504747 | 0.647367646 | FALSE |
| Geshoidin | 0.104271759 | 0.150121753 | 0.637838128 | FALSE |
| 1-O-(3-Hydroxy-4,5-dimethoxybenzoyl)hexopyranose | 0.861571216 | 0.872313999 | 0.637828088 | FALSE |
| (2S,3S,4S,5R,6S)-3,4,5-Trihydroxy-6-[5-hydroxy-6-methoxy-2-(4-methoxyphenyl)-4-oxochromen-7-yl]oxyoxane-2-carboxylic acid | 0.324652831 | 0.391124775 | 0.632628277 | FALSE |
| [(2R,3S,4S,5R,6S)-6-[3,5-Dihydroxy-4-[3-(4-hydroxyphenyl)propanoyl]phenoxy]-3,4,5-trihydroxyoxan-2-yl]methyl 3,4,5-trihydroxybenzoate | 0.065250521 | 0.10512584 | 0.630273256 | FALSE |
| Gallic acid 4-O-(6-galloylglucoside) | 0.222263927 | 0.28111886 | 0.629305392 | FALSE |
| 6-Hydroxyluteolin 6,7-dimethyl ether 4'-glucoside | 0.289635416 | 0.354192708 | 0.626872854 | FALSE |
| Dehydrochorismic acid | 0.335772718 | 0.402134877 | 0.622740901 | FALSE |
| Fragransin C1 | 0.19319473 | 0.253022775 | 0.618060782 | FALSE |
| Mulberrofuran S | 0.245679357 | 0.307857466 | 0.61493387 | FALSE |
| Trilobatin | 0.457484361 | 0.506099865 | 0.611651305 | FALSE |
| (+)-Epicatechin | 4.29527E-05 | 0.000232517 | 0.611341112 | TRUE |
| Hesperetin 7-O-glucoside | 0.144077044 | 0.197619189 | 0.610019441 | FALSE |
| 3-(3,4-Dihydroxyphenyl)-1-propanol 3'-glucoside | 0.739599741 | 0.760196189 | 0.609184081 | FALSE |
| 4'-Methyl-(-)-epigallocatechin 3'-glucuronide | 0.70858883 | 0.730170216 | 0.606582383 | FALSE |
| Punicacortein B | 0.177704282 | 0.234246553 | 0.604664207 | FALSE |
| Myricitrin | 0.008880454 | 0.019701991 | 0.602763574 | TRUE |
| 2-(3,4,5-Trihydroxyphenyl)-3,4-dihydro-2H-1-benzopyran-3,4,5,7-tetrol | 0.22962917 | 0.288636046 | 0.601435084 | FALSE |
| m-Trigallic acid | 0.926998947 | 0.931588051 | 0.600989391 | FALSE |
| 5-Hydroxy pseudo baptigenin 7-O-glucoside | 0.078348205 | 0.120948179 | 0.600804863 | FALSE |
| 1,2',5-Tri-O-galloylhamamelofuranose | 0.071724493 | 0.112868776 | 0.596967906 | FALSE |
| Cimifugin | 0.475869521 | 0.525008221 | 0.596737312 | FALSE |
| 4-Methylumbelliferone | 0.293750982 | 0.357074547 | 0.583606351 | FALSE |
| Xanthohumol E | 0.295316551 | 0.357906029 | 0.582242107 | FALSE |
| Daphnoretin | 0.40753779 | 0.463151855 | 0.574916409 | FALSE |
| 5,7-Dihydroxy-2',6-dimethoxyisoflavone 7-rhamnoside | 0.504006771 | 0.55155458 | 0.572117908 | FALSE |
| Phlorizin | 0.065130658 | 0.10512584 | 0.571110469 | FALSE |
| Licoisoflavone A | 0.121427394 | 0.172217467 | 0.563758705 | FALSE |
| Flavanomarein | 0.36324424 | 0.427470033 | 0.561229698 | FALSE |
| 2-Protocatechoylphloroglucinolcarboxylate | 0.60396707 | 0.645291133 | 0.561063459 | FALSE |
| 3-(4-{[1,3-Dihydroxy-1-(4-hydroxy-3-methoxyphenyl)-2-propanyl]oxy}-3-methoxyphenyl)propyl 6-deoxy-α-L-mannopyranoside | 0.247195342 | 0.308804028 | 0.558902508 | FALSE |
| Parvisoflavone A | 0.261317069 | 0.325443957 | 0.55677596 | FALSE |
| 3,5-Dihydroxy-2-(4-hydroxyphenyl)-3,4-dihydro-2H-chromen-7-yl pentofuranoside | 0.063533136 | 0.104009892 | 0.55194707 | FALSE |
| 2,6-Digalloylglucose | 0.646013055 | 0.675982733 | 0.550809971 | FALSE |
| Rheidin B | 0.153631179 | 0.209309593 | 0.543332639 | FALSE |
| Pterocaryanin B | 0.082843908 | 0.125035786 | 0.530728858 | FALSE |
| Naringenin 7-O-β-D-glucoside | 0.404274823 | 0.462353741 | 0.529780266 | FALSE |
| [6-[(5,7-Dihydroxy-4-methyl-1,2,3,4-tetrahydronaphthalen-2-yl)oxy]-4,5-dihydroxy-3-[(E)-3-(4-hydroxyphenyl)prop-2-enoyl]oxyoxan-2-yl]methyl (E)-3-(4-hydroxyphenyl)prop-2-enoate | 0.456516513 | 0.506099865 | 0.519537938 | FALSE |
| Isochlorogenic acid b | 0.070589371 | 0.111950331 | 0.519198435 | FALSE |
| 3-Glucogallic acid | 0.592316273 | 0.634512946 | 0.518105098 | FALSE |
| 4',6'-Dihydroxy-2'-methoxyacetophenone 6'-glucoside | 0.431393686 | 0.483828278 | 0.511416493 | FALSE |
| Apigenin 7-(6''-crotonylglucoside) | 0.192519006 | 0.252953774 | 0.507784314 | FALSE |
| Quercetin-3-β-D-xyloside | 0.489447417 | 0.538524801 | 0.50751388 | FALSE |
| 4-Glucosyl gallate | 0.099017253 | 0.143575017 | 0.50461166 | FALSE |
| 5,7-Dihydroxy-2-(4-hydroxyphenyl)-3-[3,5,7-trihydroxy-2-(4-hydroxyphenyl)-3,4-dihydro-2H-chromen-8-yl]-2,3-dihydrochromen-4-one | 0.70449945 | 0.727803503 | 0.503059145 | FALSE |
| Catechin-4-ol 3-O-α-L-rhamnopyranoside | 0.897227815 | 0.90390693 | 0.500778033 | FALSE |
| (+/-)-Taxifolin | 0.018435927 | 0.036512128 | 0.495369103 | TRUE |
| 3-Feruloylquinic acid | 0.361156101 | 0.427470033 | 0.494000228 | FALSE |
| Isopulegone caffeate | 0.687458662 | 0.713831757 | 0.493949965 | FALSE |
| Catechin 3-O-gallate | 3.35428E-05 | 0.000187036 | 0.480232162 | TRUE |
| Taxifolin 3-arabinoside | 0.384151109 | 0.446943748 | 0.477806572 | FALSE |
| Okanin 4'-(2'',4'',6''-triacetylglucoside) | 0.20057369 | 0.260169067 | 0.471975047 | FALSE |
| Taxifolin | 0.78928982 | 0.805154942 | 0.470999087 | FALSE |
| Uralenneoside | 0.408394985 | 0.463151855 | 0.44962455 | FALSE |
| 1-(2,6-Dihydroxy-4-methoxyphenyl)ethanone | 0.387833241 | 0.449886559 | 0.438303484 | FALSE |
| 1-Hydroxyanthraquinone | 0.423149047 | 0.477218092 | 0.427066206 | FALSE |
| Rumexoside | 0.544162211 | 0.589146288 | 0.422341648 | FALSE |
| 3-(3,4-Dihydroxyphenyl)lactate | 0.4425411 | 0.493603534 | 0.419236881 | FALSE |
| Aromadendrin 4'-methyl ether 7-rhamnoside | 0.026652005 | 0.050095898 | 0.405430579 | FALSE |
| Piceatannol | 0.082200922 | 0.124994661 | 0.392736511 | FALSE |
| Naphthoherniarin | 0.878919086 | 0.887664549 | 0.383837784 | FALSE |
| Osajin | 0.639003071 | 0.672112038 | 0.377496835 | FALSE |
| Gnetin A | 0.70003061 | 0.725031704 | 0.358447854 | FALSE |
| 9-Hydroxy-4-methoxypsoralen 9-glucoside | 0.372189965 | 0.436731578 | 0.354429837 | FALSE |
| (-)-Epigallocatechin 3-p-coumaroate | 0.619545505 | 0.656750588 | 0.350143187 | FALSE |
| Luteolin-7-O-glucuronide | 0.781095342 | 0.798802793 | 0.326703688 | FALSE |
| Epigallo catechin | 0.005860512 | 0.014162904 | 0.309085731 | TRUE |
| Psoromic acid | 0.670631145 | 0.699938932 | 0.290597101 | FALSE |
| Hibiscetin | 0.775698914 | 0.79528727 | 0.283327422 | FALSE |
| Bisnorbadioquinone A | 0.078707228 | 0.121042177 | 0.230229709 | FALSE |


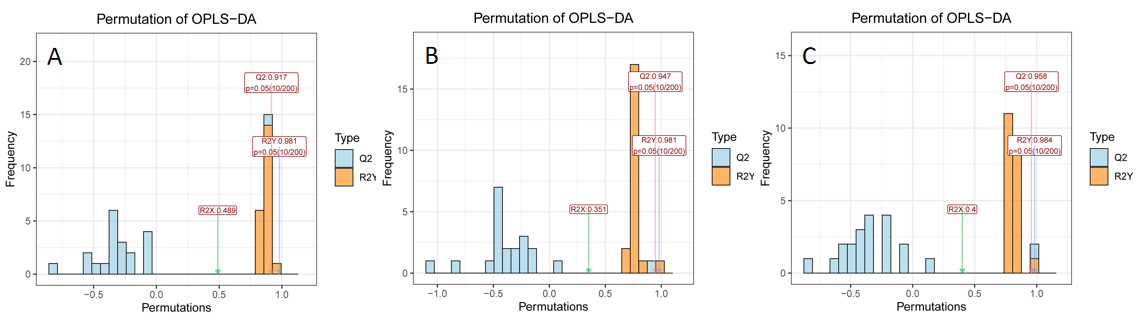


Figure S1 Permutation test results for the OPLS-DA models presented in Figure 5, validating the pairwise comparisons of walnut pellicle samples from different geographic origins. (A-C) Permutation plot for the BF vs YN, BF vs XJ and YN vs XJ comparison. Each plot displays the frequency distribution of Q2 and R^2^Y values across 200 permutations, with original model statistics indicated for reference.


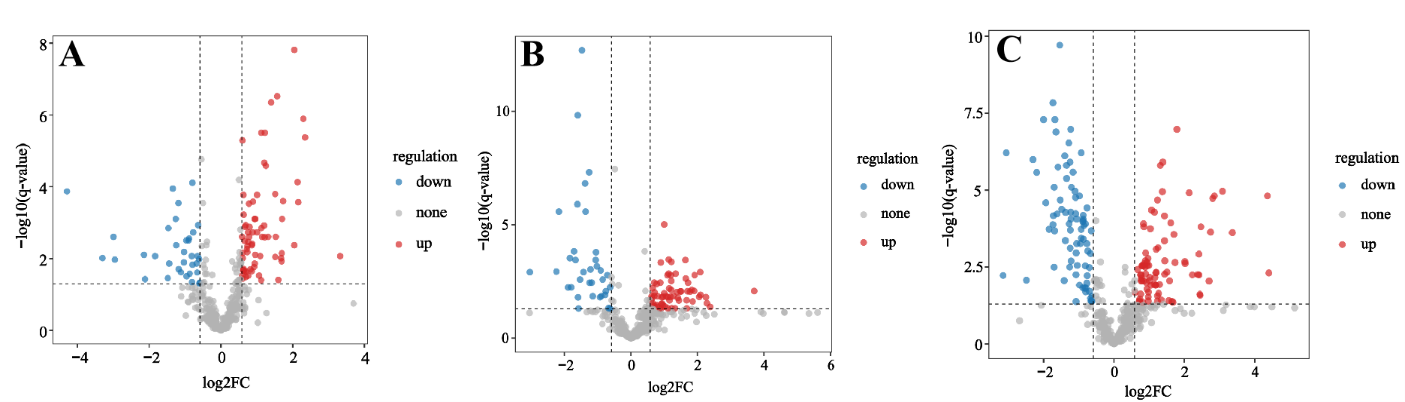


Figure S2 Volcano plots illustrating the differential abundance of metabolites in pairwise comparisons of walnut pellicle samples from the three geographic origins, based on thresholds of |log2(fold change)| > 0.585 (corresponding to >1.5-fold change) and FDR-adjusted q < 0.05.

(A) BF vs YN comparison, identifying 69 metabolites that were more abundant in YN than in BF (red), 35 metabolites that were more abundant in BF than in YN (blue), and 302 non-significant metabolites (gray). (B) BF vs XJ comparison, with 67 metabolites up-regulated in XJ, 35 down-regulated in XJ, and 304 non-significant metabolites. (C) YN vs XJ comparison, in which 86 metabolites showed higher levels in XJ than in YN, 80 were more abundant in YN, and 240 metabolites did not differ significantly. In all panels, the x-axis represents log₂(fold change) for the second region relative to the first, and the y-axis represents −log10(q-value). Vertical dashed lines mark the 1.5-fold change cut-offs (log2FC = ±0.585), and the horizontal dashed line marks the significance threshold (q = 0.05).

Using these FDR/FC criteria, 104 metabolites differed significantly between regions BF and YN. Region YN exhibited broad up-regulation relative to BF, with 69 compounds (66.3 %) present at ≥1.5-fold higher levels. Among the metabolites showing the largest fold increases in YN were 5,4′-dihydroxy-6,7,8,3′-tetramethoxyflavone 4′-galactoside, 4-(butoxymethyl)-2-methoxyphenol, 4,6,8-trihydroxy-7-methoxy-3-methyl-3,4-dihydro-1H-isochromen-1-one, a methyl-esterified hydroxy-phenyl glucoside iso-ellagitannin, 1-O-p-coumaroyl-β-D-glucose, phaseolic acid, edulisin I, 8-hydroxy-6,7-dimethoxy-2H-chromen-2-one, lyoniresinol-9′-sulfate and epigallocatechin-3-O-vanillate. Conversely, 35 metabolites (33.7 %) were relatively more abundant in BF; representative examples include daidzein, 8-C-glucosylnaringenin, isorhamnetin, trans-p-hydroxycinnamic acid, a highly methoxylated flavone, eriodictyol glycosides, frangulin B, a prenyl-benzoate glucoside, epigallocatechin-3-cinnamate and a highly galloylated glucose ester of pentahydroxybenzoic acid. This pattern indicates a shift from a tannin-dominated profile in BF to a more diverse flavonoid/phenolic spectrum in YN.

A total of 102 metabolites distinguished regions BF and XJ under the same FDR/FC thresholds. Region XJ showed extensive up-regulation of 67 compounds (65.7 %), while 35 metabolites were relatively depleted in XJ compared with BF. Metabolites displaying strong enrichment in XJ included pilosin, demethoxysudachitin, isoliquiritigenin, purpurin, loquatoside, dihydrokaempferol, the same hydroxy-phenyl glucoside iso-ellagitannin noted above, dihydroisorhamnetin, carnosol, viniferal and secoisolariciresinol. In contrast, metabolites that were comparatively suppressed in XJ comprised epigallocatechin-3-cinnamate, an acetyl-tri-glucosylated okanin, a tetrahydroxy-chromenone glucoside, catechin, ent-robinetinidol-3-O-gallate, epicatechin, prunin, apigenin-7-(ethylglucuronide), vescalagin and casuarinin. Overall, XJ walnuts replace the high ellagitannin load typical of BF with a more flavonoid- and stilbenoid-rich profile, consistent with their milder perceived astringency.

Region-specific divergence was also pronounced between YN and XJ, where 166 metabolites met the differential-expression criteria. Eighty-six compounds (51.8 %) were ≥1.5-fold higher in XJ than in YN, with viniferal, daidzein, 8-C-glucosylnaringenin, isorhamnetin, trans-p-hydroxycinnamic acid, eriodictyol, phloretin, 3-O-acetylpinobanksin, sinapyl alcohol and secoisolariciresinol among the most elevated. In contrast, 80 metabolites (48.2 %) were relatively depleted in XJ; the most affected included the tetramethoxyflavone galactoside, acetyl-tri-glucosylated okanin, melledonol, the tetrahydroxy-chromenone glucoside, 4-(butoxymethyl)-2-methoxyphenol, prunin, naringenin, lyoniresinol-9′-sulfate, a complex poly-galloylated ellagitannin (corilagin) and 1-O-p-coumaroyl-β-D-glucose. Thus, while YN retains the broadest overall polyphenol abundance, XJ distinguishes itself by selectively enriching specific flavonoids and stilbenoids at the expense of several ellagitannins.


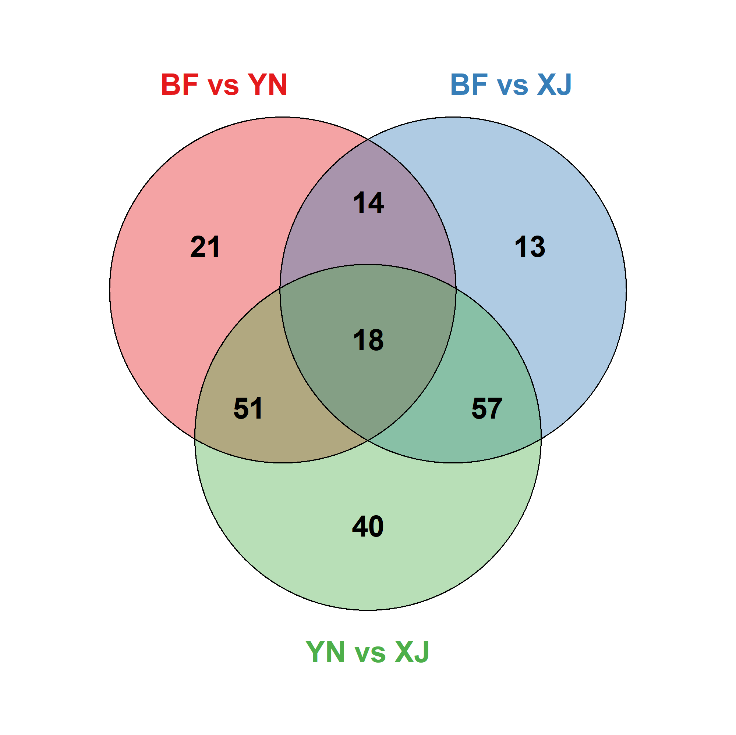


Figure S3 Venn diagram illustrating the overlaps among differential metabolites identified in pairwise comparisons of walnut pellicle samples from different geographic origins (BF vs YN, BF vs XJ, and YN vs XJ).

The Venn diagram in Figure S3 provides a visual summary of the overlaps and unique elements among the differential metabolites from the three pairwise comparisons. A total of 18 metabolites were shared across all comparisons, representing conserved differences likely driven by fundamental geographic or environmental factors affecting polyphenol biosynthesis. The largest pairwise overlap was observed between BF vs XJ and YN vs XJ (57 metabolites), suggesting greater similarity in metabolic perturbations between these pairs compared to others. In contrast, the BF vs YN and BF vs XJ overlap (14 metabolites) was the smallest, indicating more distinct profiles for BF relative to the other regions. Unique metabolites were most numerous in the YN vs XJ comparison (40), followed by BF vs YN (21) and BF vs XJ (13); nevertheless, the BF vs XJ contrast still contained several region-specific features, including ellagitannins and other high-molecular-weight tannins. These patterns emphasize the role of region-specific variations in flavonoid, phenolic acid, and tannin profiles, with implications for sensory attributes and authenticity markers.
